# Supplementary material for: Design, Synthesis and Characterization of HIV-1 CA-Targeting Small Molecules: Conformational Restriction of PF74
Source: Viruses. 2021 Mar 15;13(3):479. doi: 10.3390/v13030479 (PMC8000227; doi:10.3390/v13030479)
Supplement: Supplementary file 1 [file viruses-13-00479-s001.pdf]

Supporting Information

Design, synthesis and characterization of HIV-1 CA-targeting small molecules: conformational constraint of PF74

Rajkumar Lalji Sahani [1], Raquel Diana-Rivero [1], Sanjeev Kumar V. Vernekar [1], Lei Wang [1], Haijuan Du [2,3], Huanchun Zhang [2,3], Andres Emanuelli Castaner [2,3], Mary C. Casey [4], Karen A. Kirby [2,3], Philip R. Tedbury [2,3], Jiashu Xie [1], Stefan G. Sarafianos [2,3], and Zhengqiang Wang [1,]*

[1] Center for Drug Design, College of Pharmacy, University of Minnesota, Minneapolis, MN 55455, USA

[2] Laboratory of Biochemical Pharmacology, Department of Pediatrics, Emory University School of Medicine, Atlanta, GA 30322, USA

[3] Children's Healthcare of Atlanta, Atlanta, GA 300322, USA.

[4] Department of Molecular Microbiology and Immunology, University of Missouri School of Medicine, Christopher S. Bond Life Sciences Center, Columbia, MO 65211, USA

** Correspondence: wangx472@umn.edu; Tel: +1-612-626-7025*

Table of Contents

1. Scheme S1: Synthesis of intermediates **27-28** S2
2. Synthesis of compounds **1-3** and spectral data S3
3. Scheme S2. Synthesis of intermediates **29-37** and spectral data S5
4. Synthesis of compounds **4-8** and spectral data S10
5. Synthesis of compounds **9-19** and spectral data S14
6. Scheme S3. Synthesis of intermediates **39-41** and spectral data S20
7. Synthesis of compounds **20-25** and spectral data S22
8. NMR spectra of compounds **1-25** S25

Scheme S1. Synthesis of intermediates **27-28** and compounds **1-3**.

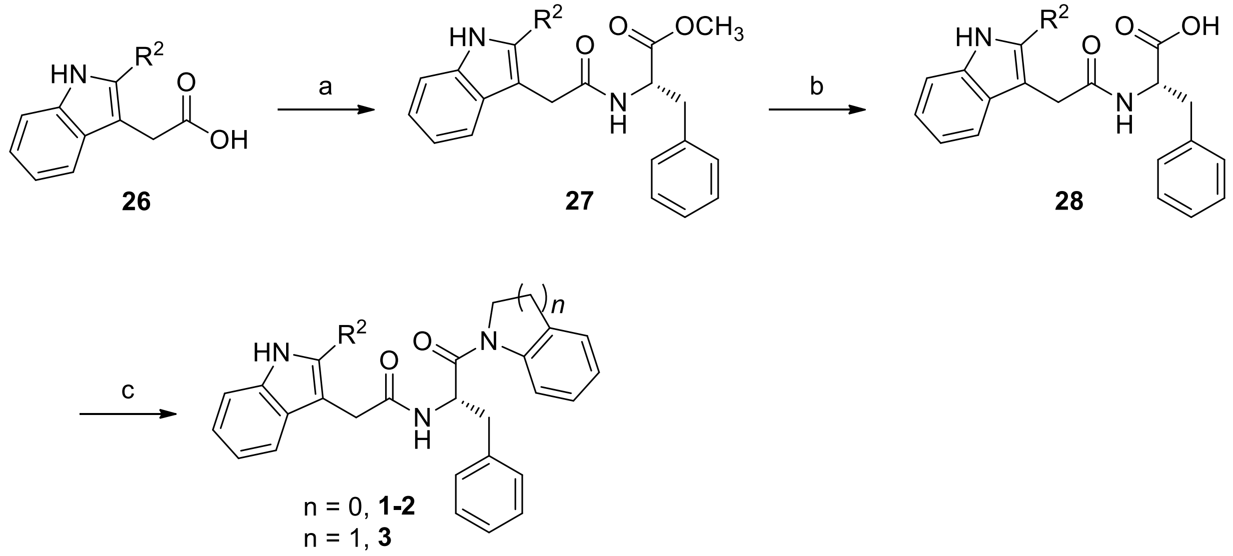

Reagents and conditions: a) L-Phenylalanine methyl ester hydrochloride, HATU, DIPEA, DMF, rt, 12 h; b) LiOH, THF, rt, 12 h; c) amine, PyOAP, DIPEA, DMF, rt, 12 h.

Synthesis of intermediate 27: To a solution of acid **26** (1.0 gm, 1 equiv.) in DMF (10 mL), HATU (1.2 equiv.) and DIPEA (2.0 equiv.) were added and the mixture was stirred at room temperature for 20 min. Followed by addition of desired *L*-Phenylalanine methyl ester hydrochloride (1.1 equiv.) and the mixture was further stirred at room overnight for 12 hours. Upon completion, H_2O (50 mL) was added and the reaction mixture was extracted with EtOAc (3x50 mL). The combined organic phases were washed with water, brine, dried over anhydrous Na_2SO_4, filtered and concentrated. The crude product was purified by Combi-flash on silica gel using 50-100% EtOAc/hexane.

Synthesis of intermediate 28: To a solution of the ester products **27**, obtained in the previous step, in methanol (10 mL) was added LiOH (3.0 equiv.) dissolved in H_2O (10 mL) and the mixture was stirred at room temperature for 12 hours. Upon completion, ethanol was evaporated and acidified

to pH = 3 using 1N HCl. Filtration of the crude product, washing, and drying gave the respective acids **28**.

Synthesis of compounds 1-3: To a solution of acid **28** (0.1 gm, 1 equiv.) in DMF (1 mL) PyOAP (1.2 equiv.) and DIPEA (2.0 equiv.) were added and the mixture was stirred at room temperature for 20 min. Followed by addition of desired indoline or tetrahydroquinoline (1.1 equiv.) and the mixture was further stirred at room overnight for 12 hours. Upon completion, H_2O (10 mL) was added and the reaction mixture was extracted with EtOAc (3x10 mL). The combined organic phases were washed with water, brine, dried over anhydrous Na_2SO_4, filtered and concentrated. The crude product was purified by Combi-flash on silica gel using 0-50% EtOAc/hexane.

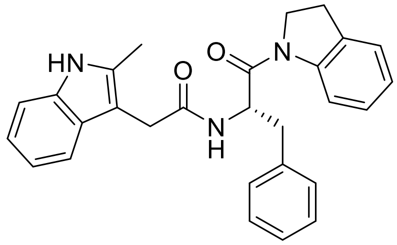

White solid, *(S)-N-(1-(indolin-1-yl)-1-oxo-3-phenylpropan-2-yl)-2-(2-methyl-1H-indol-3-yl)acetamide* (**1**)· Yield 81%. ^{1}H NMR (600 MHz, DMSO) δ 10.71 (s, 1H), 8.50 (d, *J* = 8.3 Hz, 1H), 8.09 (dd, *J* = 8.0, 3.1 Hz, 1H), 7.35 (dd, *J* = 7.9, 3.2 Hz, 1H), 7.27 (d, *J* = 7.3 Hz, 2H), 7.19 (ddd, *J* = 17.1, 14.0, 7.3 Hz, 6H), 7.00 (t, *J* = 7.4 Hz, 1H), 6.93 (t, *J* = 7.5 Hz, 1H), 6.82 (td, *J* = 7.4, 2.5 Hz, 1H), 4.74 (q, *J* = 5.7 Hz, 1H), 4.16 (q, *J* = 9.1 Hz, 1H), 3.90 (td, *J* = 9.9, 6.9 Hz, 1H), 3.49 (dd, *J* = 15.0, 3.1 Hz, 1H), 3.44 (dd, *J* = 15.1, 3.1 Hz, 1H), 3.09 (dd, *J* = 13.8, 6.0 Hz, 1H), 2.99 (q, *J* = 7.6 Hz, 2H), 2.90 (ddd, *J* = 13.5, 8.5, 2.8 Hz, 1H), 2.26 (d, *J* = 2.4 Hz, 3H). ^{13}C NMR (150 MHz, DMSO) δ 170.75, 169.81, 142.57, 137.44, 134.95, 132.92, 131.94, 129.27, 128.38, 128.09, 126.90, 126.40, 124.77, 123.61, 119.85, 118.01, 117.91, 116.31, 110.10, 104.73, 53.16,

47.29, 36.92, 30.93, 27.36, 11.31. HRMS (ESI) m/z calcd for $C_{28}H_{27}N_3O_2$ $[M - H]^-$ 436.2031, found 436.2034.

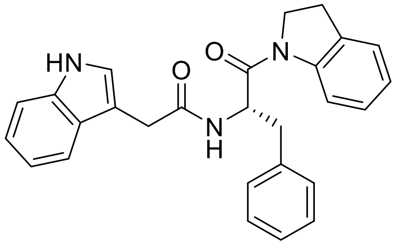

White solid, *(S)-2-(1H-indol-3-yl)-N-(1-(indolin-1-yl)-1-oxo-3-phenylpropan-2-yl)acetamide* (**2**). Yield 72%. ^{1}H NMR (600 MHz, DMSO) δ 10.81 (s, 1H), 8.59 (d, *J* = 7.8 Hz, 1H), 8.09 (d, *J* = 8.0 Hz, 1H), 7.42 (d, *J* = 7.9 Hz, 1H), 7.32 – 7.28 (m, 3H), 7.25 – 7.13 (m, 5H), 7.09 (d, *J* = 2.3 Hz, 1H), 7.01 (dt, *J* = 18.1, 7.4 Hz, 2H), 6.88 (t, *J* = 7.4 Hz, 1H), 4.78 (q, *J* = 7.6 Hz, 1H), 4.17 (td, *J* = 10.0, 6.6 Hz, 1H), 3.89 (td, *J* = 10.1, 6.8 Hz, 1H), 3.53 (s, 2H), 3.10 (dd, *J* = 13.5, 6.2 Hz, 1H), 2.99 (dp, *J* = 13.8, 8.1, 7.0 Hz, 2H), 2.90 (dd, *J* = 13.5, 8.3 Hz, 1H). ^{13}C NMR (151 MHz, DMSO) δ 170.63, 142.56, 137.43, 136.01, 131.95, 129.27, 128.11, 127.15, 126.90, 126.43, 124.78, 123.70, 123.62, 120.83, 118.70, 118.18, 116.32, 111.16, 108.65, 53.13, 47.31, 37.00, 32.12, 27.36. HRMS (ESI) m/z calcd for $C_{27}H_{25}N_3O_2$ $[M - H]^-$ 422.1874, found 422.1873.

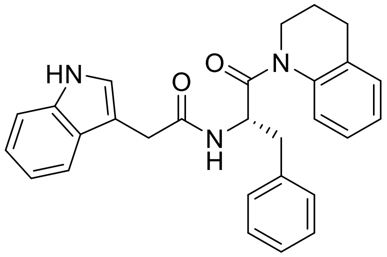

White solid, *(S)-N-(1-(3,4-dihydroquinolin-1(2H)-yl)-1-oxo-3-phenylpropan-2-yl)-2-(1H-indol-3-yl)acetamide* (**3**). Yield 65%. ^{1}H NMR (600 MHz, CD_3OD) δ 7.50 (d, *J* = 7.9 Hz, 1H), 7.35 (d, *J* = 8.1 Hz, 1H), 7.22 (d, *J* = 8.0 Hz, 1H), 7.14 – 7.05 (m, 8H), 7.01 (t, *J* = 7.5 Hz, 1H), 6.78 (s, 2H), 5.42 (s, 1H), 3.96 (s, 1H), 3.69 (s, 2H), 3.19 (s, 1H), 2.77 (s, 2H), 2.45 (d, *J* = 13.2 Hz, 1H), 1.98

(s, 1H), 1.71 (q, J = 9.4, 6.3 Hz, 1H), 1.56 (dt, J = 13.9, 7.0 Hz, 1H). ^{13}C NMR (150 MHz, CD_3OD) δ 172.88, 171.62, 137.84, 136.74, 136.24, 128.63, 128.01, 127.06, 126.40, 125.96, 124.50, 123.57, 121.19, 118.62, 118.04, 110.92, 107.83, 51.72, 42.74, 38.45, 32.35, 25.61, 23.36 (three carbons are merging). HRMS (ESI) m/z calcd for $C_{28}H_{27}N_3O_2$ $[M - H]^-$ 436.2031, found 436.2037.

Scheme S2. Synthesis of intermediates **30-37** and compounds **4-19**.

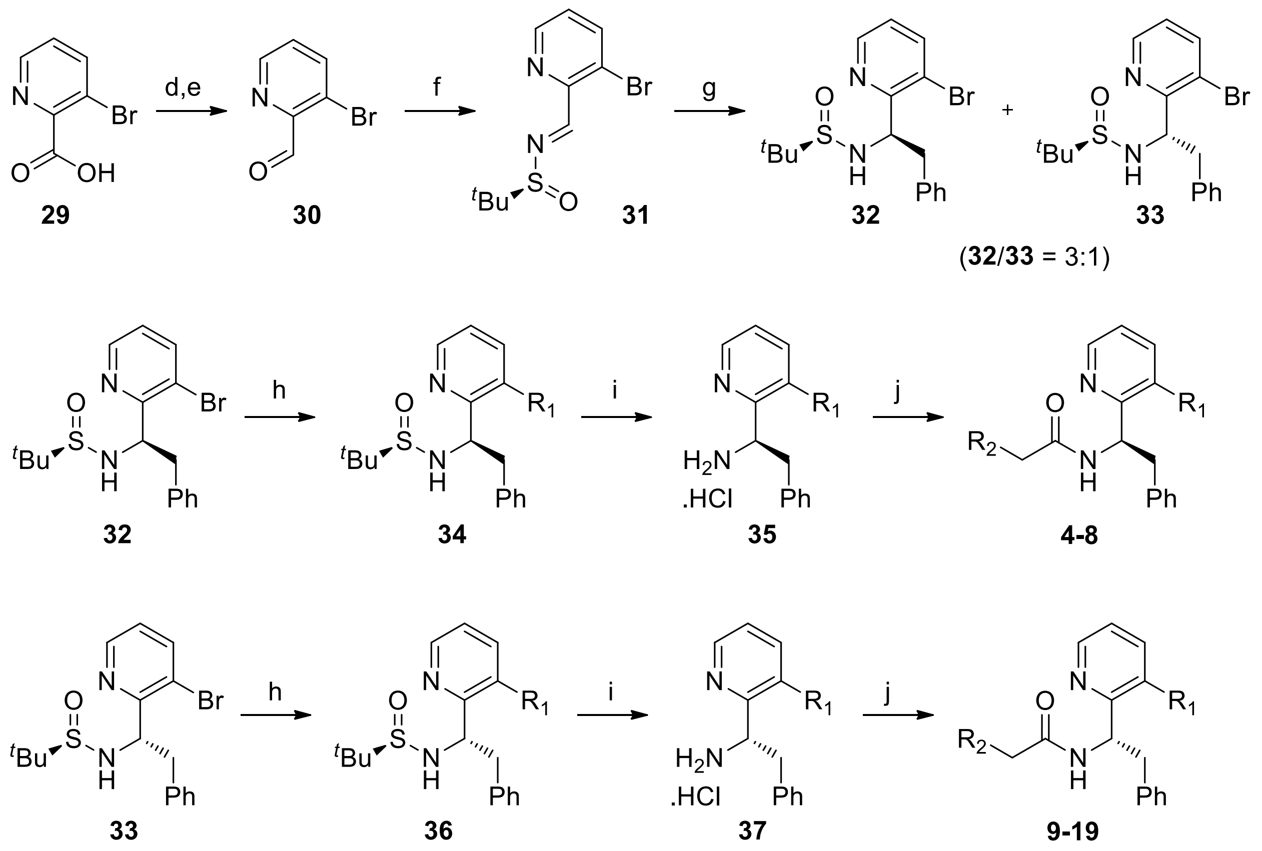

Reagents and conditions: d) $(COCl)_2$, cat. DMF, DCM, 0 °C - rt, 2 h, then MeOH, rt, 2 h; e) DIBAL-H, THF, -78 °C, 3 h; f) (R)-(+)-2-Methyl-2-propanesulfinamide, $CuSO_4$, DCM, rt, 3 h; g) Benzylmagnesium chloride, DCM, -78 °C - rt, 5 h; h) boronic acid, $Pd(PPh_3)_2Cl$, K_2CO_3, DME, MW, 120 °C, 45 min.; i) 4N HCl/dioxane, MeOH, rt, 1 h; j) acid, HATU or T3P, DIPEA, DMF, rt, 12 h.

Synthesis of intermediate 30:

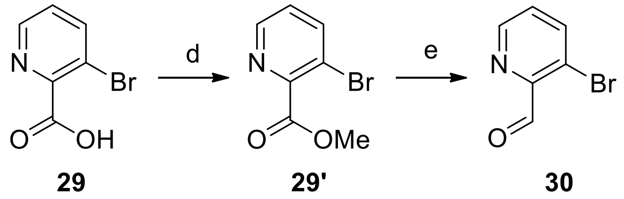

Reagents and conditions: d) $(COCl)_2$, cat. DMF, DCM, 0 °C - rt, 2 h, then MeOH, rt, 2 h; e) DIBAL-H, THF, -78 °C, 3 h

Synthesis of intermediate 29': To a solution of acid **29** (2.0 gm, 1 equiv.) in DCM (50 mL), oxalyl chloride (1.2 equiv.) and catalytic amount of DMF were slowly added at 0 °C and the mixture was stirred at room temperature for 2 hours. Upon completion, indicated by TLC, the reaction mixture was evaporated to dryness, MeOH (20 mL) was added, and the reaction was stirred at room temperature for another 2 hours. Upon completion, the reaction mixture was evaporated to dryness, sat. $NaHCO_3$ (10 mL) was added and the reaction mixture was extracted with EtOAc (3x50 mL). The combined organic phases were washed with water, brine, dried over anhydrous Na_2SO_4, filtered and concentrated. The crude product was purified by Combi-flash on silica gel using 5-10% EtOAc/hexane to get the desired ester **29'** as yellow oil.

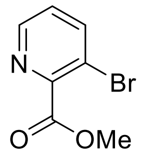

^{1}H NMR (600 MHz, $CDCl_3$) δ 8.61 (ddt, J = 4.6, 3.2, 1.7 Hz, 1H), 8.00 (ddt, J = 6.9, 3.1, 1.6 Hz, 1H), 7.30 (dtd, J = 8.0, 3.1, 1.7 Hz, 1H), 4.01 (s, 3H).

Synthesis of intermediate 30: To a solution of ester **29'** (1.0 gm, 1 equiv.) in dry THF (10 mL), DIBAL-H (1.5 equiv.) was added at -78 °C over 40 minute and the mixture was maintained at the same temperature for 2.5 hours. Upon completion, indicated by TLC, MeOH (10 mL) and saturated $NaHCO_3$ (10 mL) were added and the reaction mixture was extracted with EtOAc (3x50

mL). The combined organic phases were washed with water, brine, dried over anhydrous Na_2SO_4, filtered and concentrated. The crude product was purified by Combi-flash on silica gel using 2-20% EtOAc/hexane to get the desired aldehyde **30** as white solid.

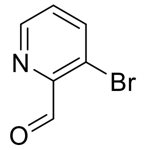

^{1}H NMR (600 MHz, $CDCl_3$) δ 10.25 (d, *J* = 1.1 Hz, 1H), 8.76 (d, *J* = 4.5 Hz, 1H), 8.04 (dt, *J* = 8.1, 1.3 Hz, 1H), 7.37 (ddd, *J* = 8.2, 4.5, 1.0 Hz, 1H).

Synthesis of intermediate 31: To a solution of aldehyde **30** (1.0 gm, 1 equiv.) in DCM (20 mL), (*R*)-(+)-2-Methyl-2-propanesulfinamide (1.1 equiv.) and anhydrous $CuSO_4$ (2.0 equiv.) were added and the mixture was stirred at room temperature for 3 hours. Upon completion, indicated by TLC, the reaction mixture was filtered through short celite bed and evaporated to dryness. The crude product was purified by Combi-flash on silica gel using 15-30% EtOAc/hexane to get the desired sulfinimine **31** as white solid.

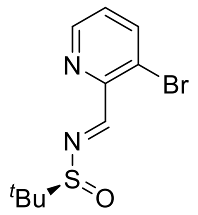

^{1}H NMR (600 MHz, $CDCl_3$) δ 9.03 (d, *J* = 1.2 Hz, 1H), 8.73 (dd, *J* = 4.5, 1.4 Hz, 1H), 8.00 (dt, *J* = 8.2, 1.3 Hz, 1H), 7.30 – 7.24 (m, 1H), 1.31 (s, 9H).

Synthesis of intermediate 32** and **33: To a solution of sulfinimine **31** (1.0 gm, 1 equiv.) in dry DCM (20 mL), benzylmagnesium chloride (1.4M in THF, 1.2 equiv.) was added at -78 °C over 20 minute and the mixture was maintained at the same temperature for 2.5 hours. Another portion of

benzylmagnesium bromide (1.4M in THF, O.2 equiv.) was added at -78 °C and the reaction mixture was warmed to room temperature for an hour. Upon completion, indicated by TLC, saturated NH_4Cl (20 mL) was added and the reaction mixture was extracted with EtOAc (3x50 mL). The combined organic phases were washed with water, brine, dried over anhydrous Na_2SO_4, filtered and concentrated. The crude product was purified by Combi-flash on silica gel using 20-22% EtOAc/hexane to get the desired intermediate **33** as brown solid and 30-50% EtOAc/hexane to get the desired intermediate **32** as brown oil.

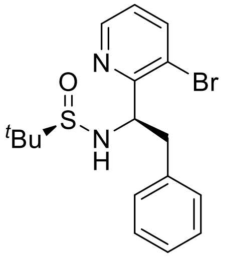

32, ^{1}H NMR (400 MHz, DMSO) δ 8.61 (dd, *J* = 4.6, 1.4 Hz, 1H), 8.06 (dd, *J* = 8.1, 1.4 Hz, 1H), 7.47 – 6.32 (m, 6H), 4.93 (td, *J* = 9.2, 5.2 Hz, 1H), 3.03 (dd, *J* = 13.5, 5.1 Hz, 1H), 2.91 (dd, *J* = 13.4, 9.0 Hz, 1H), 0.96 (s, 9H). ^{13}C NMR (100 MHz, DMSO) δ 158.91, 147.96, 140.69, 138.04, 129.47, 127.98, 126.25, 124.35, 119.36, 60.80, 55.40, 42.00, 22.23.

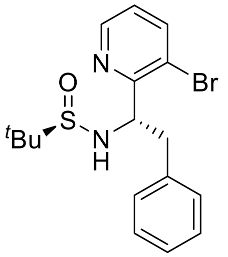

33, ^{1}H NMR (400 MHz, DMSO) δ 8.59 (dd, *J* = 4.6, 1.5 Hz, 1H), 7.94 (dd, *J* = 8.1, 1.5 Hz, 1H), 7.27 – 7.09 (m, 4H), 7.13 – 7.01 (m, 2H), 5.63 (d, *J* = 8.3 Hz, 1H), 5.08 (td, *J* = 8.2, 6.3 Hz, 1H), 3.27 (dd, *J* = 13.2, 8.2 Hz, 1H), 3.13 (dd, *J* = 13.2, 6.3 Hz, 1H), 0.97 (s, 9H). ^{13}C NMR (100 MHz,

DMSO) δ 158.28, 147.95, 140.43, 137.62, 129.45, 128.02, 126.23, 124.15, 120.46, 58.82, 55.60, 41.49, 22.28.

Synthesis of intermediate 34: To a solution of intermediate **32** (0.2 gm, 1 equiv.) in DME (7 mL) in a microwave vial, corresponding boronic acid (1.05 equiv.), 2M K_2CO_3 in 3 mL water (10.0 equiv.), and Pd catalyst were added and the mixture was irradiated in microwave at 120 °C for 45 mins. Upon completion, indicated by TLC, the reaction mixture was filtered through short celite bed, diluted with water and extracted with EtOAc (3x20 mL). The combined organic phases were washed with water, brine, dried over anhydrous Na_2SO_4, filtered and concentrated. The crude product was purified by Combi-flash on silica gel using 80-95% EtOAc/hexane to get the desired Suzuki coupling intermediate **34** as colorless oil.

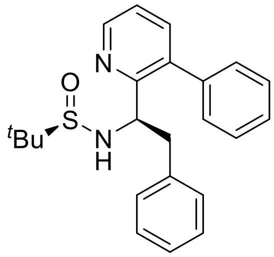

^{1}H NMR (600 MHz, $CDCl_3$) δ 8.49 (dt, J = 4.8, 1.5 Hz, 1H), 7.57 – 7.50 (m, 1H), 7.32 (ddt, J = 9.4, 7.6, 2.1 Hz, 2H), 7.26 (d, J = 6.2 Hz, 2H), 7.09 (ddd, J = 7.6, 4.8, 1.2 Hz, 1H), 6.97 (dq, J = 10.8, 5.7, 4.8 Hz, 4H), 6.58 (dt, J = 7.7, 1.4 Hz, 2H), 4.79 (d, J = 9.0 Hz, 1H), 4.63 – 4.56 (m, 1H), 2.81 (dd, J = 13.4, 6.6 Hz, 1H), 2.70 (dd, J = 13.4, 8.1 Hz, 1H), 0.97 (d, J = 1.3 Hz, 9H).

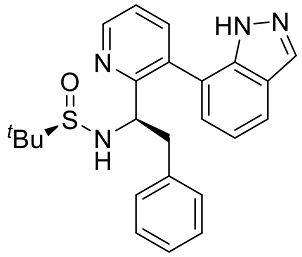

^{1}H NMR (600 MHz, $CDCl_3$) δ 8.74 – 8.70 (m, 1H), 8.14 (s, 1H), 7.75 (d, *J* = 8.1 Hz, 1H), 7.44 (dt, *J* = 7.4, 1.4 Hz, 1H), 7.32 – 7.15 (m, 1H), 7.12 (t, *J* = 7.4 Hz, 1H), 7.05 (t, *J* = 7.5 Hz, 2H), 7.01 (t, *J* = 7.5 Hz, 1H), 6.55 (d, *J* = 7.5 Hz, 2H), 6.32 (d, *J* = 6.9 Hz, 1H), 5.43 (d, *J* = 10.0 Hz, 1H), 4.29 (dt, *J* = 10.0, 7.5 Hz, 1H), 2.93 – 2.84 (m, 2H), 1.23 (s, 9H).

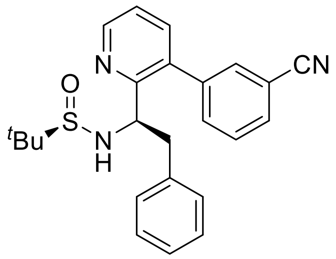

^{1}H NMR (600 MHz, $CDCl_3$) δ 8.71 (dd, *J* = 4.7, 1.7 Hz, 1H), 7.73 – 7.57 (m, 1H), 7.48 (d, *J* = 6.5 Hz, 2H), 7.31 (dd, *J* = 7.6, 1.7 Hz, 1H), 7.26 – 7.20 (m, 3H), 7.13 (t, *J* = 7.6 Hz, 2H), 6.60 (dd, *J* = 7.9, 1.4 Hz, 2H), 4.87 (d, *J* = 9.3 Hz, 1H), 4.47 (td, *J* = 9.3, 6.0 Hz, 1H), 3.00 (qd, *J* = 12.9, 7.6 Hz, 2H), 1.20 (s, 9H).

Synthesis of intermediate 35: To a solution of intermediate **34** (0.2 gm, 1 equiv.) in MeOH (10 mL), 4N HCl/dioxane (36.0 equiv.) was added and the mixture was stirred at room temperature for an hour. Upon completion, indicated by TLC, the reaction mixture evaporated to dryness and used in for next step without further purification.

Synthesis of compounds 4-8: To a solution of amine hydrochloride **35** (0.1 gm, 1 equiv.) in DMF (1 mL) HATU (1.2 equiv.), DIPEA (2.0 equiv.) and desired acids (1.1 equiv.) were added and the mixture was stirred at room temperature for 12 hours. Upon completion, H_2O (10 mL) was added and the reaction mixture was extracted with EtOAc (3x10 mL). The combined organic phases were washed with water, brine, dried over anhydrous Na_2SO_4, filtered and concentrated. The crude product was purified by Combi-flash on silica gel using 0-50% EtOAc/hexane.

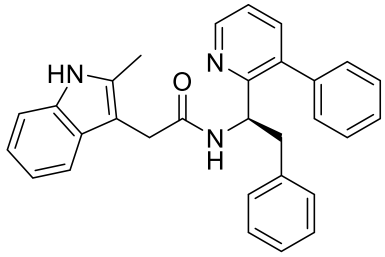

White solid, *(R)-2-(2-methyl-1H-indol-3-yl)-N-(2-phenyl-1-(3-phenylpyridin-2-yl)ethyl)acetamide* (**4**). Yield 74%. ^{1}H NMR (400 MHz, $CDCl_3$) δ 8.27 (d, *J* = 4.7 Hz, 1H), 8.15 – 8.03 (m, 1H), 7.35 – 7.25 (m, 5H), 7.20 – 7.15 (m, 1H), 7.05 (q, *J* = 7.9, 6.5 Hz, 2H), 6.96 (dd, *J* = 7.5, 5.2 Hz, 4H), 6.88 (t, *J* = 7.4 Hz, 2H), 6.77 (d, *J* = 8.6 Hz, 1H), 6.42 (d, *J* = 7.4 Hz, 2H), 5.49 (q, *J* = 7.5 Hz, 1H), 3.53 (d, *J* = 1.9 Hz, 2H), 2.71 (dd, *J* = 12.7, 6.8 Hz, 1H), 2.62 (dd, *J* = 13.2, 6.8 Hz, 1H), 2.19 (s, 3H). ^{13}C NMR (100 MHz, $CDCl_3$) δ 170.64, 157.05, 147.96, 138.28, 137.74, 137.18, 136.84, 135.51, 133.30, 129.37, 129.23, 128.6, 128.5 128.05, 127.75, 126.20, 122.04, 121.49, 119.75, 118.17, 110.43, 105.11, 51.66, 42.08, 32.53, 11.74. HRMS (ESI) m/z calcd for $C_{30}H_{27}N_3O$ $[M - H]^+$ 446.2227, found 446.2227.

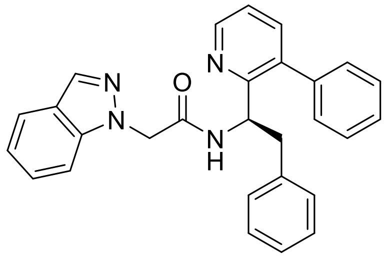

White solid, *(R)-2-(1H-indazol-1-yl)-N-(2-phenyl-1-(3-phenylpyridin-2-yl)ethyl)acetamide* (**5**). Yield 32%. ^{1}H NMR (400 MHz, $CDCl_3$) δ 8.36 (dd, J = 4.8, 1.7 Hz, 1H), 8.11 (s, 1H), 7.76 (d, J = 8.1 Hz, 1H), 7.41 – 7.26 (m, 6H), 7.28 – 7.05 (m, 3H), 7.05 – 6.87 (m, 5H), 6.47 (d, J = 7.3 Hz, 2H), 5.52 (q, J = 7.4 Hz, 1H), 5.00 (d, J = 2.3 Hz, 2H), 2.78 (dd, J = 13.2, 7.2 Hz, 1H), 2.70 (dd, J = 13.2, 6.8 Hz, 1H). ^{13}C NMR (100 MHz, $CDCl_3$) δ 166.54, 156.25, 148.04, 140.33, 138.06,

137.89, 136.84, 136.82, 135.05, 129.33, 129.14, 128.59, 128.08, 127.83, 127.16, 126.30, 124.43, 122.23, 121.37, 109.22, 52.44, 51.79, 42.20. HRMS (ESI) m/z calcd for $C_{28}H_{24}N_4O$ [M − H]$^-$ 431.1877, found 431.1881.

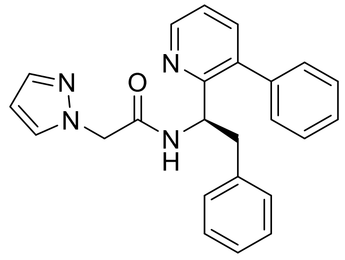

White solid, *(R)-N-(2-phenyl-1-(3-phenylpyridin-2-yl)ethyl)-2-(1H-pyrazol-1-yl)acetamide* (**6**). Yield 25%. ^{1}H NMR (400 MHz, $CDCl_3$) δ 8.36 (d, *J* = 4.8 Hz, 1H), 7.57 – 7.47 (m, 2H), 7.43 – 7.24 (m, 4H), 7.17 (q, *J* = 5.9, 4.4 Hz, 3H), 7.06 (dd, *J* = 7.7, 4.8 Hz, 1H), 6.95 – 6.82 (m, 4H), 6.43 (d, *J* = 7.3 Hz, 2H), 6.18 (s, 1H), 5.38 (q, *J* = 7.4 Hz, 1H), 4.63 (d, *J* = 3.0 Hz, 2H), 2.80 – 2.62 (m, 2H). ^{13}C NMR (100 MHz, $CDCl_3$) δ 166.28, 156.10, 147.95, 140.94, 138.01, 136.81, 132.17, 132.07, 130.92, 129.31, 129.03, 128.47, 128.08, 127.73, 126.31, 122.31, 106.65, 55.10, 51.88, 42.20. HRMS (ESI) m/z calcd for $C_{24}H_{22}N_4O$ [M − H]$^-$ 381.1721, found 381.1726.

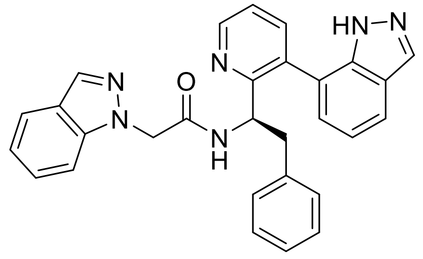

White solid, *(R)-N-(1-(3-(1H-indazol-7-yl)pyridin-2-yl)-2-phenylethyl)-2-(1H-indazol-1-yl)acetamide* (**7**). Yield 40%. ^{1}H NMR (400 MHz, $CDCl_3$) δ 12.57 (bs, 1H), 8.56 (d, *J* = 4.8 Hz, 1H), 8.12 (s, 2H), 7.77 (dd, *J* = 8.1, 4.7 Hz, 2H), 7.53 (d, *J* = 7.7 Hz, 1H), 7.45 – 7.34 (m, 2H), 7.31 – 7.27 (m, 1H), 7.22 (t, *J* = 7.2 Hz, 1H), 7.11 – 6.95 (m, 3H), 6.87 (d, *J* = 7.7 Hz, 2H), 6.62 (d, *J* = 7.0 Hz, 1H), 6.26 (d, *J* = 7.5 Hz, 2H), 5.16 – 5.01 (m, 2H), 4.94 – 4.87 (m, 1H), 2.71 (dt, *J*

= 17.0, 8.3 Hz, 2H). ^{13}C NMR (100 MHz, $CDCl_3$) δ 168.85, 157.23, 148.84, 140.42, 139.68, 137.76, 135.80, 135.28, 135.08, 133.56, 128.84, 128.20, 127.99, 127.37, 126.73, 124.34, 123.57, 122.92, 121.56, 121.34, 121.20, 120.95, 120.70, 109.36, 54.81, 52.06, 41.71. HRMS (ESI) m/z calcd for $C_{29}H_{24}N_6O$ $[M - H]^-$ 471.1939, found 471.1942.

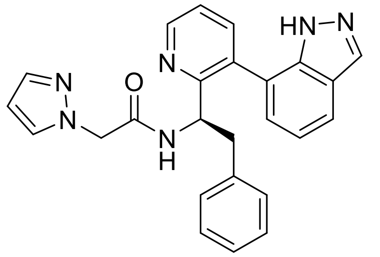

White solid, *(R)-N-(1-(3-(1H-indazol-7-yl)pyridin-2-yl)-2-phenylethyl)-2-(1H-pyrazol-1-yl)acetamide* (**8**). Yield 31%. ^{1}H NMR (400 MHz, $CDCl_3$) δ 12.48 (bs, 1H), 8.69 (d, *J* = 4.9 Hz, 1H), 8.12 (s, 1H), 7.79 (d, *J* = 8.1 Hz, 1H), 7.70 – 7.59 (m, 2H), 7.51 (d, *J* = 2.3 Hz, 1H), 7.46 – 7.35 (m, 2H), 7.08 (q, *J* = 7.4 Hz, 2H), 6.98 (d, *J* = 7.7 Hz, 2H), 6.59 (d, *J* = 7.0 Hz, 1H), 6.39 (d, *J* = 7.5 Hz, 2H), 6.33 (s, 1H), 4.95 – 4.83 (m, 3H), 2.83 (d, *J* = 7.7 Hz, 2H). ^{13}C NMR (100 MHz, $CDCl_3$) δ 168.72, 156.88, 147.80, 141.24, 140.96, 137.52, 135.59, 135.10, 134.32, 131.52, 128.99, 128.59, 128.07, 126.95, 123.62, 123.34, 121.27, 120.01, 106.78, 54.83, 41.63, 29.83. HRMS (ESI) m/z calcd for $C_{25}H_{22}N_6O$ $[M - H]^-$ 421.1782, found 421.1786.

Synthesis of intermediate 36: Intermediate **36** was synthesized from intermediate **33** as per the synthetic protocol described for the synthesis of intermediate **34**.

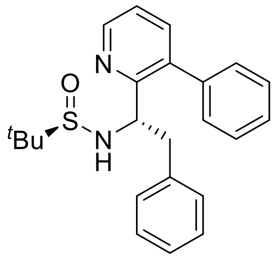

^{1}H NMR (600 MHz, $CDCl_3$) δ 8.66 (dd, *J* = 4.9, 1.8 Hz, 1H), 7.67 (ddt, *J* = 12.1, 6.9, 1.4 Hz, 2H), 7.58 – 7.52 (m, 1H), 7.46 (ddd, *J* = 8.7, 7.0, 2.8 Hz, 2H), 7.36 – 7.25 (m, 3H), 7.13 – 7.08 (m, 1H), 7.08 – 7.03 (m, 2H), 6.77 (s, 1H), 6.68 – 6.64 (m, 2H), 4.80 – 4.76 (m, 1H), 3.21 (d, *J* = 7.5 Hz, 2H), 1.13 (s, 9H).

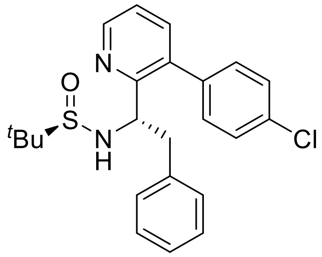

^{1}H NMR (600 MHz, $CDCl_3$) δ 8.68 (d, *J* = 4.8 Hz, 1H), 7.36 (s, 1H), 7.25 (d, *J* = 9.4 Hz, 4H), 7.14 – 7.09 (m, 1H), 7.09 – 7.00 (m, 2H), 6.65 (d, *J* = 7.5 Hz, 4H), 4.71 (d, *J* = 8.0 Hz, 1H), 3.23 (d, *J* = 7.7 Hz, 2H), 1.18 – 1.10 (m, 9H).

Synthesis of intermediate 37: Intermediate **37** was synthesized from intermediate **36** as per the synthetic protocol described for the synthesis of intermediate **35**.

Synthesis of compounds 9-19: Compounds **9-19** were synthesized by HATU or T_3P coupling of desired acids and intermediate **37** following a process described for the synthesis of compounds **4-8**. T_3P was used in place of HATU for the synthesis of compound **15**.

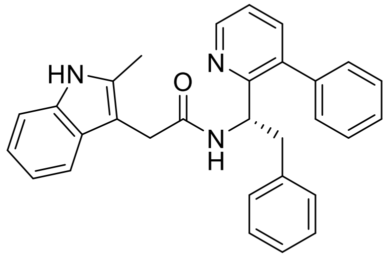

White solid, *(S)-2-(2-methyl-1H-indol-3-yl)-N-(2-phenyl-1-(3-phenylpyridin-2-yl)ethyl)acetamide* (**9**). Yield 62%. ^{1}H NMR (400 MHz, $CDCl_3$) δ 8.29 (dd, *J* = 4.8, 1.6 Hz, 1H), 8.20 (s, 1H), 7.38 –

7.25 (m, 5H), 7.22 – 7.16 (m, 1H), 7.12 – 7.01 (m, 2H), 7.04 – 6.94 (m, 4H), 6.90 (t, J = 7.4 Hz, 2H), 6.81 (d, J = 8.6 Hz, 1H), 6.44 (d, J = 7.3 Hz, 2H), 5.52 (q, J = 7.4 Hz, 1H), 3.55 (d, J = 1.7 Hz, 2H), 2.78 – 2.60 (m, 2H), 2.19 (s, 3H). ^{13}C NMR (100 MHz, $CDCl_3$) δ 170.75, 156.99, 147.83, 138.18, 137.90, 137.12, 136.91, 135.52, 133.36, 129.34, 129.21, 128.6, 128.5 128.07, 127.78, 126.22, 122.10, 121.43, 119.69, 118.11, 110.47, 104.97, 51.70, 42.05, 32.52, 11.70. HRMS (ESI) m/z calcd for $C_{30}H_{27}N_3O$ $[M-H]^-$ 444.2081, found 444.2083.

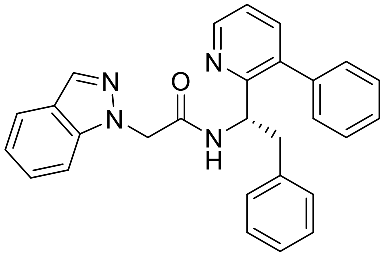

White solid, *(S)-2-(1H-indazol-1-yl)-N-(2-phenyl-1-(3-phenylpyridin-2-yl)ethyl)acetamide* (**10**). Yield 50%. ^{1}H NMR (400 MHz, $CDCl_3$) δ 8.39 (d, J = 4.9 Hz, 1H), 8.12 (s, 1H), 7.77 (d, J = 8.1 Hz, 1H), 7.47 – 7.14 (m, 8H), 7.10 – 6.89 (m, 6H), 6.48 (d, J = 7.5 Hz, 2H), 5.53 (q, J = 7.4 Hz, 1H), 5.02 (d, J = 1.8 Hz, 2H), 2.85 – 2.68 (m, 2H). ^{13}C NMR (100 MHz, $CDCl_3$) δ 166.67, 156.12, 147.77, 140.33, 138.25, 137.86, 136.94, 136.72, 135.06, 129.29, 129.11, 128.63, 128.12, 128.0, 127.9, 127.18, 126.36, 124.40, 122.38, 121.38, 109.20, 52.37, 51.83, 42.08. HRMS (ESI) m/z calcd for $C_{28}H_{24}N_4O$ $[M-H]^-$ 431.1877, found 431.1878.

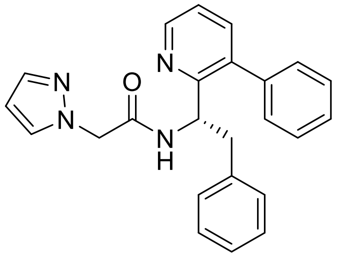

White solid, *(S)-N-(2-phenyl-1-(3-phenylpyridin-2-yl)ethyl)-2-(1H-pyrazol-1-yl)acetamide* (**11**). Yield 58%. ^{1}H NMR (400 MHz, $CDCl_3$) δ 8.46 (dd, J = 4.9, 1.7 Hz, 1H), 7.67 – 7.57 (m, 2H), 7.52

– 7.40 (m, 1H), 7.39 (ddd, J = 13.2, 6.7, 2.1 Hz, 2H), 7.35 (d, J = 2.6 Hz, 2H), 7.28 (p, J = 3.6 Hz, 3H), 7.24 – 7.11 (m, 2H), 7.07 – 6.87 (m, 5H), 6.56 – 6.49 (m, 2H), 6.29 (t, J = 2.2 Hz, 1H), 5.47 (q, J = 7.5 Hz, 1H), 4.73 (d, J = 3.6 Hz, 2H), 2.87 – 2.72 (m, 2H). ^{13}C NMR (100 MHz, $CDCl_3$) δ 166.23, 156.22, 148.14, 141.02, 137.86, 136.91, 132.23, 130.92, 129.39, 129.09, 128.65, 128.51, 128.11, 127.75, 126.33, 122.26, 106.73, 55.23, 51.88, 42.34. HRMS (ESI) m/z calcd for $C_{24}H_{22}N_4O$ $[M-H]^-$ 381.1721, found 381.1725.

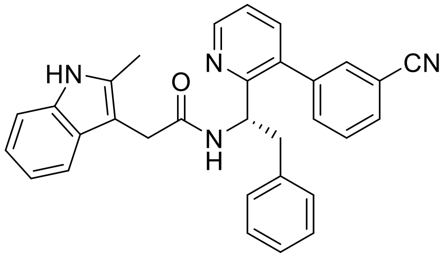

White solid, *(S)-N-(1-(3-(3-cyanophenyl)pyridin-2-yl)-2-phenylethyl)-2-(2-methyl-1H-indol-3-yl)acetamide* (**12**). Yield 44%. ^{1}H NMR (400 MHz, $CDCl_3$) δ 8.50 (dd, *J* = 4.9, 1.7 Hz, 1H), 8.06 (s, 1H), 7.59 (d, *J* = 7.6 Hz, 1H), 7.42 (dd, *J* = 30.9, 7.8 Hz, 3H), 7.30 (d, *J* = 8.0 Hz, 2H), 7.15 (dt, *J* = 17.8, 7.5 Hz, 3H), 7.02 (q, *J* = 7.5 Hz, 4H), 6.62 (s, 1H), 6.47 (d, *J* = 7.6 Hz, 2H), 5.30 (q, *J* = 8.1 Hz, 1H), 3.73 – 3.58 (m, 2H), 2.92 – 2.86 (m, 2H), 2.39 (s, 3H). ^{13}C NMR (100 MHz, $CDCl_3$) δ 171.22, 156.77, 136.47, 135.51, 133.76, 133.42, 132.68, 131.50, 129.30, 129.14, 128.53, 128.48, 126.90, 122.75, 121.57, 119.82, 118.45, 118.08, 112.78, 110.51, 104.94, 52.08, 42.36, 32.53, 11.94. HRMS (ESI) m/z calcd for $C_{31}H_{26}N_4O$ $[M-H]^+$ 471.2179, found 471.2180.

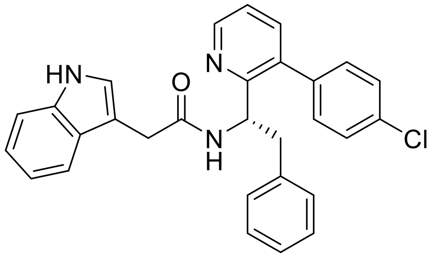

White solid, *(S)-N-(1-(3-(4-chlorophenyl)pyridin-2-yl)-2-phenylethyl)-2-(1H-indol-3-yl)acetamide* (**13**). Yield 88%. ^{1}H NMR (400 MHz, $CDCl_3$) δ 8.48 – 8.40 (m, 2H), 7.42 (dd, *J* = 16.3, 7.9 Hz, 2H), 7.34 – 7.24 (m, 3H), 7.24 – 7.09 (m, 2H), 7.03 (dt, *J* = 10.1, 3.4 Hz, 4H), 6.95 (t, *J* = 7.5 Hz, 2H), 6.87 (d, *J* = 8.0 Hz, 2H), 6.54 – 6.47 (m, 2H), 5.47 (q, *J* = 7.8 Hz, 1H), 3.68 (s, 2H), 2.89 – 2.81 (m, 2H). ^{13}C NMR (100 MHz, $CDCl_3$) δ 171.05, 156.81, 147.26, 138.90, 136.68, 136.58, 135.97, 134.23, 130.61, 129.32, 128.72, 128.28, 127.29, 126.50, 123.88, 122.59, 122.37, 119.83, 118.96, 111.39, 109.04, 51.86, 42.16, 33.67 (one carbon merging). HRMS (ESI) m/z calcd for $C_{29}H_{24}ClN_3O$ $[M + H]^-$ 464.1535, found 464.1535.

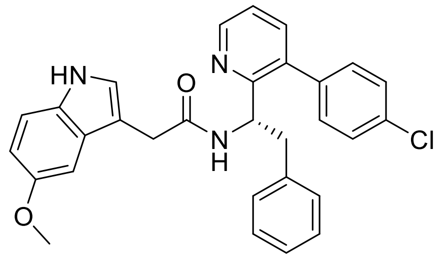

White solid, *(S)-N-(1-(3-(4-chlorophenyl)pyridin-2-yl)-2-phenylethyl)-2-(5-methoxy-1H-indol-3-yl)acetamide* (**14**). Yield 83%. ^{1}H NMR (600 MHz, $CDCl_3$) δ 8.47 (d, *J* = 4.9 Hz, 1H), 8.43 (s, 1H), 7.55 (s, 1H), 7.35 (s, 2H), 7.32 – 7.28 (m, 2H), 7.23 (dt, *J* = 8.7, 2.3 Hz, 1H), 7.14 (s, 1H), 7.06 (t, *J* = 7.4 Hz, 1H), 6.95 (t, *J* = 7.5 Hz, 4H), 6.89 (d, *J* = 2.3 Hz, 1H), 6.83 (dq, *J* = 8.7, 2.1 Hz, 1H), 6.52 (d, *J* = 7.5 Hz, 2H), 5.47 (q, *J* = 7.8 Hz, 1H), 3.75 (s, 3H), 3.69 (d, *J* = 4.9 Hz, 2H), 2.97 (d, *J* = 7.6 Hz, 2H). ^{13}C NMR (100 MHz, $CDCl_3$) δ 171.42, 156.54, 154.33, 136.29, 134.60, 131.59, 130.60, 129.19, 128.83, 128.35, 127.67, 126.63, 124.82, 123.05, 112.85, 112.18, 108.64, 100.39, 55.94, 51.90, 41.89, 33.70 (four carbons are too short to pick between 147 - 134 ppm). HRMS (ESI) m/z calcd for $C_{30}H_{26}ClN_3O_2$ $[M + H]^-$ 494.1641, found 494.1640.

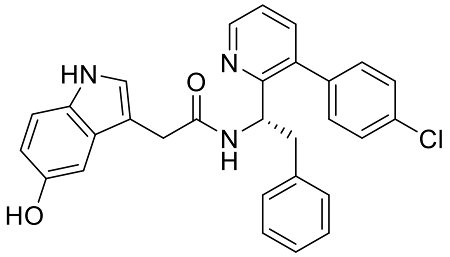

White solid, *(S)-N-(1-(3-(4-chlorophenyl)pyridin-2-yl)-2-phenylethyl)-2-(5-hydroxy-1H-indol-3-yl)acetamide* (**15**). Yield 70%. ^{1}H NMR (600 MHz, $CDCl_3$) δ 8.53 (d, *J* = 4.9 Hz, 1H), 8.43 (s, 1H), 7.44 (d, *J* = 7.7 Hz, 1H), 7.33 (s, 1H), 7.26 (dd, *J* = 16.3, 8.0 Hz, 3H), 7.15 (d, *J* = 8.6 Hz, 1H), 7.10 (t, *J* = 7.4 Hz, 1H), 7.00 (dd, *J* = 17.4, 9.9 Hz, 4H), 6.93 (d, *J* = 7.9 Hz, 2H), 6.82 (dd, *J* = 8.7, 2.3 Hz, 1H), 6.56 (d, *J* = 7.5 Hz, 2H), 5.51 (q, *J* = 7.7 Hz, 1H), 3.68 – 3.59 (m, 2H), 2.97 (d, *J* = 7.5 Hz, 2H). ^{13}C NMR (100 MHz, $CDCl_3$) δ 171.81, 156.58, 150.47, 147.10, 139.28, 136.84, 136.51, 135.79, 134.21, 131.50, 130.60, 129.29, 128.68, 128.32, 128.02, 126.53, 124.83, 122.84, 112.59, 112.11, 108.01, 103.43, 52.08, 41.90, 33.57. HRMS (ESI) m/z calcd for $C_{29}H_{24}ClN_3O_2$ $[M + H]^-$ 480.1484, found 480.1486.

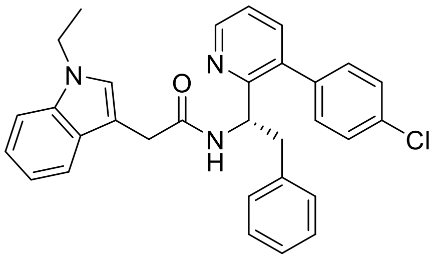

White solid, *(S)-N-(1-(3-(4-chlorophenyl)pyridin-2-yl)-2-phenylethyl)-2-(1-ethyl-1H-indol-3-yl)acetamide* (**16**). Yield 79%. ^{1}H NMR (400 MHz, $CDCl_3$) δ 8.32 (dd, *J* = 4.8, 1.7 Hz, 1H), 7.33 (dt, *J* = 8.0, 1.0 Hz, 1H), 7.30 – 7.24 (m, 1H), 7.23 – 7.02 (m, 6H), 6.92 (dddd, *J* = 8.0, 5.4, 2.8, 1.2 Hz, 3H), 6.88 – 6.81 (m, 2H), 6.76 (d, *J* = 8.0 Hz, 2H), 6.44 – 6.37 (m, 2H), 5.34 (td, *J* = 8.1, 6.7 Hz, 1H), 4.02 (q, *J* = 7.3 Hz, 2H), 3.60 – 3.53 (m, 2H), 2.74 (dd, *J* = 7.4, 2.3 Hz, 2H), 1.33 (t, *J* = 7.3 Hz, 3H). ^{13}C NMR (100 MHz, $CDCl_3$) δ 171.01, 156.88, 147.39, 138.68, 136.80, 136.51,

136.27, 136.08, 134.15, 130.61, 129.33, 128.70, 128.22, 127.93, 126.58, 126.43, 122.47, 121.84, 119.33, 119.21, 109.47, 107.72, 51.78, 42.15, 41.01, 33.62, 15.60. HRMS (ESI) m/z calcd for $C_{31}H_{28}ClN_3O$ [M + H]$^-$ 492.1848, found 492.1843.

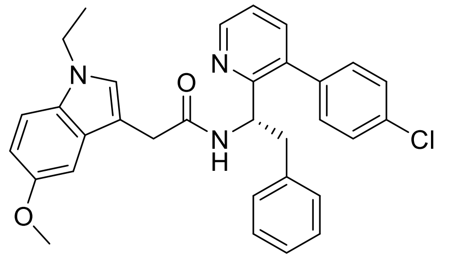

White solid, *(S)-N-(1-(3-(4-chlorophenyl)pyridin-2-yl)-2-phenylethyl)-2-(1-ethyl-5-methoxy-1H-indol-3-yl)acetamide* (**17**). Yield 68%. ^{1}H NMR (400 MHz, $CDCl_3$) δ 8.45 (dd, *J* = 4.7, 1.7 Hz, 1H), 7.42 (d, *J* = 7.4 Hz, 1H), 7.33 – 7.16 (m, 4H), 7.12 – 7.00 (m, 3H), 6.99 – 6.89 (m, 6H), 6.87 (dd, *J* = 5.6, 3.0 Hz, 1H), 6.56 – 6.49 (m, 2H), 5.47 (td, *J* = 8.2, 6.7 Hz, 1H), 4.13 (q, *J* = 7.3 Hz, 2H), 3.75 (s, 3H), 3.66 (s, 2H), 2.88 (d, *J* = 6.6 Hz, 2H), 1.46 (t, *J* = 7.3 Hz, 3H). ^{13}C NMR (101 MHz, $CDCl_3$) δ 171.19, 156.86, 154.12, 136.71, 134.20, 131.58, 130.62, 129.28, 128.71, 128.23, 128.18, 127.13, 126.45, 122.54, 112.10, 110.33, 107.15, 100.64, 55.96, 51.86, 42.11, 41.18, 33.67, 15.65 (four carbons are too short to pick between 148 - 135 ppm). HRMS (ESI) m/z calcd for $C_{32}H_{30}ClN_3O_2$ [M + H]$^-$ 522.1954, found 522.1954.

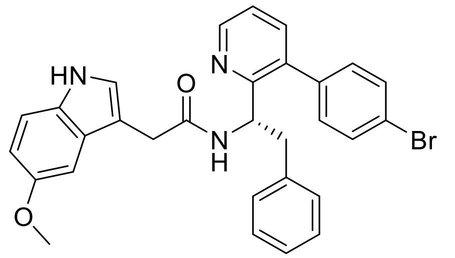

White solid, *(S)-N-(1-(3-(4-bromophenyl)pyridin-2-yl)-2-phenylethyl)-2-(5-methoxy-1H-indol-3-yl)acetamide* (**18**). Yield 40%. ^{1}H NMR (400 MHz, $CDCl_3$) δ 8.44 (dd, *J* = 5.0, 1.7 Hz, 1H), 8.19 (s, 1H), 7.47 (d, *J* = 7.5 Hz, 1H), 7.31 – 7.20 (m, 5H), 7.15 – 7.10 (m, 1H), 7.08 – 6.98 (m, 1H),

6.98 – 6.77 (m, 7H), 6.50 (dd, *J* = 8.0, 1.4 Hz, 2H), 5.45 (q, *J* = 7.8 Hz, 1H), 3.72 (s, 3H), 3.67 (s, 2H), 2.90 (d, *J* = 7.6 Hz, 2H). ^{13}C NMR (101 MHz, $CDCl_3$) δ 171.32, 156.65, 154.40, 137.28, 136.41, 134.53, 131.57, 130.63, 129.64, 129.24, 128.82, 128.34, 128.04, 127.71, 126.61, 124.72, 122.92, 112.96, 112.16, 108.84, 100.42, 55.94, 51.90, 41.95, 33.72 (one carbon merging).

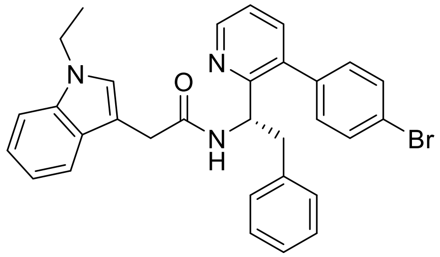

White solid, *(S)-N-(1-(3-(4-bromophenyl)pyridin-2-yl)-2-phenylethyl)-2-(1-ethyl-1H-indol-3-yl)acetamide* (**19**). Yield 35%. ^{1}H NMR (600 MHz, $CDCl_3$) δ 8.50 (s, 1H), 7.48 (d, *J* = 7.8 Hz, 1H), 7.32 (dd, *J* = 23.0, 8.2 Hz, 4H), 7.21 (t, *J* = 7.6 Hz, 1H), 7.11 (s, 1H), 7.07 (q, *J* = 7.5 Hz, 3H), 6.98 (t, *J* = 7.5 Hz, 2H), 6.93 (s, 2H), 6.55 (d, *J* = 7.5 Hz, 2H), 5.48 (q, *J* = 7.8 Hz, 1H), 4.17 (q, *J* = 7.3 Hz, 2H), 3.71 (s, 2H), 2.93 (s, 2H), 1.48 (t, *J* = 7.3 Hz, 3H). ^{13}C NMR (101 MHz, $CDCl_3$) δ 171.13, 156.85, 136.71, 136.28, 134.29, 130.63, 129.82, 129.33, 129.18, 128.75, 128.50, 128.27, 127.94, 127.17, 126.65, 126.49, 122.62, 121.83, 119.34, 119.23, 109.48, 107.69, 51.80, 42.09, 41.03, 33.62, 15.61.

Scheme S3. Synthesis of intermediates **39-41** and compounds **20-25**.

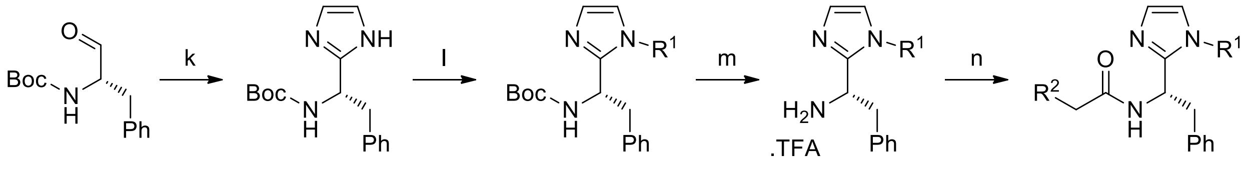

38 **39** **40** **41** **20-25**

Reagents and conditions: k) glyoxal, NH_3(g), MeOH, -78 °C - rt, 4 days; l) boronic acid, $Cu(OAc)_2$, DIPEA, DCM, 4Å MS, rt, 4 days; m) TFA, DCM, rt, 12 h; n) acid, HATU, DIPEA, DMF, rt

Synthesis of intermediate 39: To a solution of *N*-Boc-*L*-phenylalaninal **38** (1.0 gm, 1 equiv.) in MeOH (10 mL) in a microwave vial, glyoxal (1.0 equiv.) was added and the mixture was bubbled with ammonia gas at -50 ℃ for an hour. One the reaction turned turbid, the reaction mixture was warmed to room temperature slowly and stirred for 4 days. Upon completion, indicated by TLC, gas build-up was slowly removed by needle piercing. The reaction mixture was evaporated to dryness, diluted with water and extracted with EtOAc (3x50 mL). The combined organic phases were washed with water, brine, dried over anhydrous Na_2SO_4, filtered and concentrated. The crude product was purified by Combi-flash on silica gel using 60-100% EtOAc/hexane to get the desired cyclized intermediate **39** as white solid. Precaution needed to be taken during column as the product in UV-inactive.

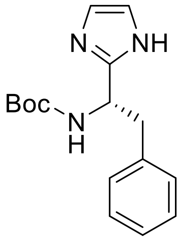

^{1}H NMR (400 MHz, $CDCl_3$) δ 7.28 – 7.15 (m, 3H), 7.13 – 7.05 (m, 2H), 6.91 (d, *J* = 1.0 Hz, 2H), 5.70 (s, 1H), 4.94 (qd, *J* = 7.8, 2.1 Hz, 1H), 3.37 – 3.25 (m, 2H), 1.37 (s, 9H). ^{13}C NMR (100 MHz, $CDCl_3$) δ 156.34, 148.60, 137.38, 129.37, 128.67, 128.63, 126.84, 80.27, 50.60, 39.98, 28.44.

Synthesis of intermediate 40: To a solution of cyclized intermediate **39** (0.1 gm, 1 equiv.) in dry DCM (10 mL), corresponding boronic acid (2.0 equiv.), $Cu(OAc)_2$ (1.0 equiv.), DIPEA (3.0 equiv.) and 4Å MS (0.1 gm) were added and the mixture was stirred at room temperature under atmospheric oxygen for 4 days. Upon completion, indicated by TLC, the reaction mixture was filtered through short celite bed and evaporated to dryness. The crude product was purified by

Combi-flash on silica gel using 40-50% EtOAc/hexane to get the desired intermediate **40** as white solid.

Synthesis of intermediate 41: To a solution of intermediate **40** (0.1 gm, 1 equiv.) in DCM (10 mL), trifluoroacetic acid (10.0 equiv.) was added and the mixture was stirred at room temperature for 12 hours. Upon completion, indicated by TLC, the reaction mixture evaporated to dryness and used in for next step without further purification.

Synthesis of compounds 20-25: To a solution of amine trifluoroacetic acid salt **41** (0.1 gm, 1 equiv.) in DMF (1 mL) HATU (1.2 equiv.), DIPEA (2.0 equiv.) and desired acids (1.1 equiv.) were added and the mixture was stirred at room temperature for 12 hours. Upon completion, H_2O (10 mL) was added and the reaction mixture was extracted with EtOAc (3x10 mL). The combined organic phases were washed with water, brine, dried over anhydrous Na_2SO_4, filtered and concentrated. The crude product was purified by Combi-flash on silica gel using 80-100% EtOAc/hexane.

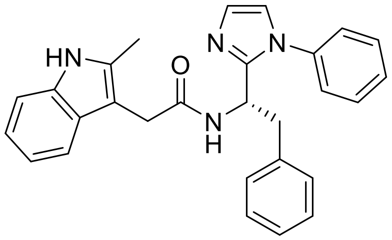

White solid, *(S)-2-(2-methyl-1H-indol-3-yl)-N-(2-phenyl-1-(1-phenyl-1H-imidazol-2-yl)ethyl)acetamide* (**20**). Yield 43%. ^{1}H NMR (600 MHz, $CDCl_3$) δ 8.75 (s, 1H), 7.40 (d, J = 7.8 Hz, 1H), 7.35 (tt, J = 7.8, 2.4 Hz, 1H), 7.30 (dd, J = 8.4, 6.9 Hz, 2H), 7.20 (d, J = 7.8 Hz, 1H), 7.15 – 6.99 (m, 8H), 6.85 – 6.80 (m, 3H), 6.71 – 6.67 (m, 2H), 5.29 (td, J = 9.0, 5.8 Hz, 1H), 3.64 (s, 2H), 3.04 (dd, J = 12.9, 9.6 Hz, 1H), 2.98 (dd, J = 12.9, 5.8 Hz, 1H), 2.20 (s, 3H). ^{13}C NMR (101 MHz, $CDCl_3$) δ 171.24, 147.79, 136.43, 136.19, 135.58, 133.58, 129.5, 129.4, 129.0, 128.9, 128.5,

128.4, 126.75, 126.19, 121.45, 121.00, 119.70, 117.93, 110.60, 104.52, 47.25, 41.94, 32.36, 11.79. HRMS (ESI) m/z calcd for $C_{28}H_{26}N_4O$ $[M + H]^+$ 435.2179, found 435.2182.

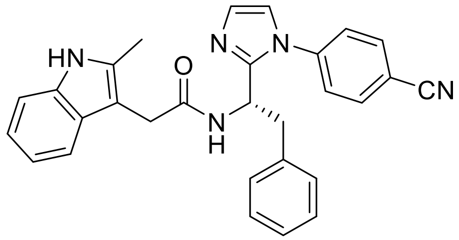

White solid, *(S)-N-(1-(1-(4-cyanophenyl)-1H-imidazol-2-yl)-2-phenylethyl)-2-(2-methyl-1H-indol-3-yl)acetamide* (**21**). Yield 51%. ^{1}H NMR (600 MHz, $CDCl_3$) δ 8.44 (s, 1H), 7.55 (d, J = 8.4 Hz, 2H), 7.40 (d, J = 7.8 Hz, 1H), 7.26 (t, J = 4.0 Hz, 1H), 7.15 – 7.01 (m, 6H), 6.94 (d, J = 8.3 Hz, 2H), 6.78 – 6.73 (m, 2H), 6.67 – 6.62 (m, 2H), 5.16 (ddd, J = 9.7, 8.4, 6.0 Hz, 1H), 3.65 (d, J = 3.9 Hz, 2H), 3.01 – 2.91 (m, 2H), 2.31 (s, 3H). ^{13}C NMR (100 MHz, $CDCl_3$) δ 171.35, 147.77, 140.18, 136.37, 135.57, 133.6, 133.5, 133.30, 129.26, 128.5, 128.4, 128.36, 126.93, 121.67, 120.25, 119.88, 117.9, 117.8, 112.51, 110.68, 104.31, 47.50, 42.23, 32.32, 11.80. HRMS (ESI) m/z calcd for $C_{29}H_{25}N_5O$ $[M + H]^+$ 460.2132, found 460.2133.

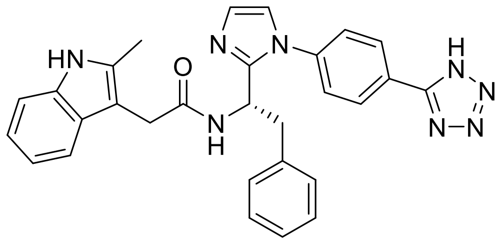

Brown solid, *(S)-N-(1-(1-(4-(1H-tetrazol-5-yl)phenyl)-1H-imidazol-2-yl)-2-phenylethyl)-2-(2-methyl-1H-indol-3-yl)acetamide* (**22**). Yield 65%. ^{1}H NMR (400 MHz, MeOD) δ 7.94 (dd, *J* = 8.6, 3.7 Hz, 2H), 7.29 (d, *J* = 7.6 Hz, 1H), 7.25 – 6.84 (m, 11H), 6.81 – 6.75 (m, 2H), 5.13 (t, *J* = 7.8 Hz, 1H), 4.92 – 4.86 (m, 2H), 3.64 – 3.51 (m, 2H), 3.16 – 3.01 (m, 2H), 2.29 (d, *J* = 3.1 Hz, 3H). ^{13}C NMR (100 MHz, MeOD) δ 173.64, 148.35, 138.34, 136.94, 136.44, 134.09, 129.61, 129.05,

128.99, 128.38, 127.52, 127.43 126.26, 122.25, 121.05, 119.22, 117.81, 110.80, 104.02, 48.47, 40.86, 31.71, 10.81. HRMS (ESI) m/z calcd for $C_{29}H_{26}N_8O$ $[M + H]^+$ 503.2302, found 503.2298.

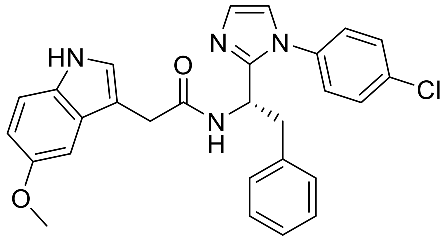

Brown solid, *(S)-N-(1-(1-(4-chlorophenyl)-1H-imidazol-2-yl)-2-phenylethyl)-2-(5-methoxy-1H-indol-3-yl)acetamide* (**23**). Yield 46%. ^{1}H NMR (400 MHz, $CDCl_3$) δ 8.81 (s, 1H), 7.47 – 7.39 (m, 1H), 7.31 – 7.19 (m, 3H), 7.18 – 7.11 (m, 2H), 7.08 – 7.00 (m, 3H), 6.97 (d, *J* = 2.4 Hz, 1H), 6.83 (dt, *J* = 8.8, 1.7 Hz, 1H), 6.82 – 6.68 (m, 3H), 6.70 – 6.61 (m, 2H), 5.16 (ddd, *J* = 10.4, 8.2, 5.4 Hz, 1H), 3.81 (s, 3H), 3.70 (d, *J* = 2.0 Hz, 2H), 3.14 (ddd, *J* = 13.5, 10.5, 3.0 Hz, 1H), 2.99 (ddd, *J* = 12.8, 5.6, 2.3 Hz, 1H). ^{13}C NMR (101 MHz, $CDCl_3$) δ 171.80, 154.37, 148.30, 136.01, 135.47, 134.08, 131.69, 129.68, 129.29, 128.64, 127.66, 127.56, 127.02, 125.35, 124.93, 120.99, 112.77, 112.30, 108.18, 100.41, 56.05, 47.53, 41.65, 33.49. HRMS (ESI) m/z calcd for $C_{28}H_{25}ClN_4O_2$ $[M + H]^-$ 483.1593, found 483.1598.

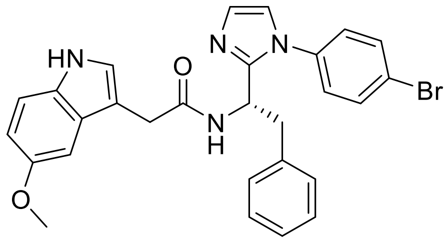

Brown solid, *(S)-N-(1-(1-(4-bromophenyl)-1H-imidazol-2-yl)-2-phenylethyl)-2-(5-methoxy-1H-indol-3-yl)acetamide* (**24**). Yield 42%. ^{1}H NMR (600 MHz, $CDCl_3$) δ 8.50 (d, *J* = 8.1 Hz, 1H), 8.25 (s, 1H), 7.48 – 7.43 (m, 3H), 7.38 (s, 1H), 7.24 (s, 1H), 7.18 (t, *J* = 7.5 Hz, 1H), 7.10 – 7.04 (m, 3H), 6.83 (dd, *J* = 8.9, 2.3 Hz, 2H), 6.73 (s, 2H), 6.68 (d, *J* = 7.5 Hz, 2H), 5.20 – 5.13 (m, 1H),

3.85 (s, 3H), 3.82 (s, 1H), 3.74 (d, J = 15.8 Hz, 1H), 3.58 (t, J = 12.4 Hz, 1H), 3.12 (dd, J = 13.1, 5.5 Hz, 1H). ^{13}C NMR (100 MHz, $CDCl_3$) δ 173.04, 154.37, 149.03, 134.95, 133.16, 132.49, 131.53, 129.18, 129.11, 127.79, 127.63, 127.59, 125.53, 125.28, 121.53, 119.60, 112.60, 112.22, 108.14, 100.68, 56.24, 47.89, 40.50, 33.38. HRMS (ESI) m/z calcd for $C_{28}H_{25}BrN_4O_2$ $[M + H]^-$ 527.1088, found 527.1092.

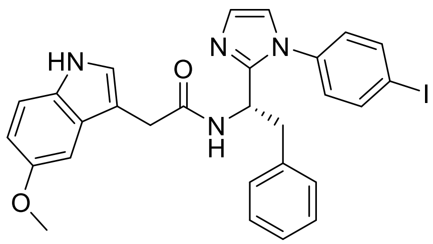

Brown solid, *(S)-N-(1-(1-(4-iodophenyl)-1H-imidazol-2-yl)-2-phenylethyl)-2-(5-methoxy-1H-indol-3-yl)acetamide* (**25**). Yield 52%.^{1}H NMR (400 MHz, $CDCl_3$) δ 8.47 (s, 1H), 7.92 (d, J = 8.3 Hz, 1H), 7.63 (d, J = 8.7 Hz, 2H), 7.27 – 7.20 (m, 3H), 7.16 – 7.05 (m, 1H), 7.09 – 6.98 (m, 3H), 6.83 (dd, J = 8.8, 2.4 Hz, 1H), 6.73 (dd, J = 8.8, 1.3 Hz, 1H), 6.70 – 6.63 (m, 2H), 6.55 (d, J = 8.1 Hz, 2H), 5.17 (ddd, J = 11.0, 8.2, 5.6 Hz, 1H), 3.83 (s, 3H), 3.77 – 3.64 (m, 2H), 3.33 (t, J = 12.0 Hz, 1H), 3.09 – 2.94 (m, 1H). ^{13}C NMR (100 MHz, $CDCl_3$) δ 172.33, 154.38, 148.55, 138.90, 135.53, 134.34, 131.60, 129.24, 128.84, 127.82, 127.73, 127.28, 125.17, 122.71, 121.12, 112.72, 112.25, 108.24, 100.54, 95.91, 56.14, 47.66, 41.12, 33.45. HRMS (ESI) m/z calcd for $C_{28}H_{25}IN_4O_2$ $[M + H]^-$ 575.0949, found 575.0945.

NMR Spectra of compounds **1-25**.

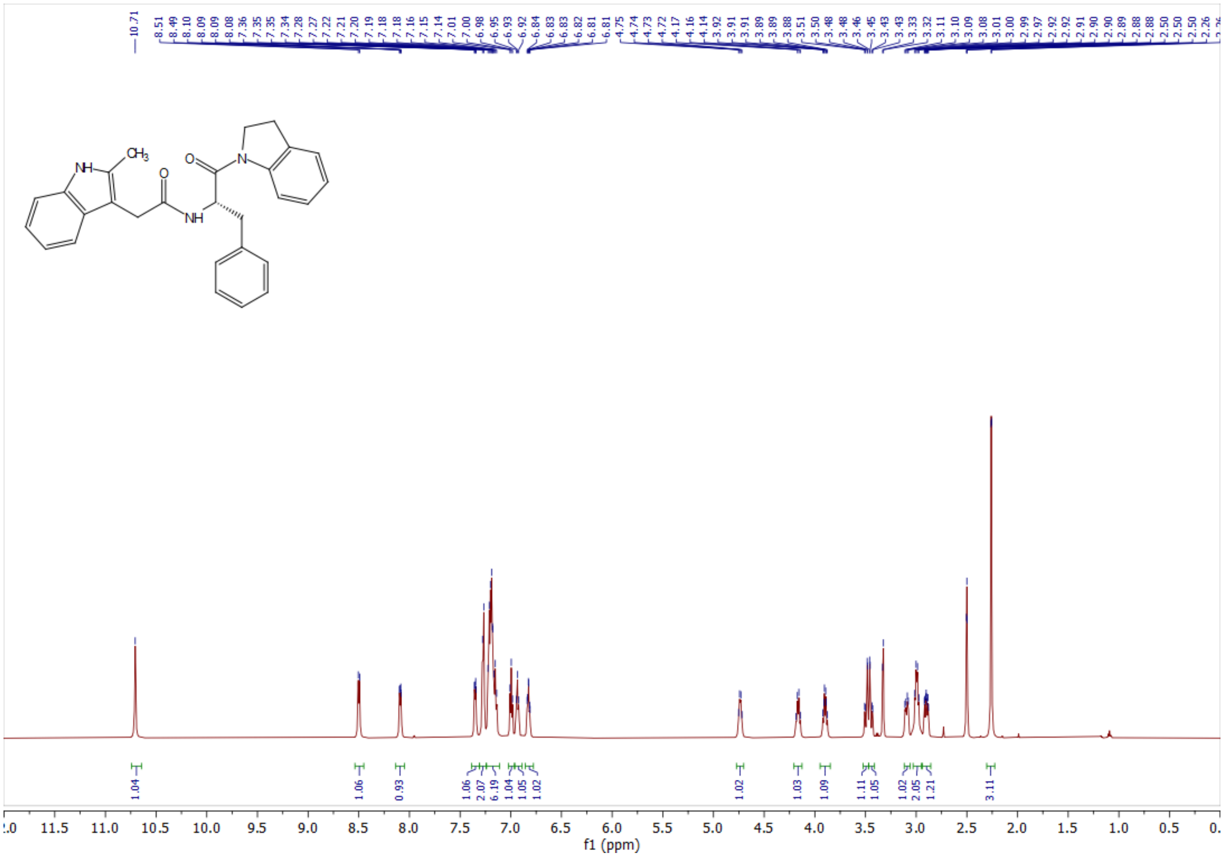

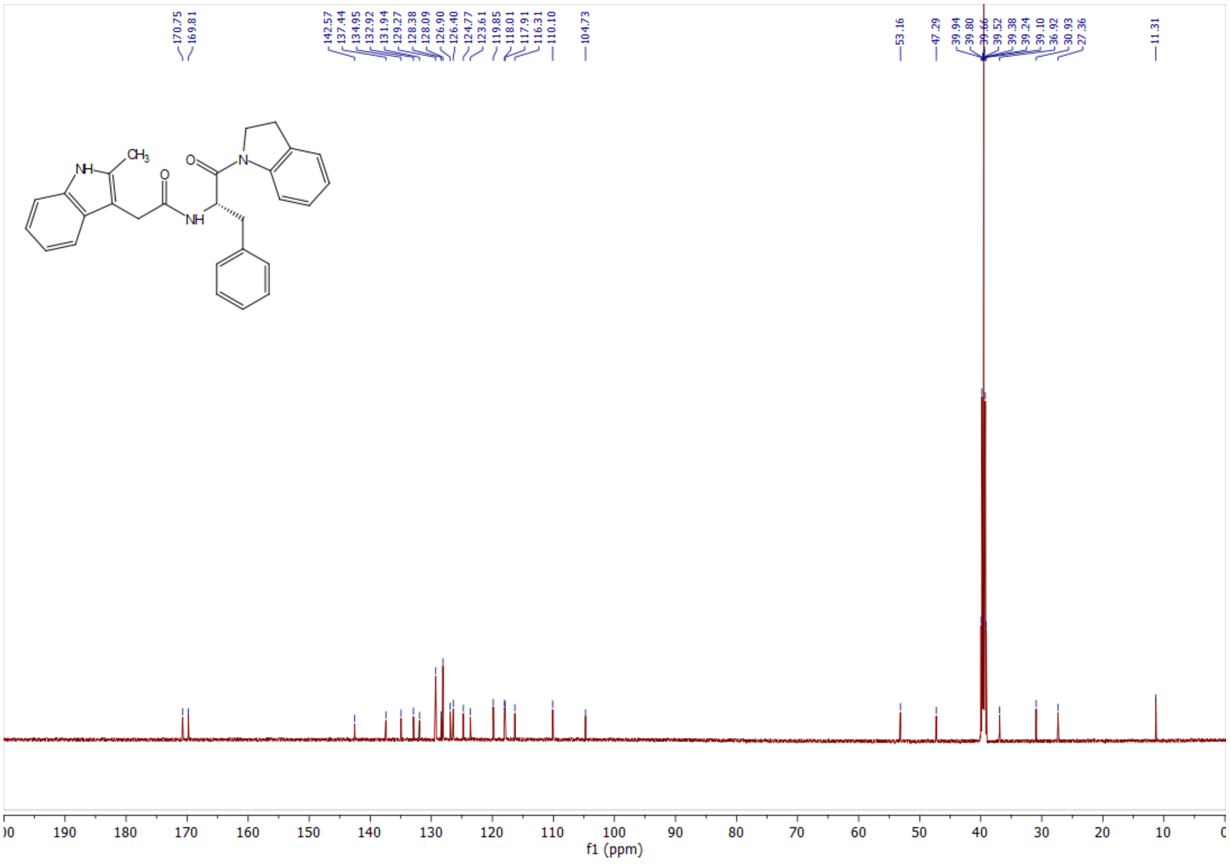

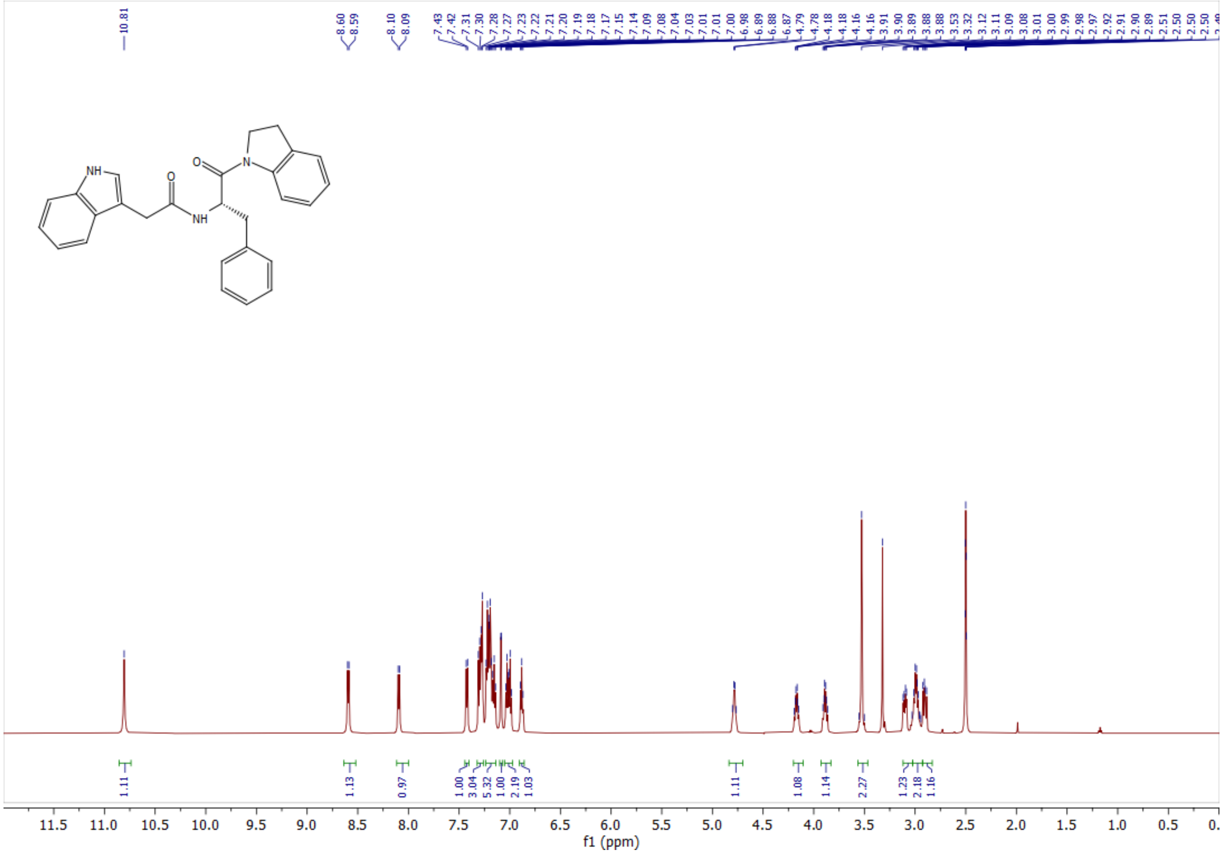

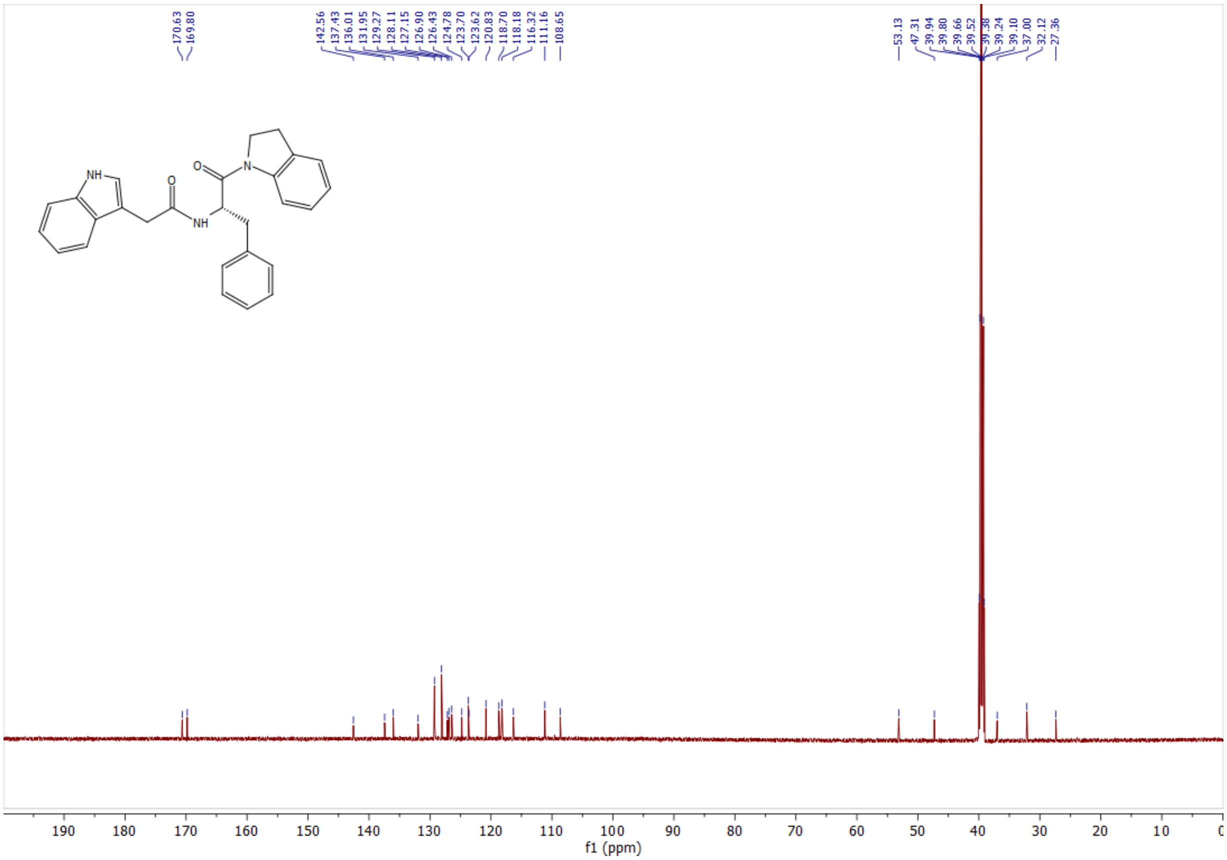

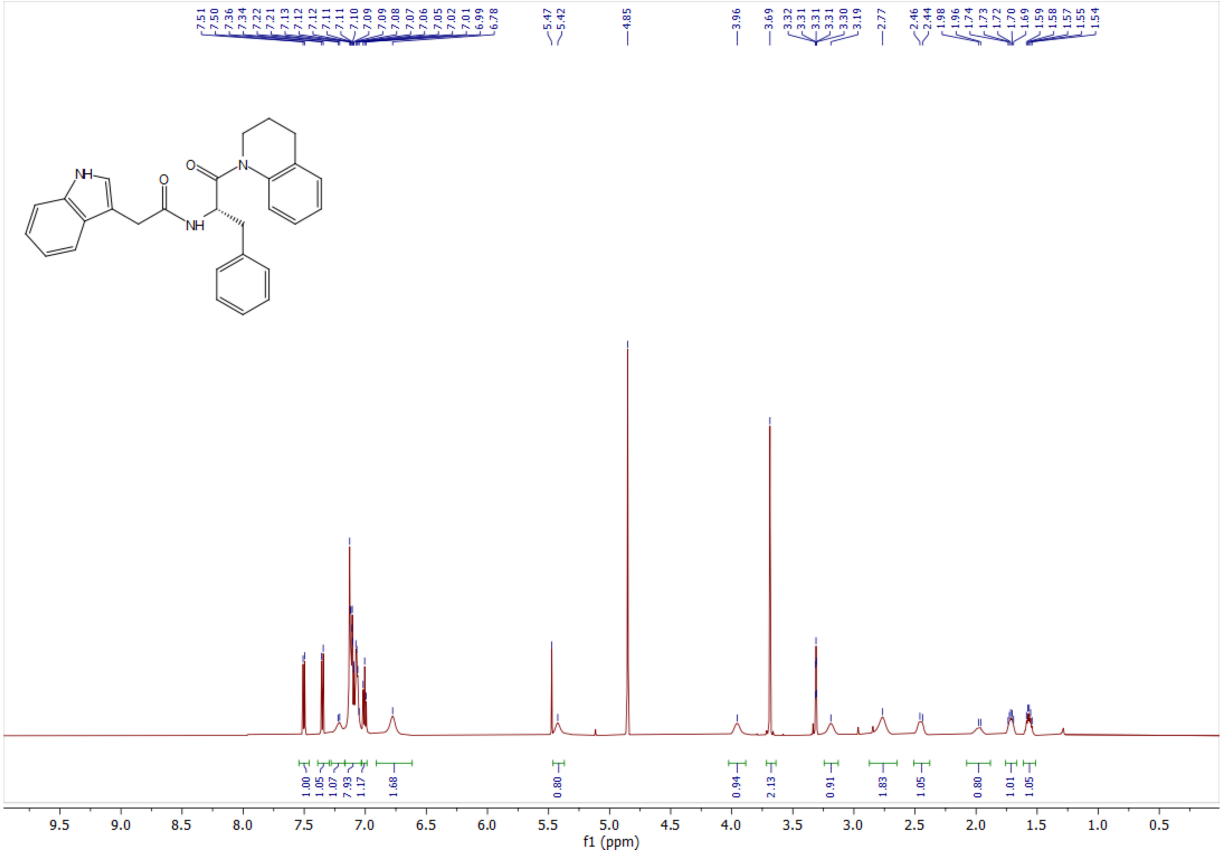

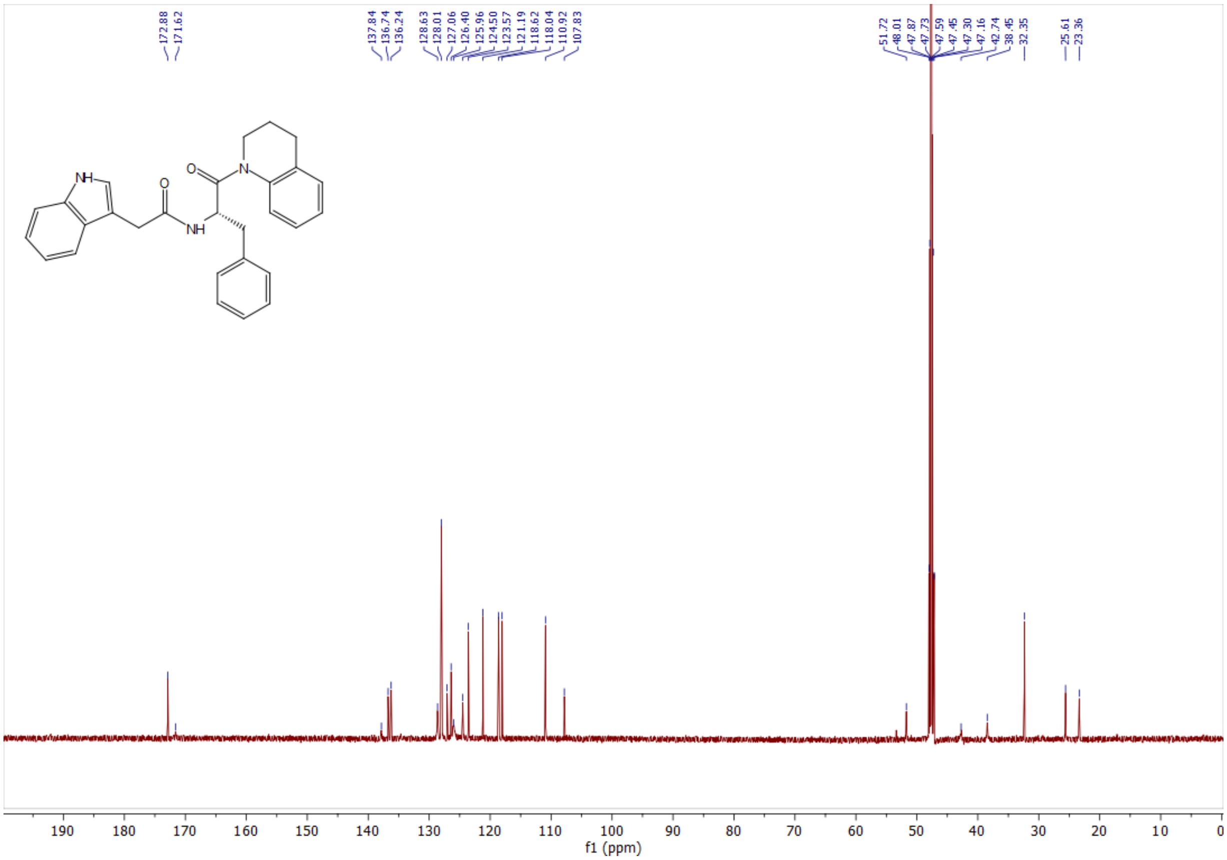

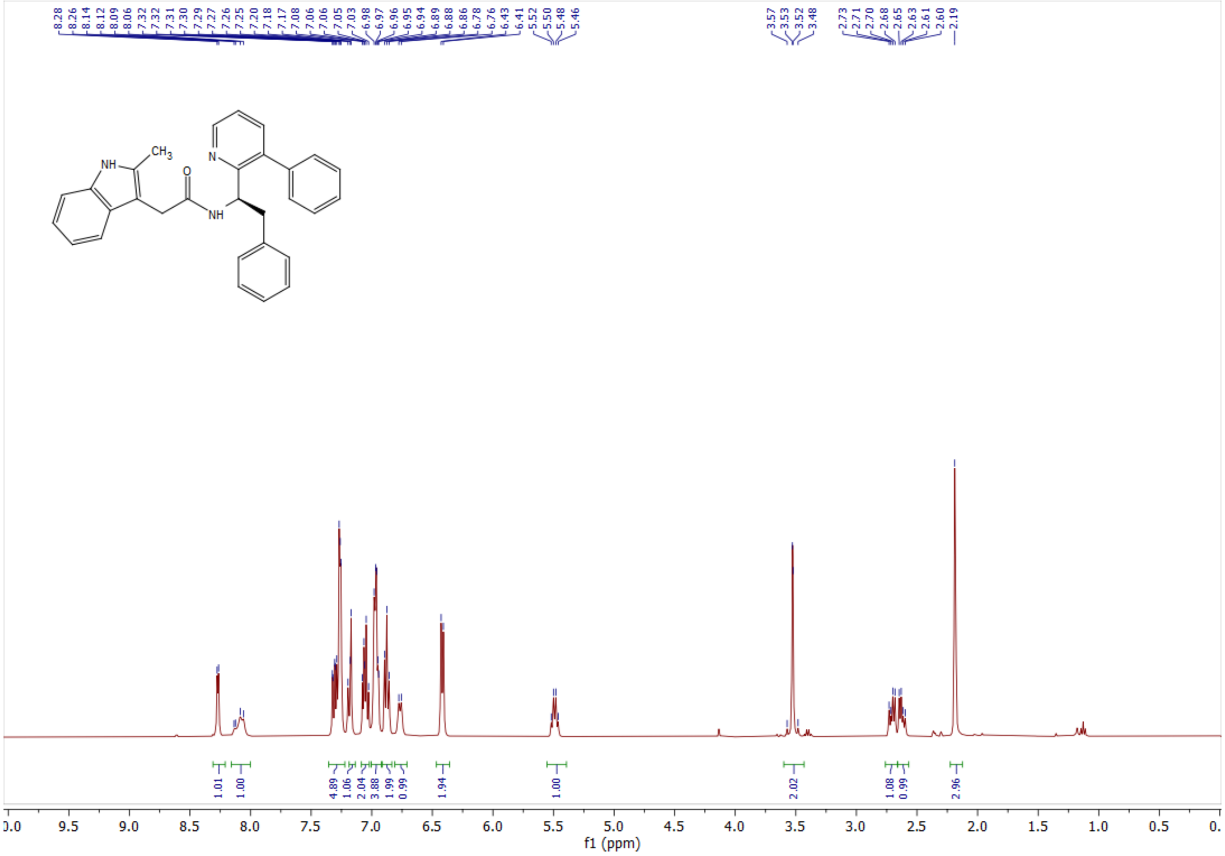

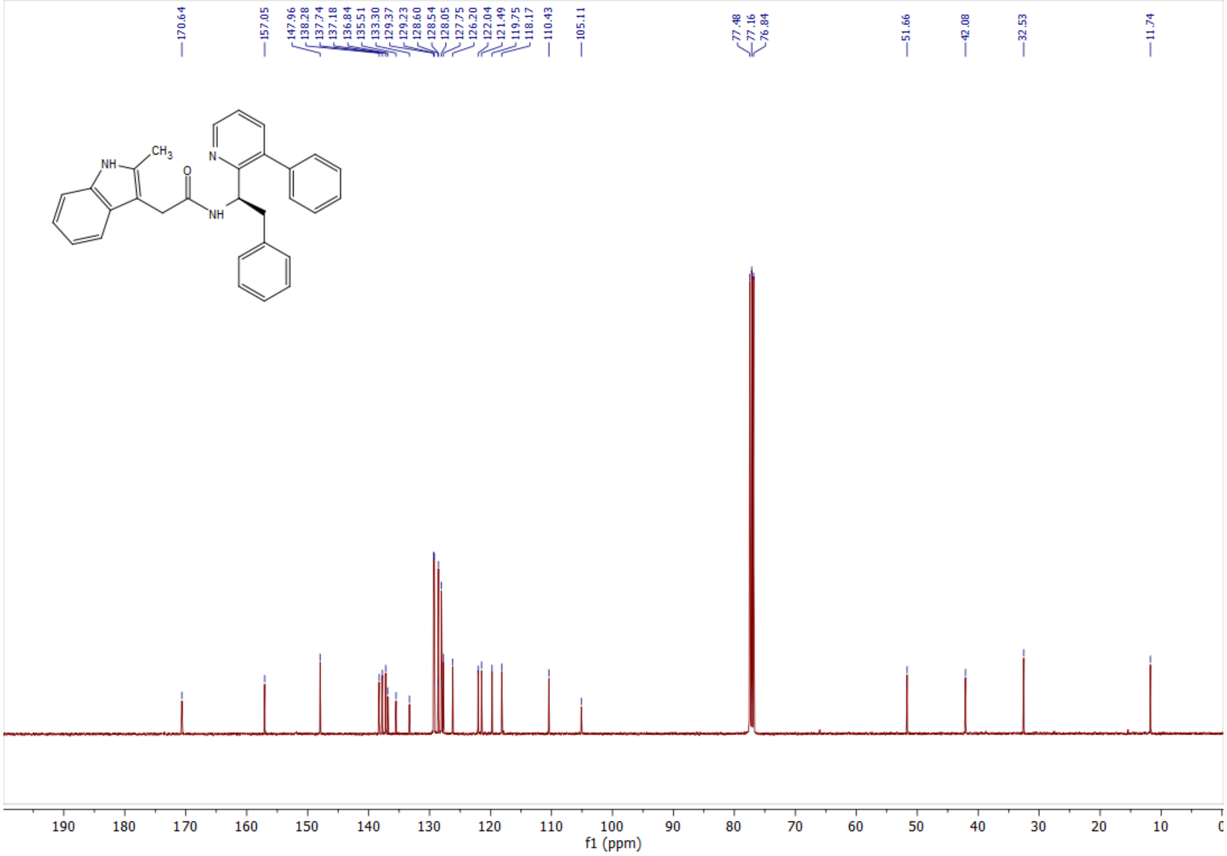

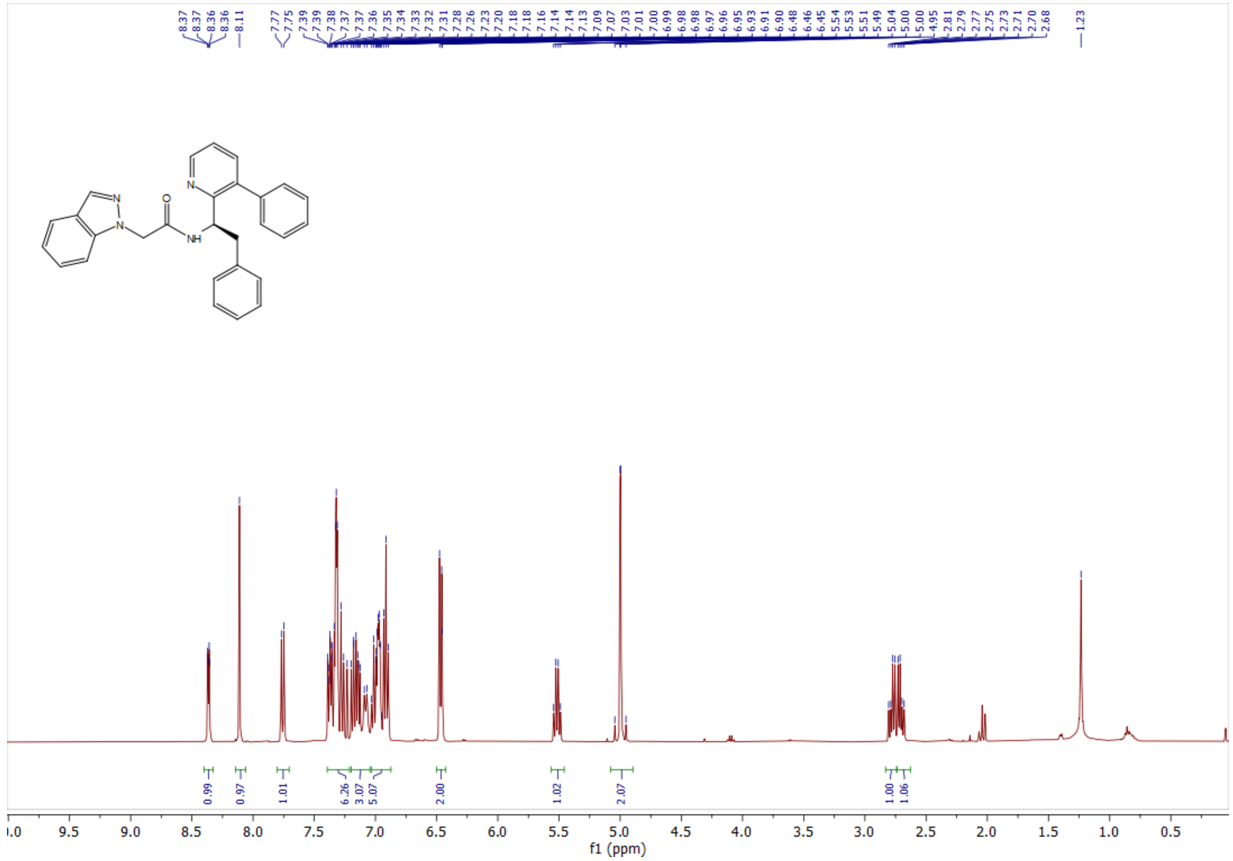

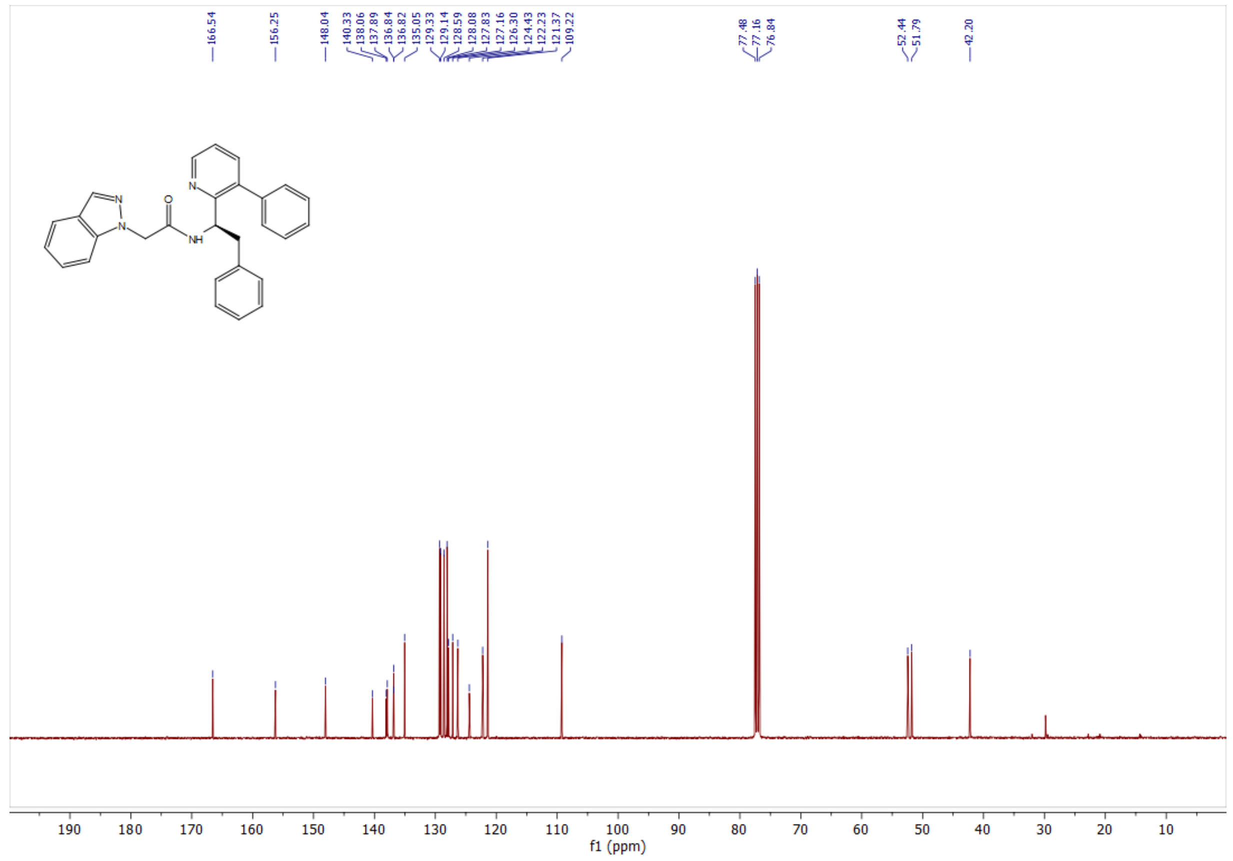

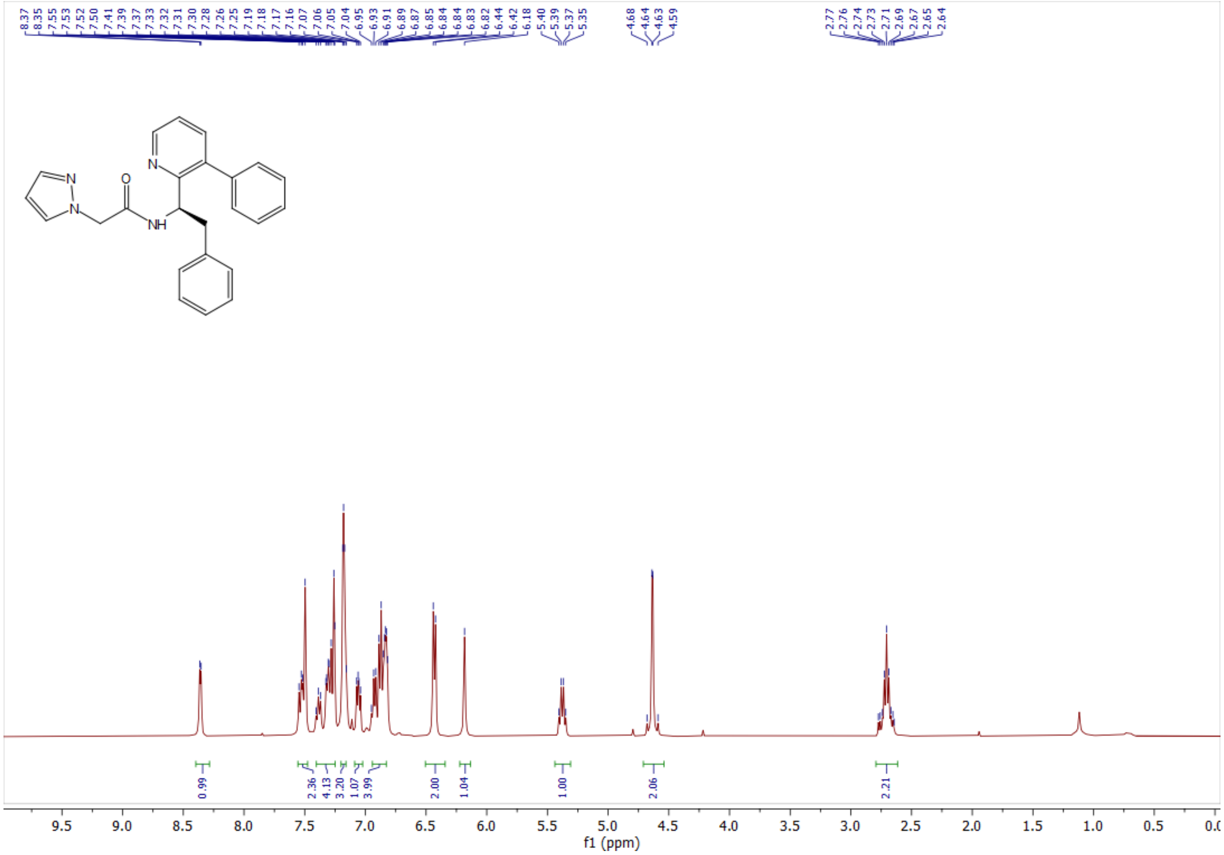

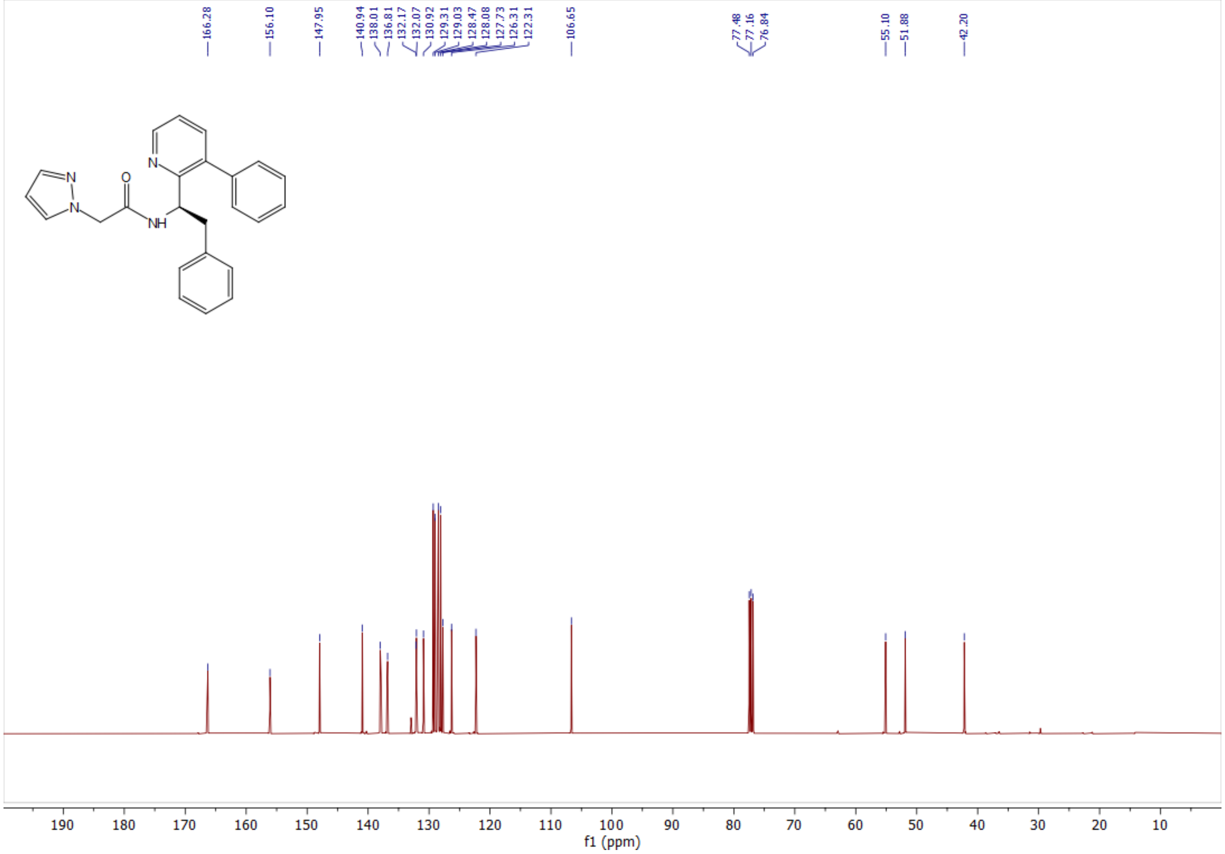

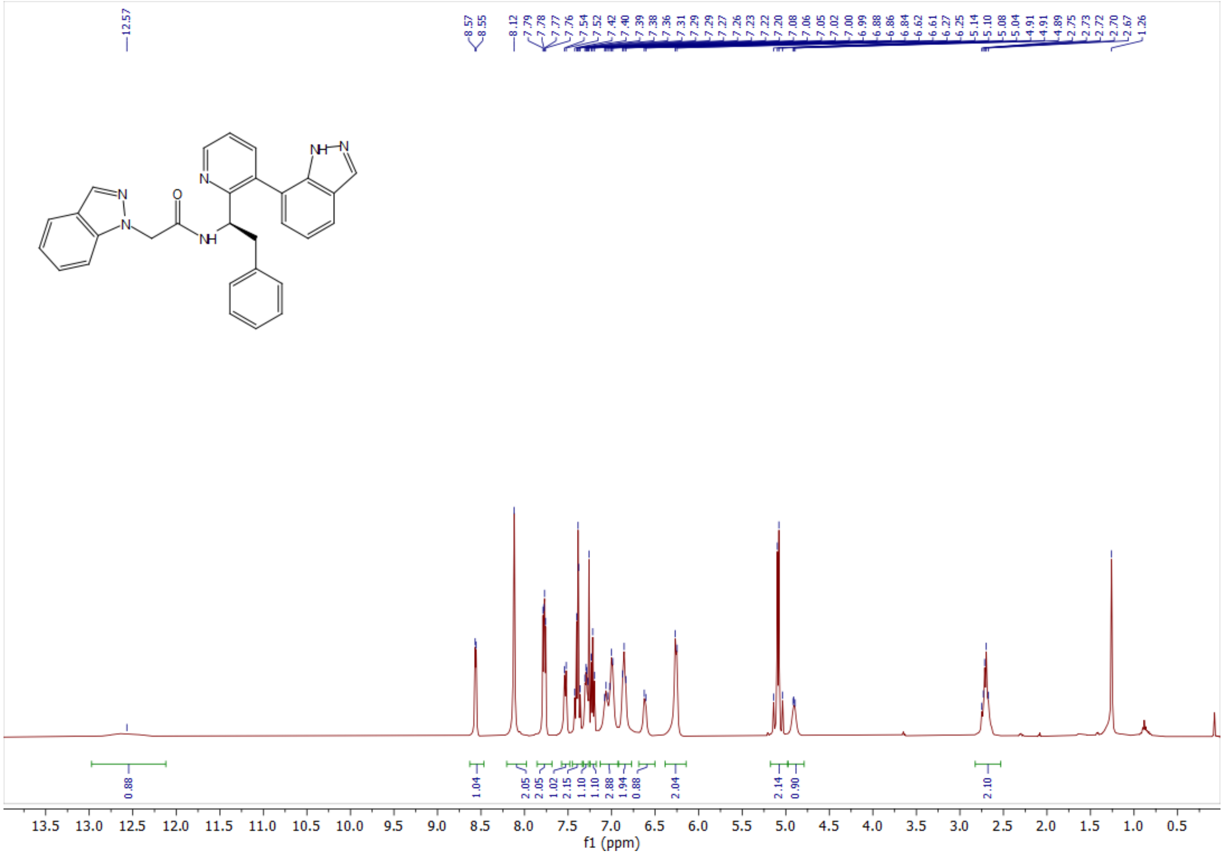
12.57
8.57
8.55
8.12
1.26
0.88
1.04
2.05
2.05
1.02
2.15
1.10
1.10
2.88
1.94
0.88
2.04
2.14
0.90
2.10
f1 (ppm)

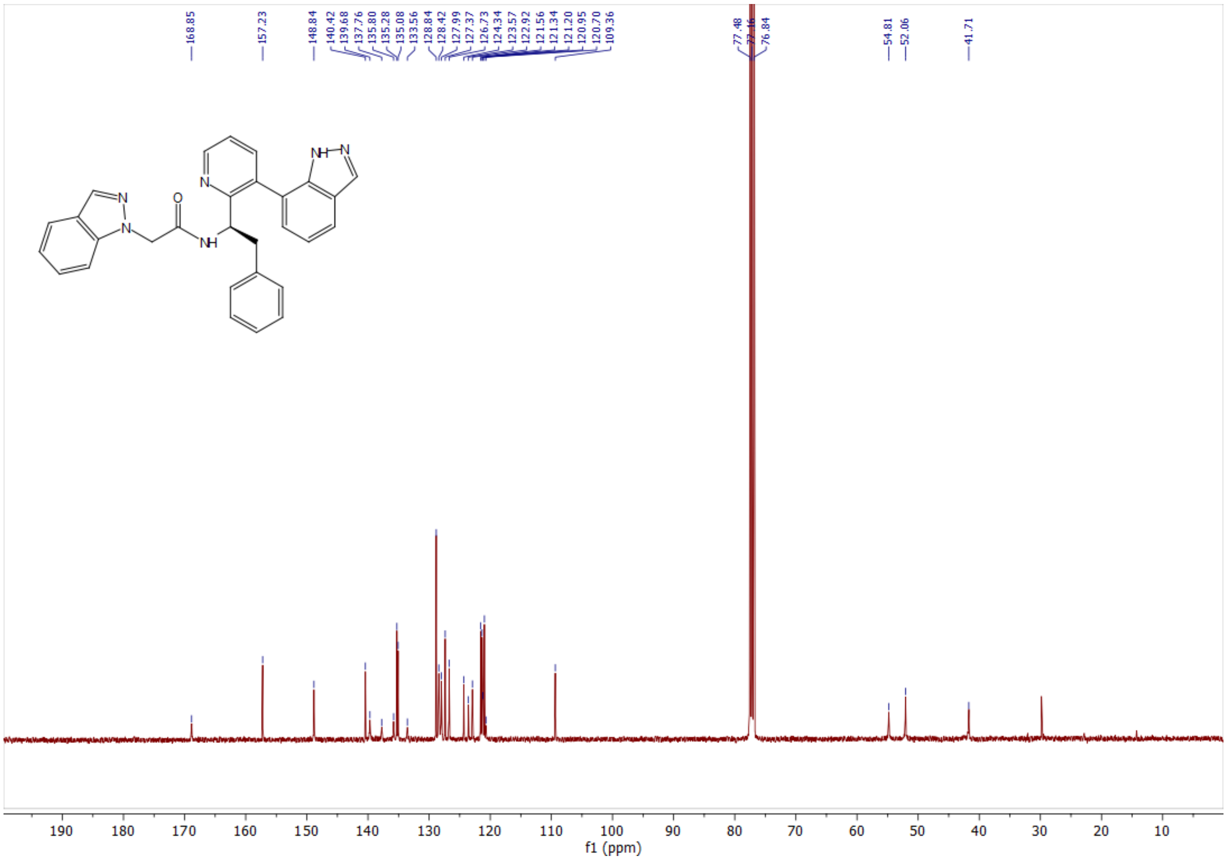
168.85
157.23
148.84
140.42
139.68
137.76
135.80
135.28
135.08
133.56
128.84
128.42
127.99
127.37
126.73
124.34
123.57
122.92
121.56
121.34
121.20
120.95
120.70
109.36
77.48
76.84
54.81
52.06
41.71
f1 (ppm)

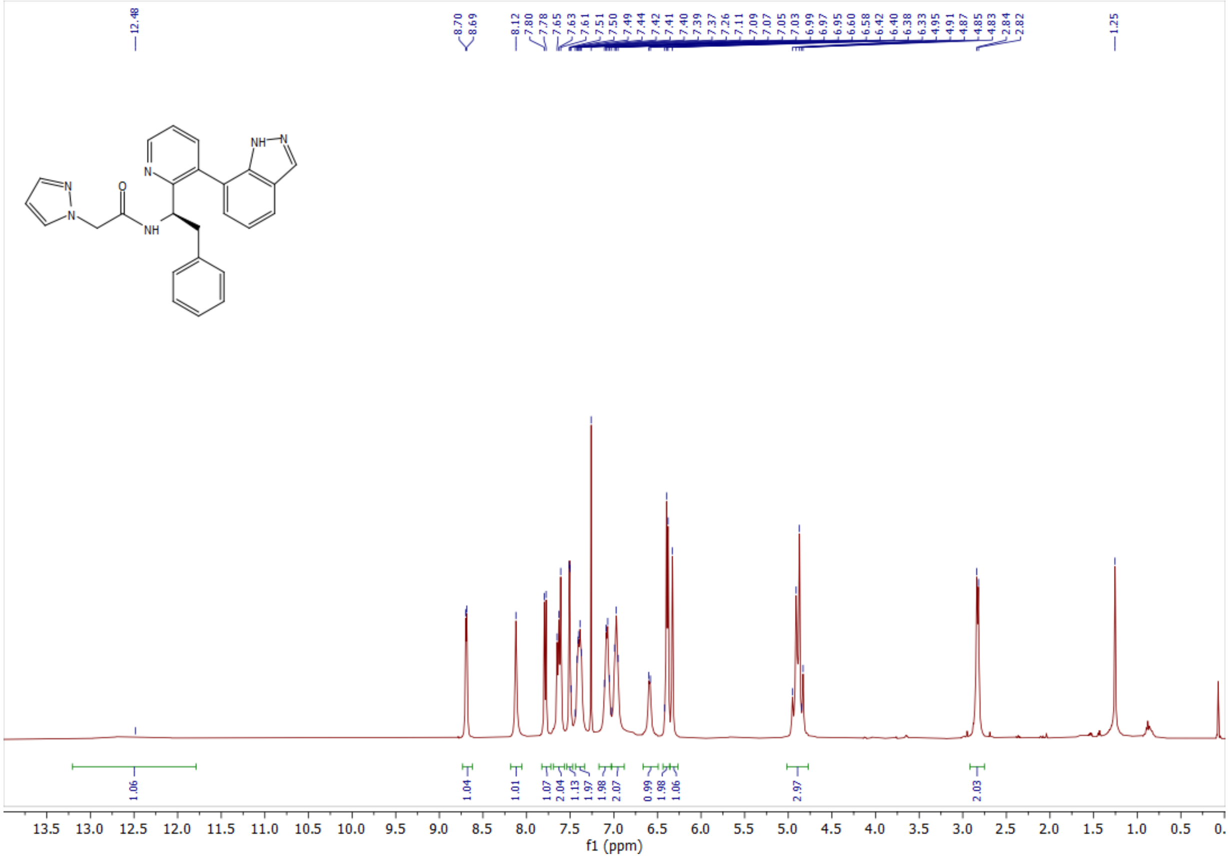
12.48
8.70
8.69
8.12
1.25
1.06
1.04
1.01
2.97
2.03
f1 (ppm)

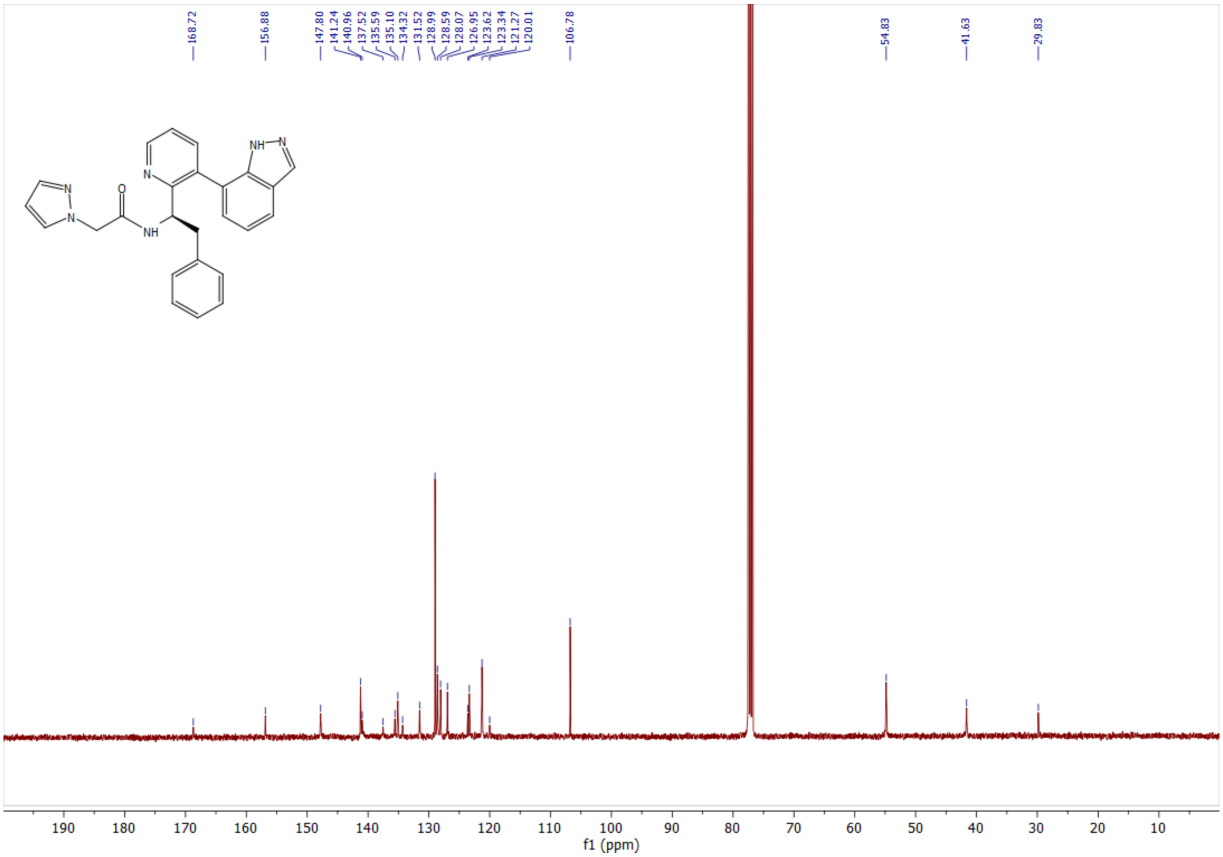
168.72
156.88
147.80
141.24
140.96
137.52
135.59
135.10
134.32
131.52
128.99
128.59
128.07
126.95
123.62
123.34
121.27
120.01
106.78
54.83
41.63
29.83
f1 (ppm)

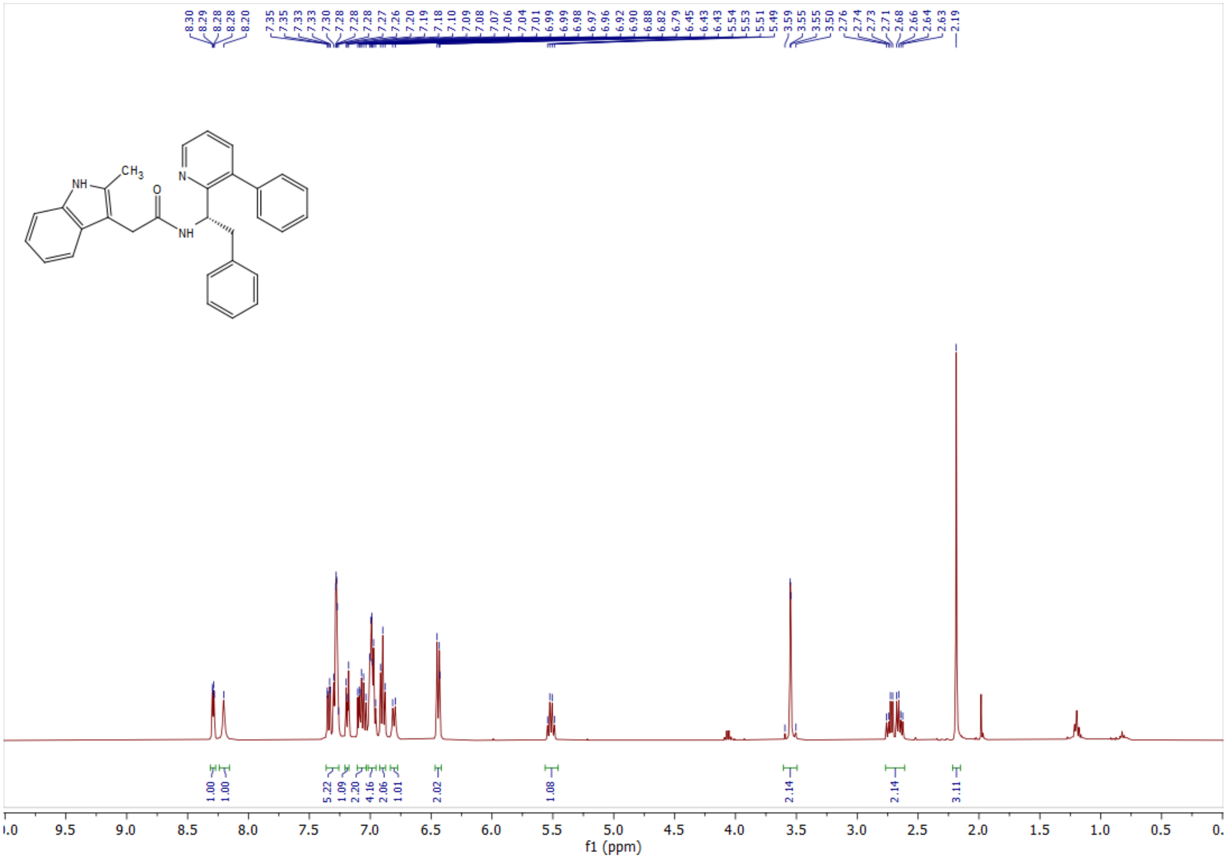

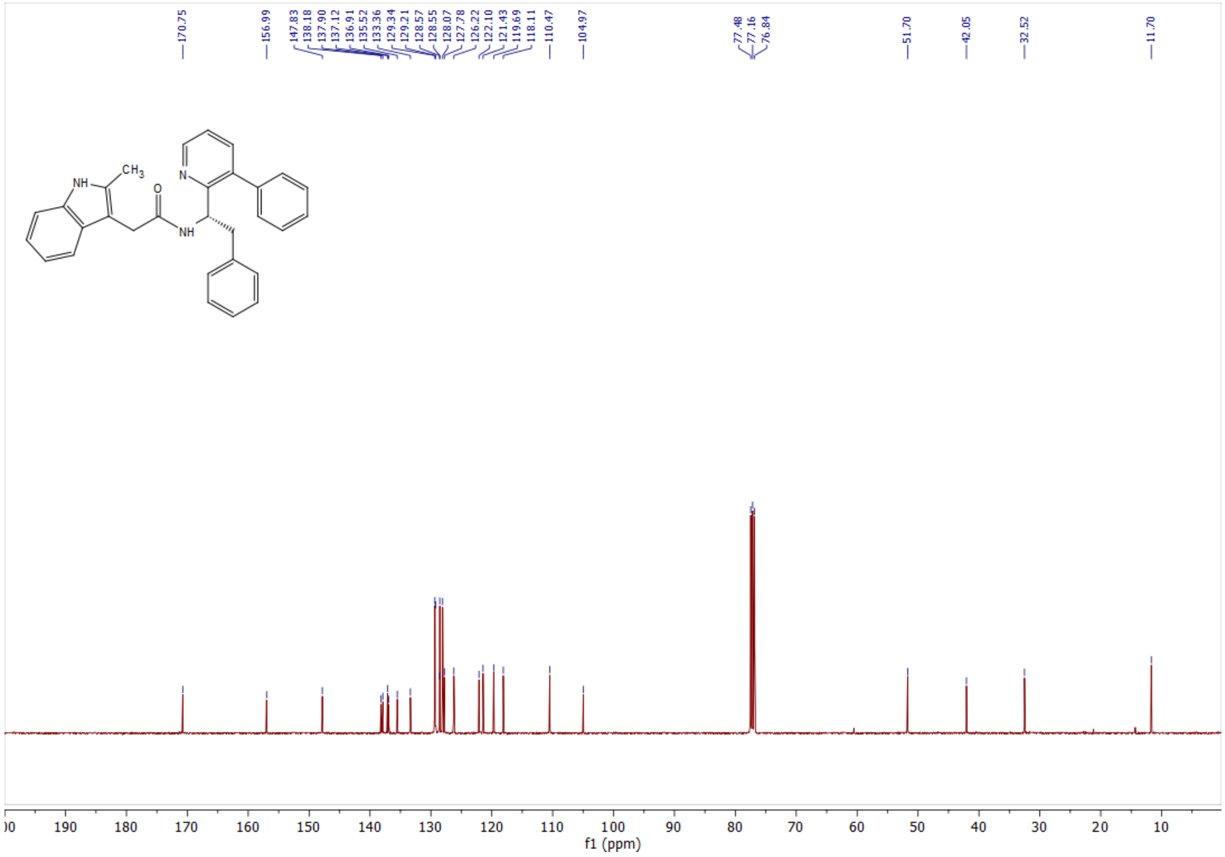

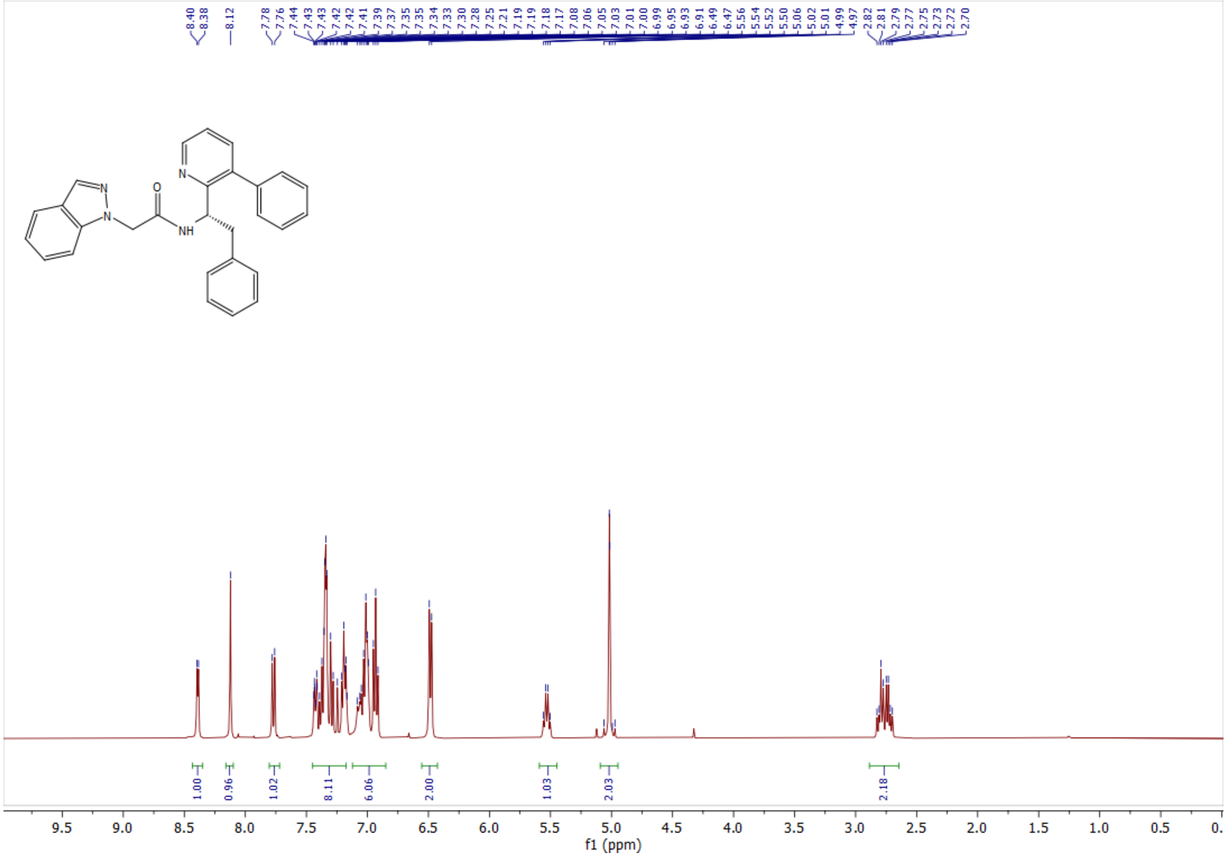
8.40
8.38
8.12
7.78
7.76
7.44
7.43
7.43
7.42
7.42
7.41
7.39
7.37
7.35
7.35
7.34
7.33
7.30
7.28
7.25
7.21
7.19
7.19
7.18
7.17
7.08
7.06
7.05
7.03
7.01
7.00
6.99
6.95
6.93
6.91
6.49
6.47
5.56
5.54
5.52
5.50
5.06
5.02
5.01
4.99
4.97
2.82
2.81
2.79
2.77
2.75
2.73
2.72
2.70
N
N
O
N
NH
1.00
0.96
1.02
8.11
6.06
2.00
1.03
2.03
2.18
f1 (ppm)

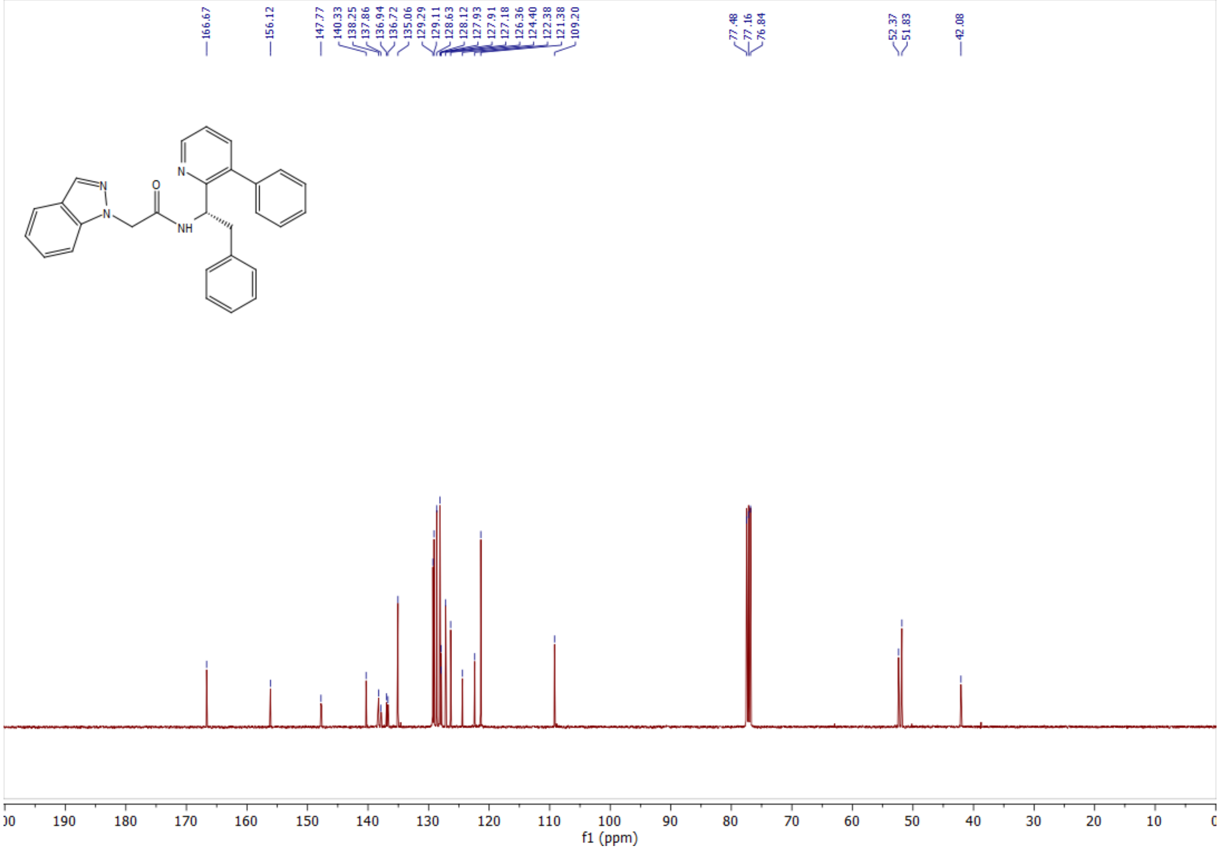
166.67
156.12
147.77
140.33
138.25
137.86
136.94
136.72
135.06
129.29
129.11
128.63
128.12
127.93
127.91
127.18
126.36
124.40
122.38
121.38
109.20
77.48
77.16
76.84
52.37
51.83
42.08
N
N
O
N
NH
f1 (ppm)

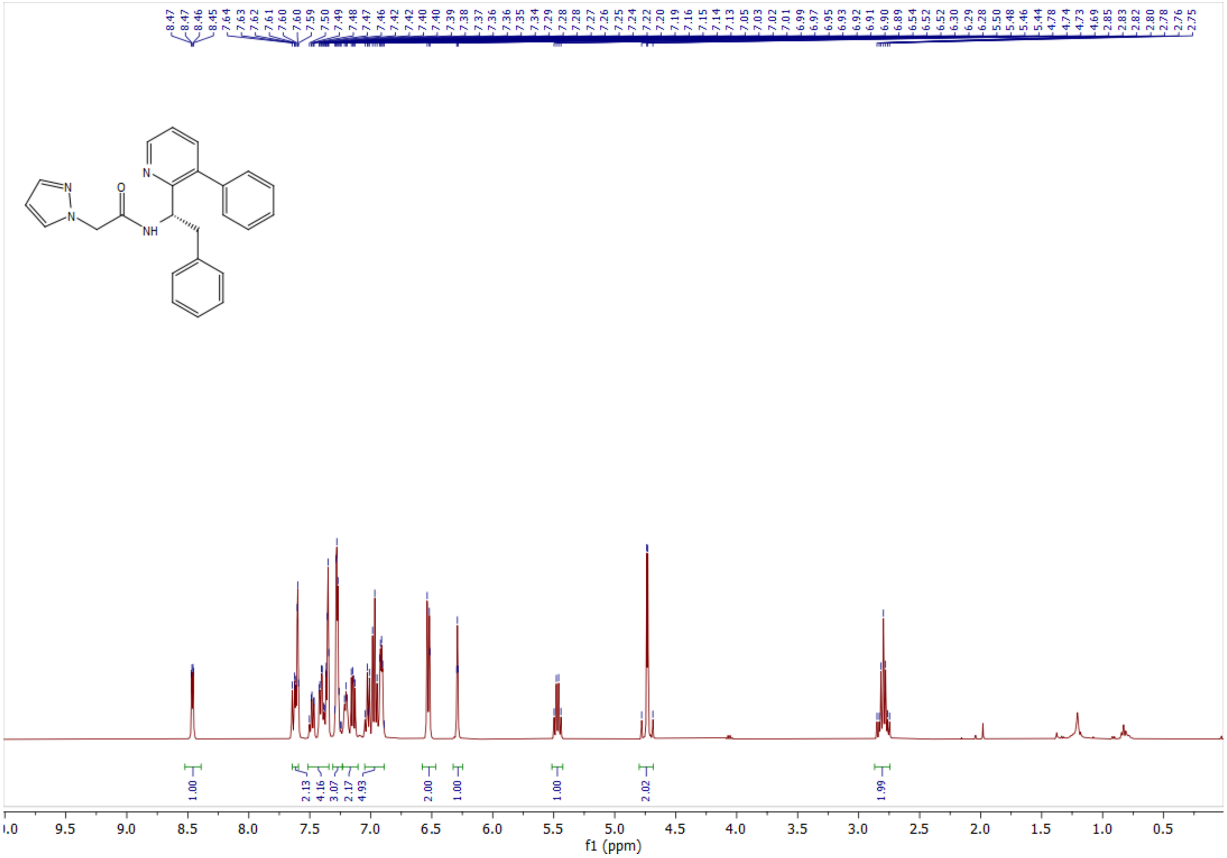
8.47 8.47 8.46 8.45 7.64 7.63 7.62 7.61 7.60 7.59 7.50 7.49 7.48 7.47 7.46 7.42 7.40 7.40 7.39 7.38 7.37 7.36 7.35 7.34 7.29 7.28 7.27 7.26 7.25 7.24 7.22 7.20 7.19 7.16 7.15 7.14 7.13 7.05 7.03 7.02 7.01 6.99 6.97 6.95 6.93 6.92 6.91 6.90 6.89 6.54 6.52 6.52 6.30 6.29 6.28 5.50 5.48 5.46 5.44 4.78 4.74 4.73 4.69 2.85 2.83 2.82 2.80 2.78 2.76 2.75
N
N
O
N
NH
1.00
2.13
4.16
3.07
2.17
4.93
2.00
1.00
1.00
2.02
1.99
f1 (ppm)

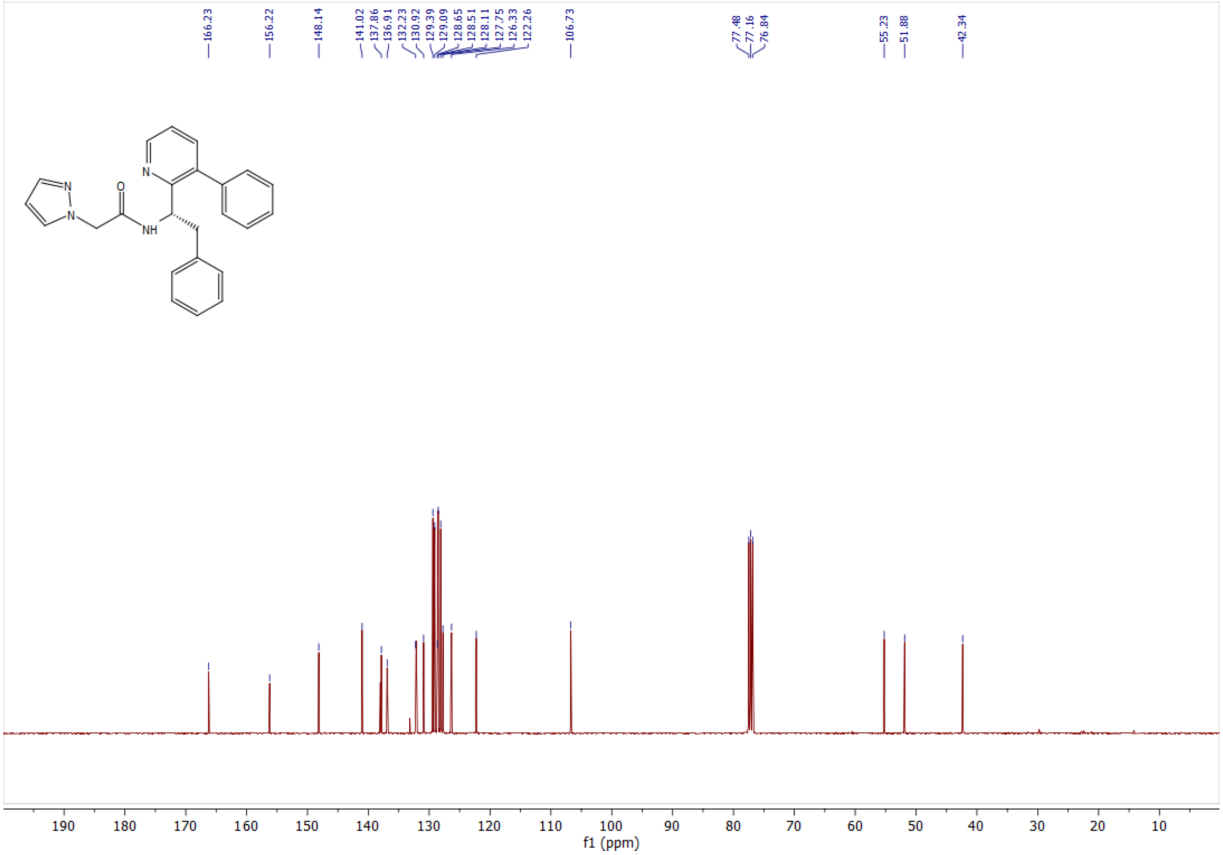
166.23
156.22
148.14
141.02 137.86 136.91 132.23 130.92 129.39 129.09 128.65 128.51 128.11 127.75 126.33 122.26
106.73
77.48 77.16 76.84
55.23
51.88
42.34
N
N
O
N
NH
f1 (ppm)

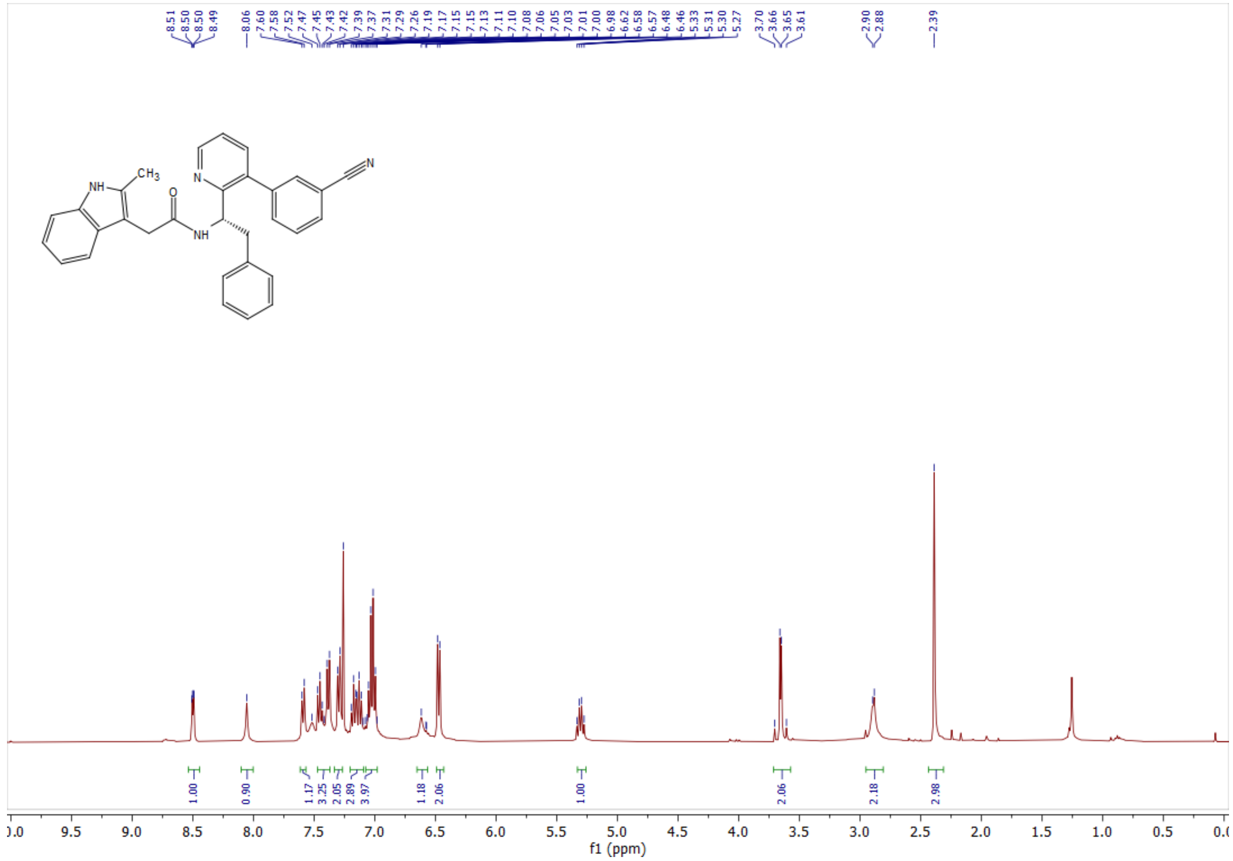

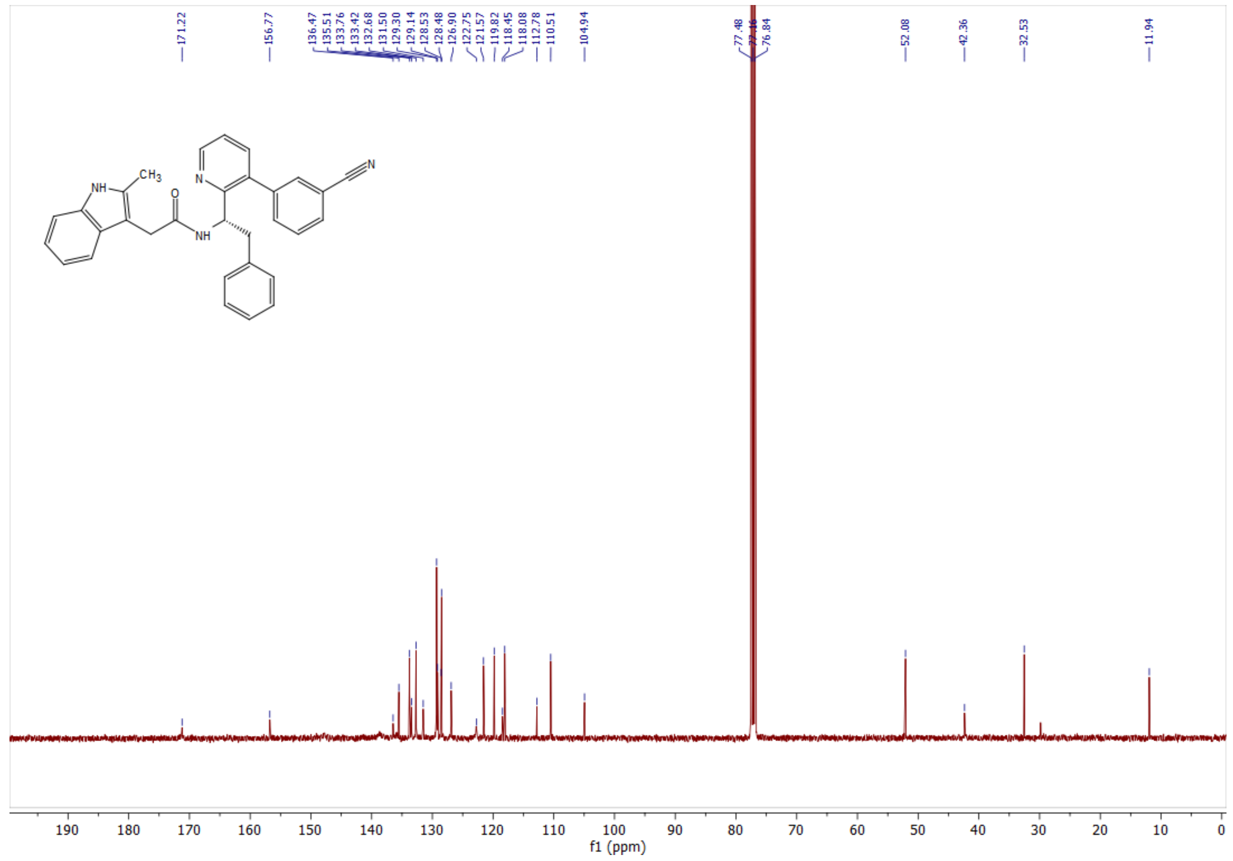

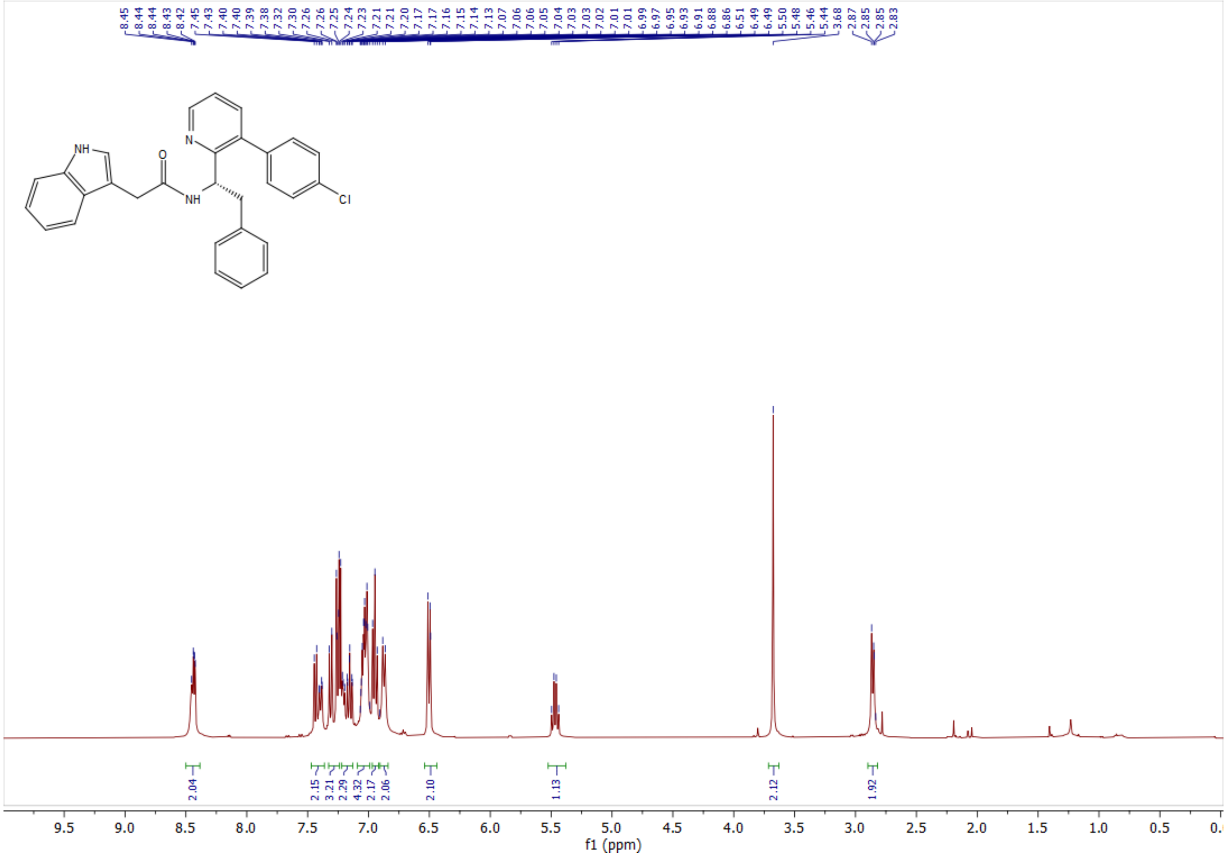

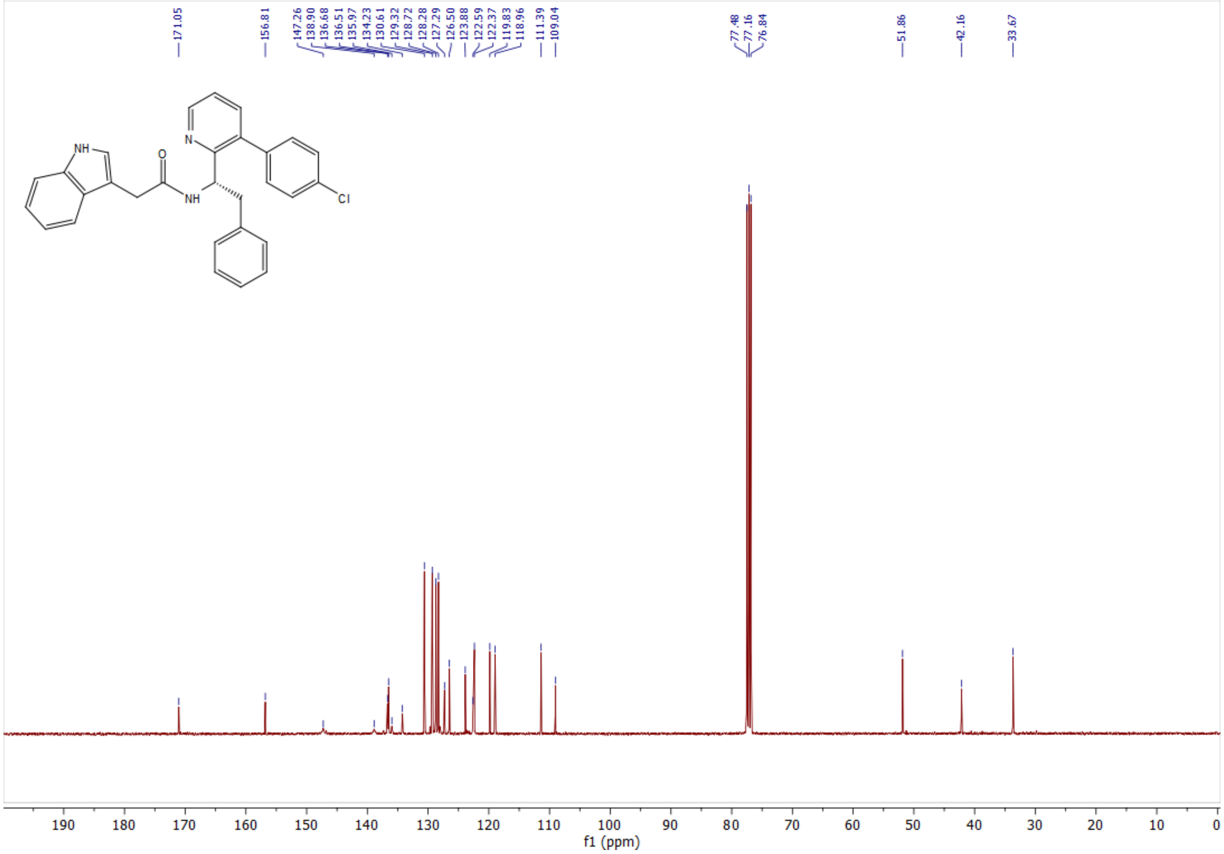

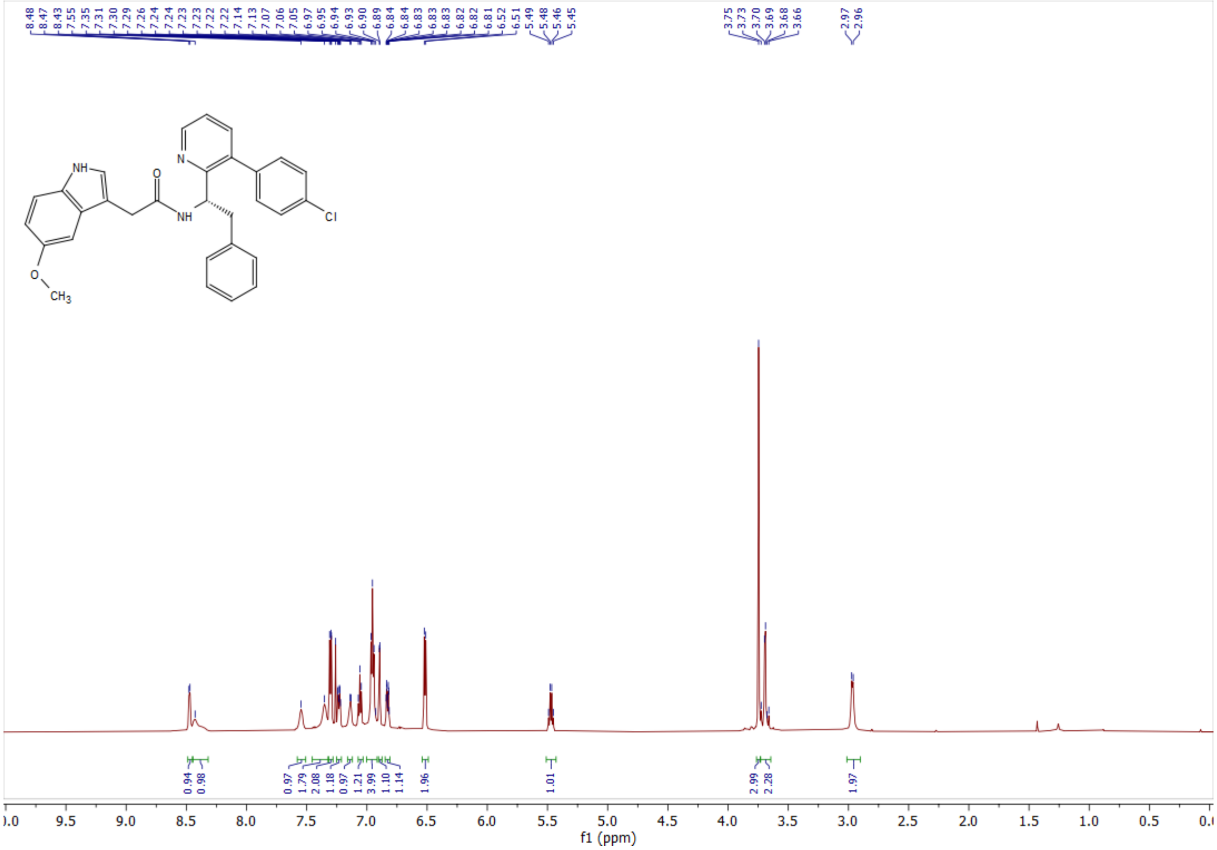
8.48
8.47
8.43
7.55
7.35
7.31
7.30
7.29
7.26
7.24
7.24
7.23
7.23
7.22
7.22
7.14
7.13
7.07
7.06
7.05
6.97
6.95
6.94
6.93
6.90
6.89
6.84
6.84
6.83
6.83
6.83
6.82
6.82
6.81
6.52
6.51
5.49
5.48
5.46
5.45
3.75
3.73
3.70
3.69
3.68
3.66
2.97
2.96
NH
O
N
NH
Cl
O
CH3
0.94
0.98
0.97
1.79
2.08
1.18
0.97
1.21
3.99
1.10
1.14
1.96
1.01
2.99
2.28
1.97
f1 (ppm)

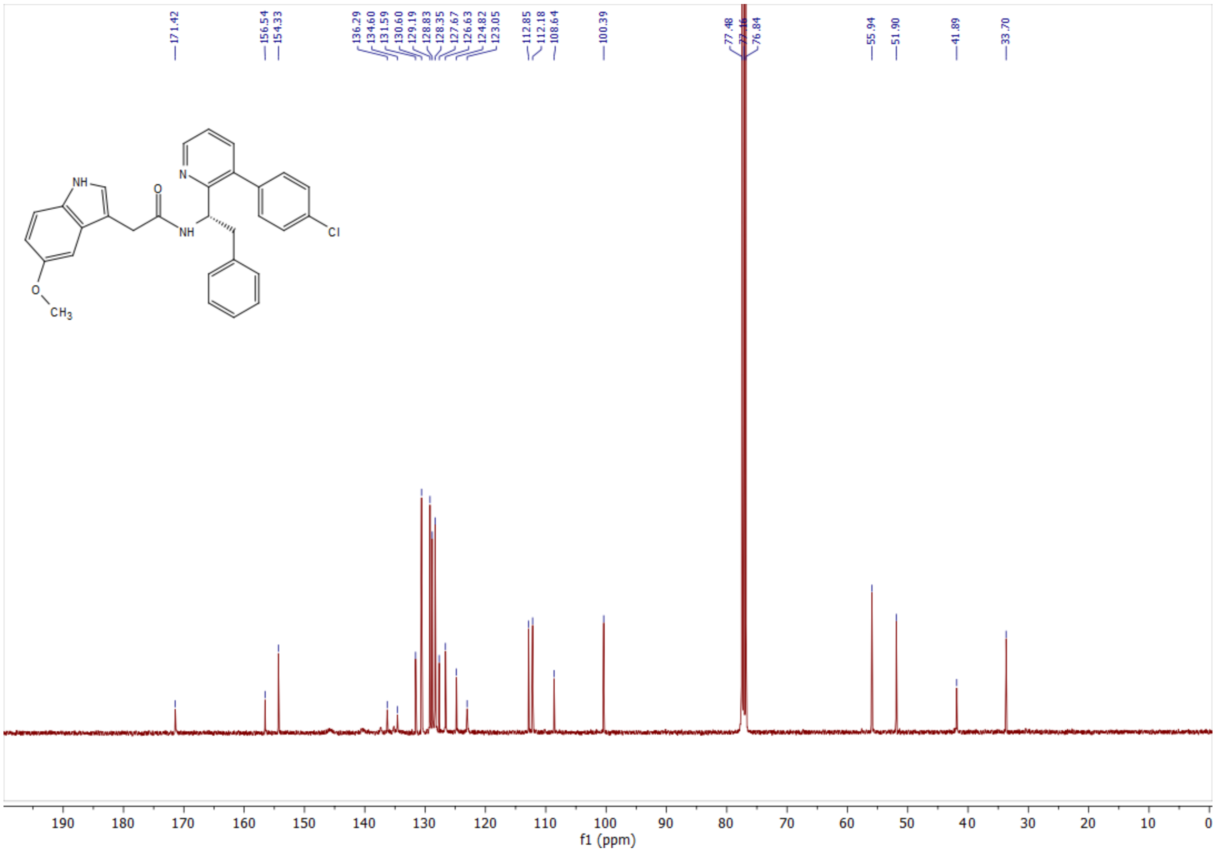
171.42
156.54
154.33
136.29
134.60
131.59
130.60
129.19
128.83
128.35
127.67
126.63
124.82
123.05
112.85
112.18
108.64
100.39
77.48
77.16
76.84
55.94
51.90
41.89
33.70
NH
O
N
NH
Cl
O
CH3
f1 (ppm)

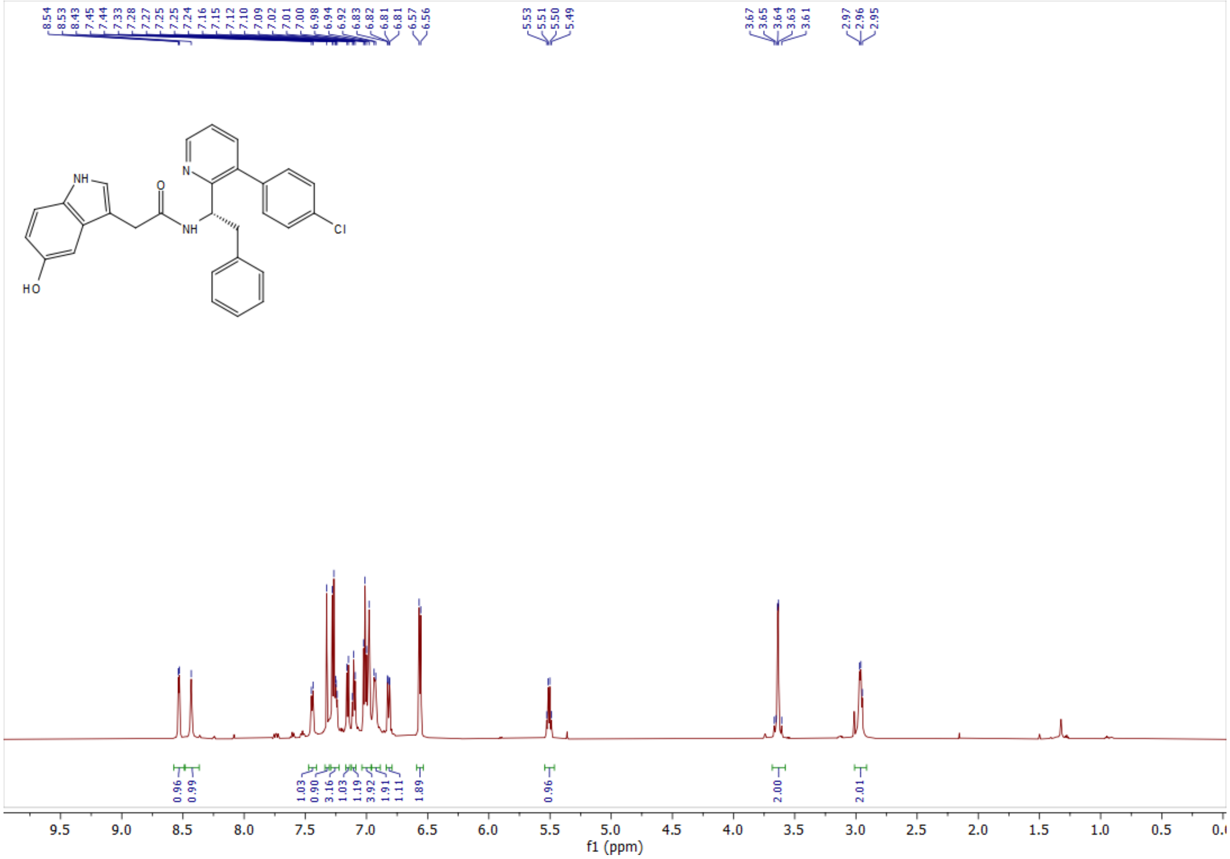
8.54
8.53
8.43
7.45
7.44
7.33
7.28
7.27
7.25
7.25
7.24
7.16
7.15
7.12
7.10
7.09
7.02
7.01
7.00
6.98
6.94
6.92
6.83
6.82
6.81
6.81
6.57
6.56
5.53
5.51
5.50
5.49
3.67
3.65
3.64
3.63
3.61
2.97
2.96
2.95
NH
O
N
NH
Cl
HO
0.96
0.99
1.03
0.90
3.16
1.03
1.19
3.92
1.91
1.11
1.89
0.96
2.00
2.01
f1 (ppm)

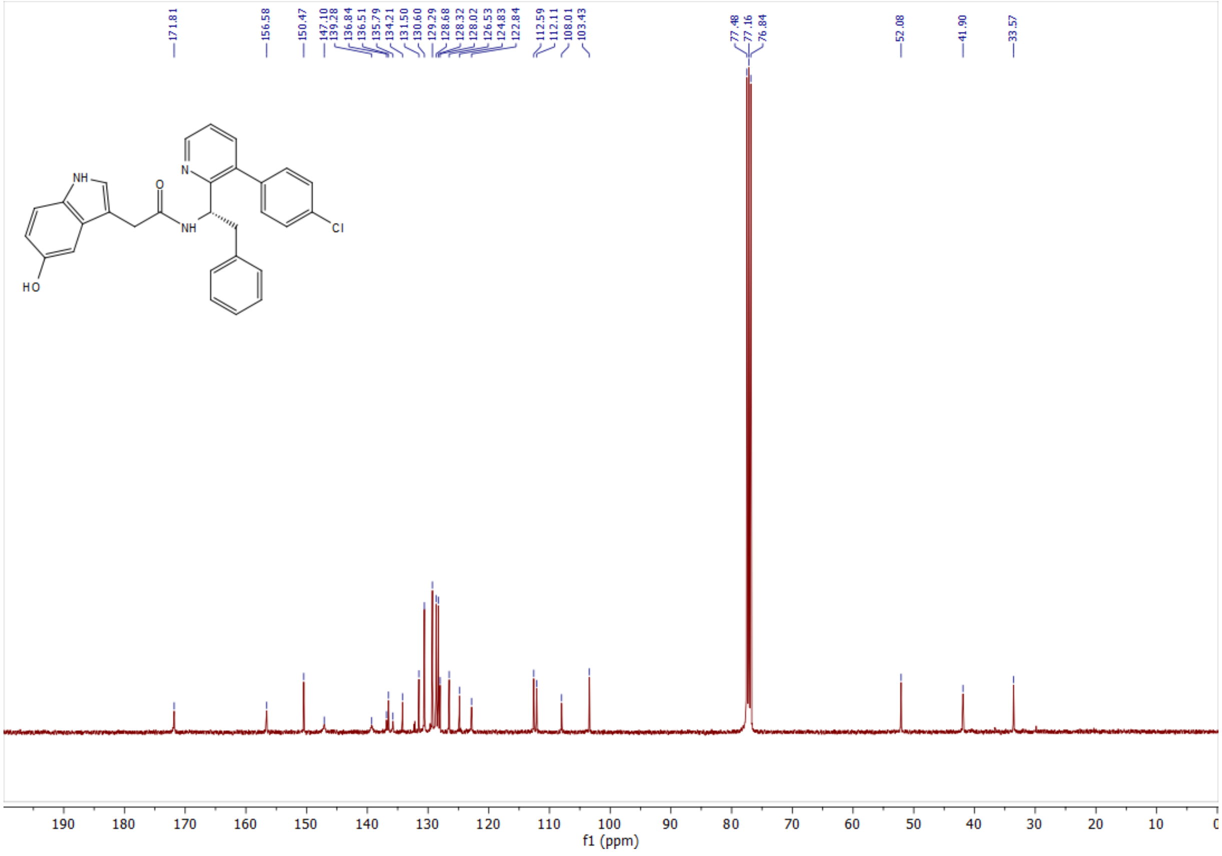
171.81
156.58
150.47
147.10
139.28
136.84
136.51
135.79
134.21
131.50
130.60
129.29
128.68
128.32
128.02
126.53
124.83
122.84
112.59
112.11
108.01
103.43
77.48
77.16
76.84
52.08
41.90
33.57
NH
O
N
NH
Cl
HO
f1 (ppm)

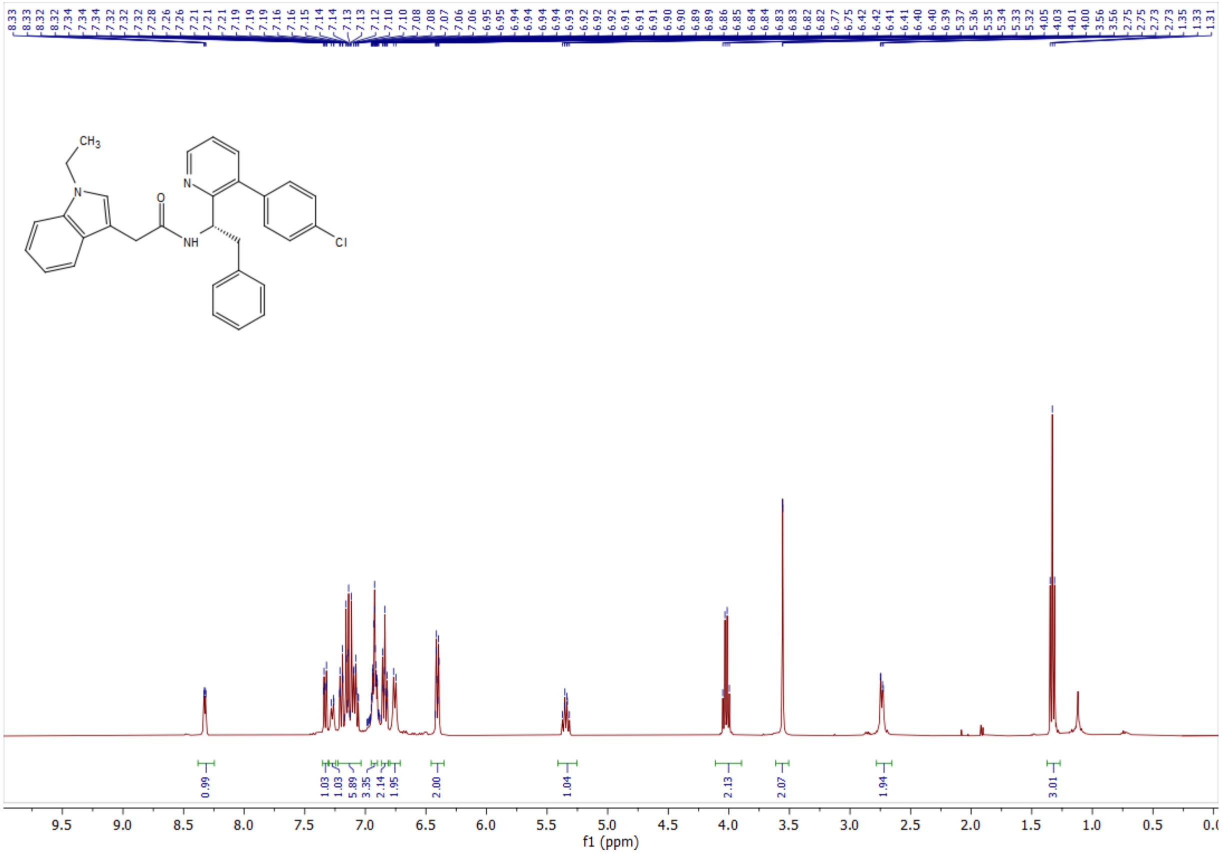

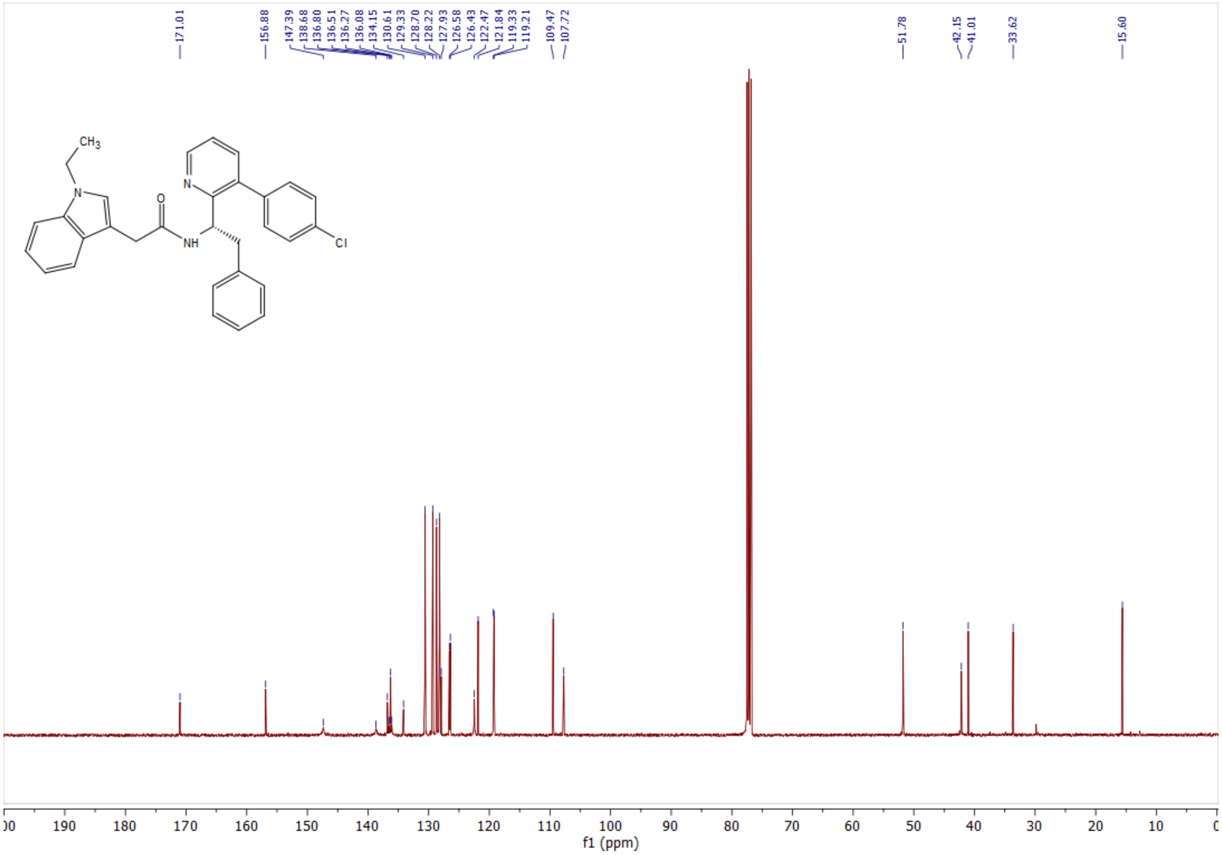

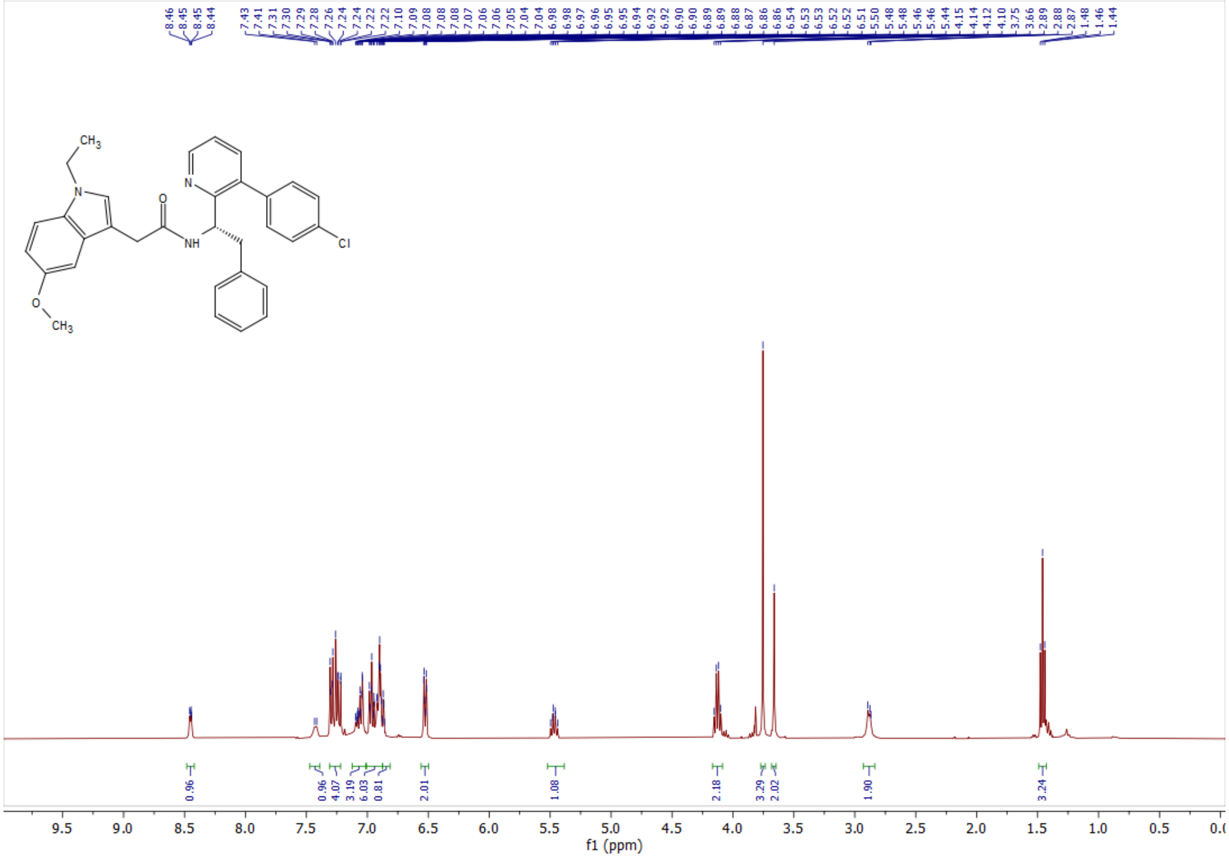
8.46
8.45
8.45
8.44
7.43
7.41
7.31
7.30
7.29
7.28
7.26
7.24
7.24
7.22
7.22
7.10
7.09
7.08
7.08
7.08
7.07
7.06
7.06
7.05
7.04
7.04
6.98
6.98
6.97
6.96
6.95
6.95
6.94
6.92
6.92
6.90
6.90
6.89
6.89
6.88
6.87
6.86
6.86
6.54
6.53
6.53
6.52
6.52
6.51
5.50
5.48
5.48
5.46
5.44
4.15
4.14
4.12
4.10
3.75
3.66
2.89
2.88
2.87
1.48
1.46
1.44
0.96
0.96
4.07
3.19
6.03
0.81
2.01
1.08
2.18
3.29
2.02
1.90
3.24
f1 (ppm)

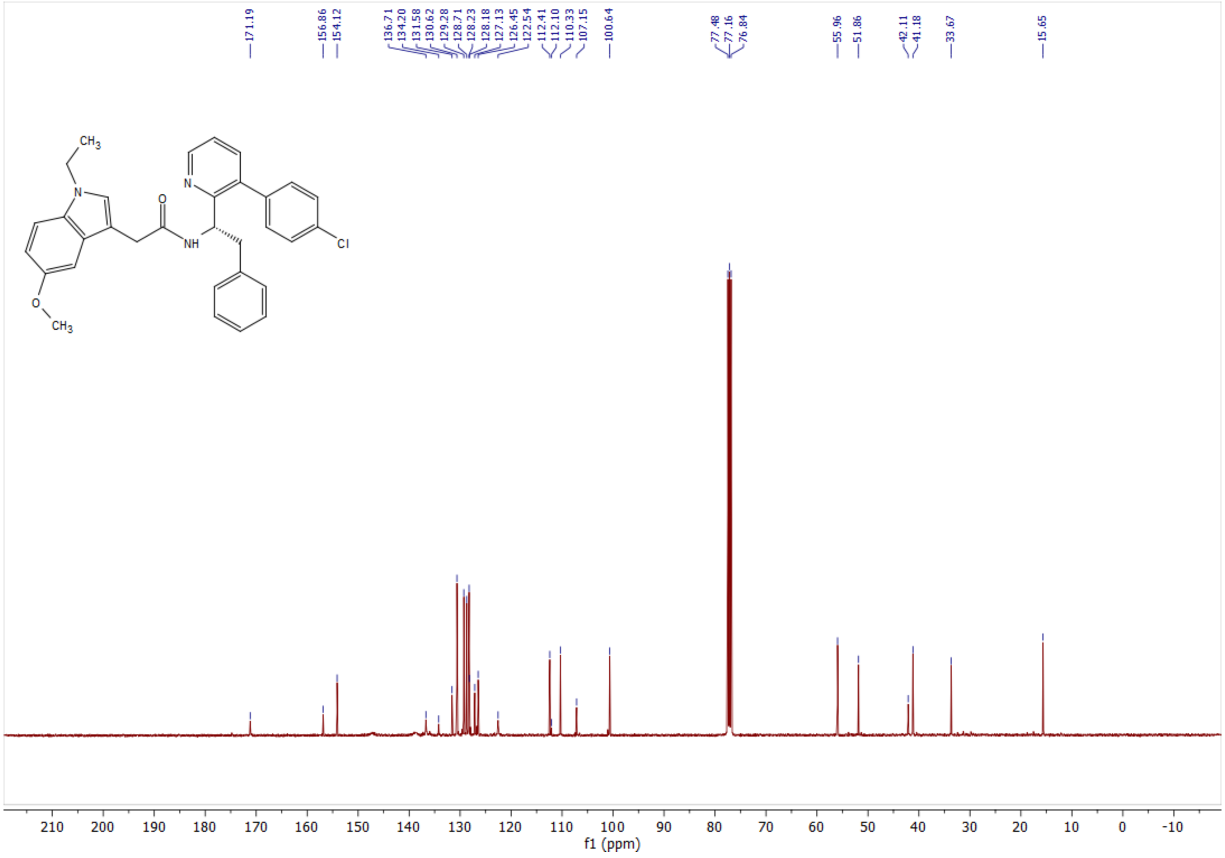
171.19
156.86
154.12
136.71
134.20
131.58
130.62
129.28
128.71
128.23
128.18
127.13
126.45
122.54
112.41
112.10
110.33
107.15
100.64
77.48
77.16
76.84
55.96
51.86
42.11
41.18
33.67
15.65
f1 (ppm)

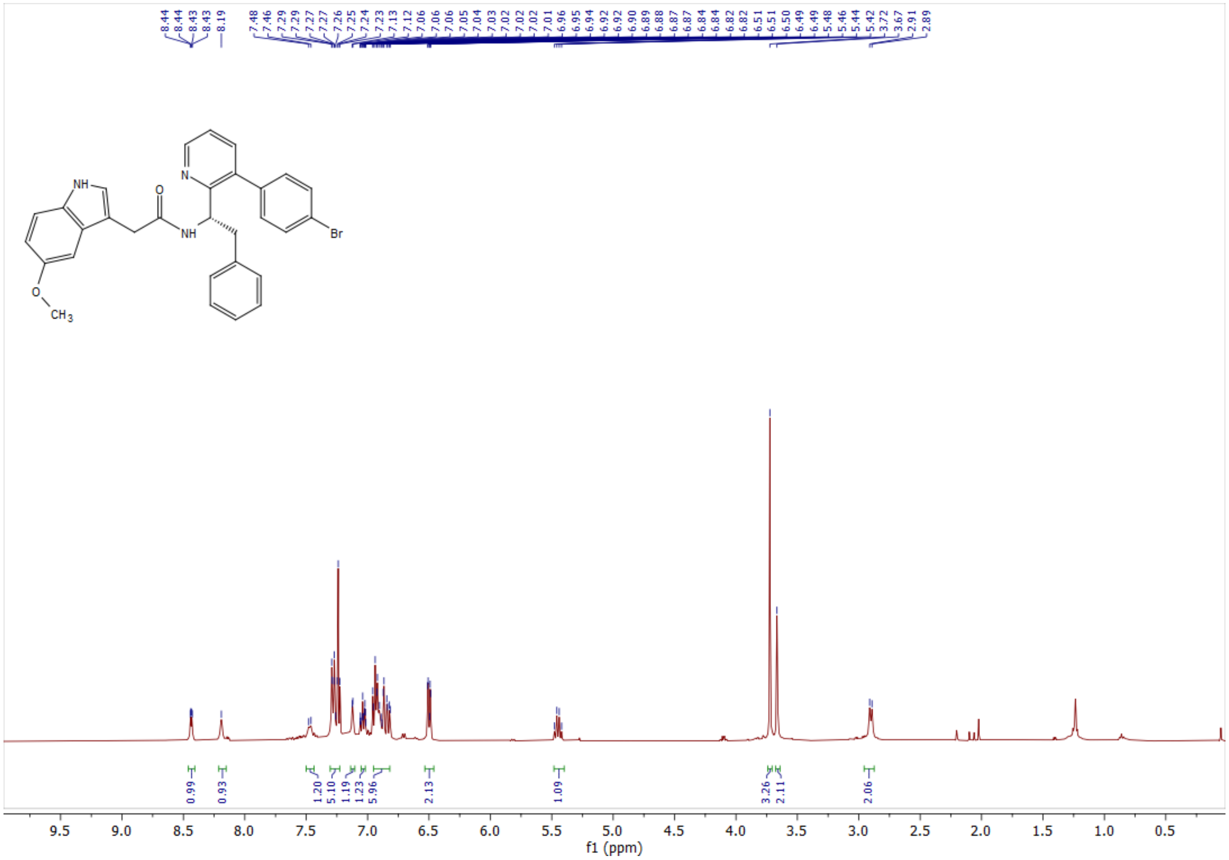

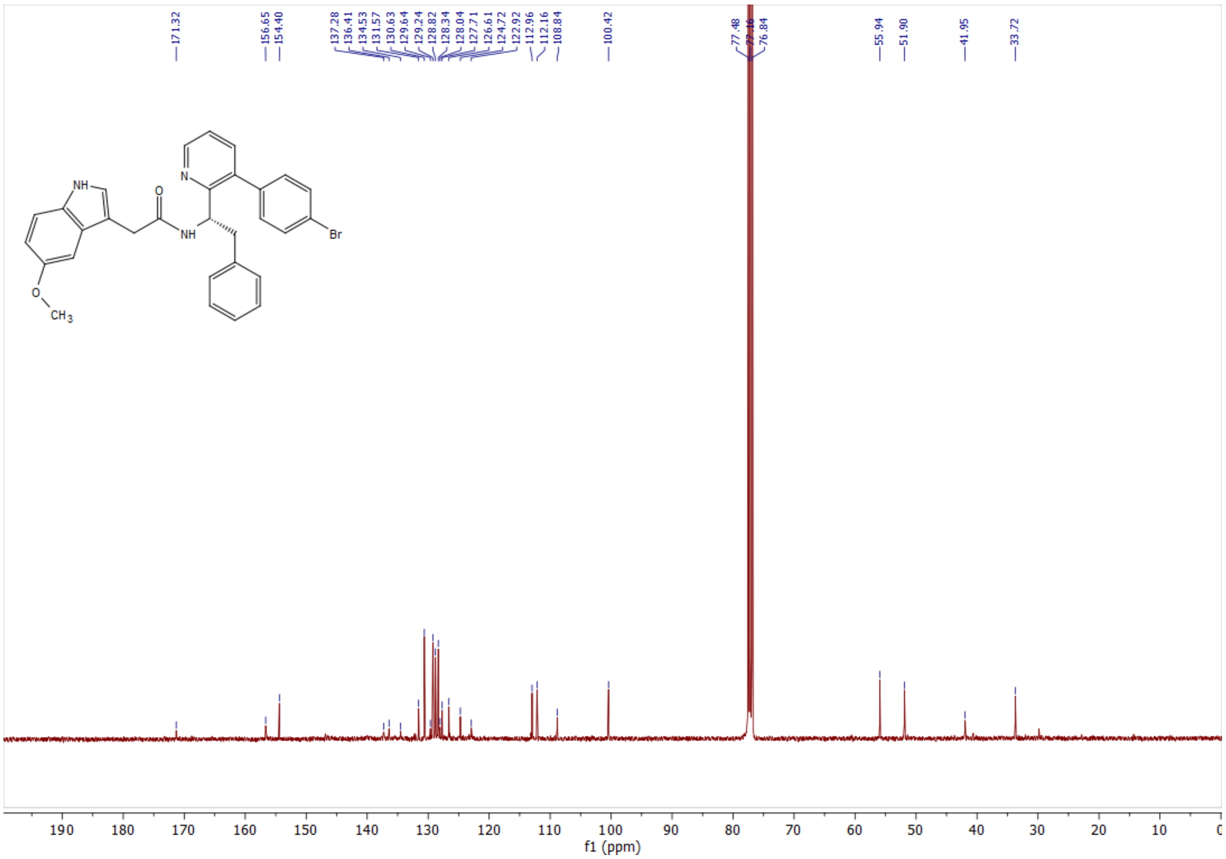

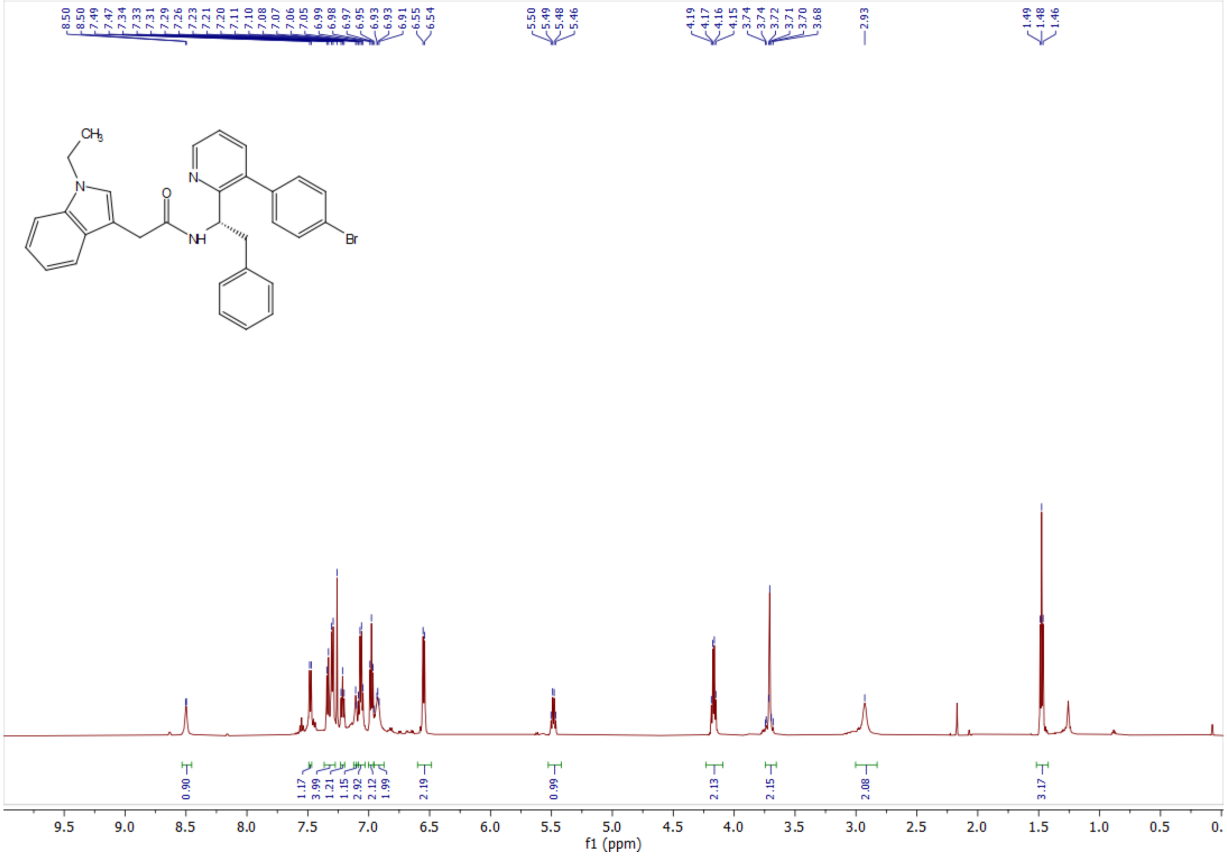

8.50
8.50
7.49
7.47
7.34
7.33
7.31
7.29
7.26
7.23
7.21
7.20
7.11
7.10
7.08
7.07
7.06
7.05
6.99
6.98
6.97
6.95
6.93
6.93
6.91
6.55
6.54
5.50
5.49
5.48
5.46
4.19
4.17
4.16
4.15
3.74
3.74
3.72
3.71
3.70
3.68
2.93
1.49
1.48
1.46
CH3
N
O
NH
N
Br
0.90
1.17
3.99
1.21
1.15
2.92
2.12
1.99
2.19
0.99
2.13
2.15
2.08
3.17
9.5
9.0
8.5
8.0
7.5
7.0
6.5
6.0
5.5
5.0
4.5
4.0
3.5
3.0
2.5
2.0
1.5
1.0
0.5
0.
f1 (ppm)

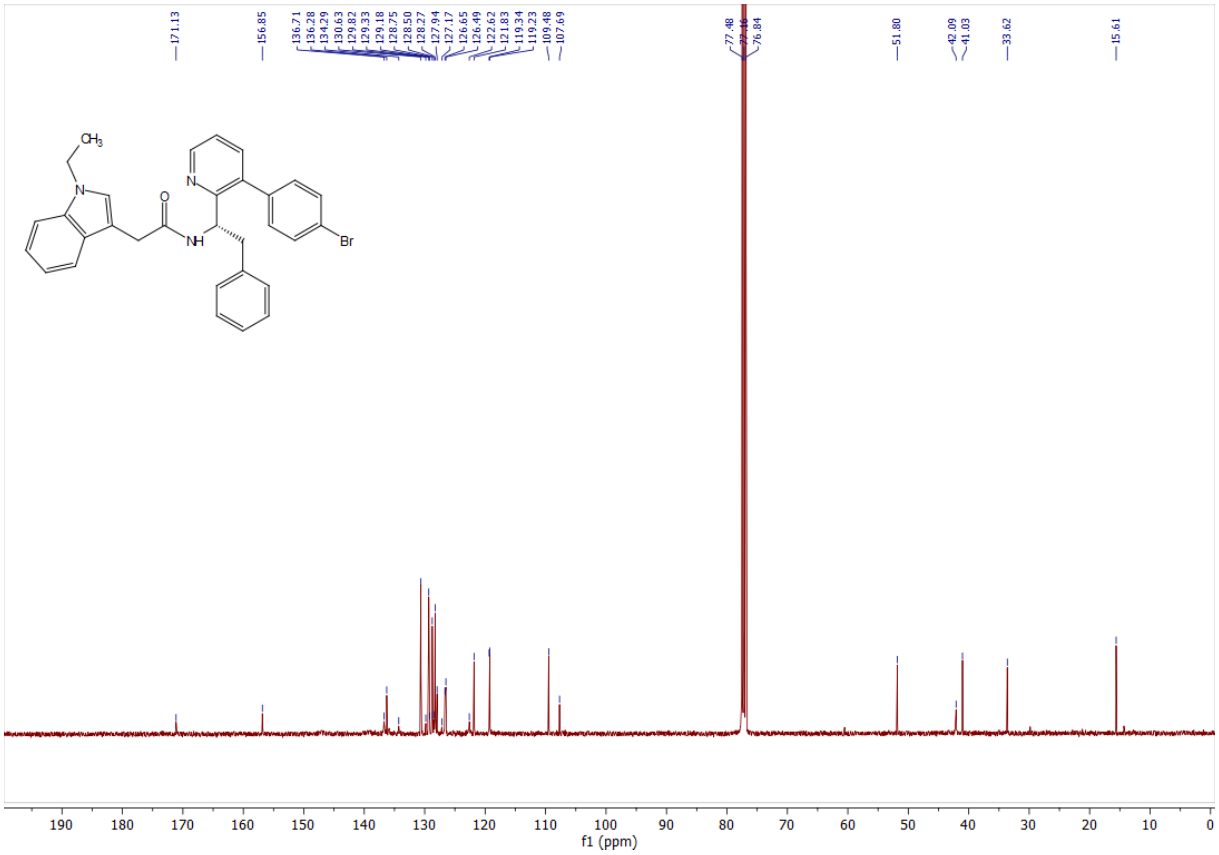

171.13
156.85
136.71
136.28
134.29
130.63
129.82
129.33
129.18
128.75
128.50
128.27
127.94
127.17
126.65
126.49
122.62
121.83
119.34
119.23
109.48
107.69
77.48
76.84
51.80
42.09
41.03
33.62
15.61
CH3
N
O
NH
N
Br
190
180
170
160
150
140
130
120
110
100
90
80
70
60
50
40
30
20
10
0
f1 (ppm)

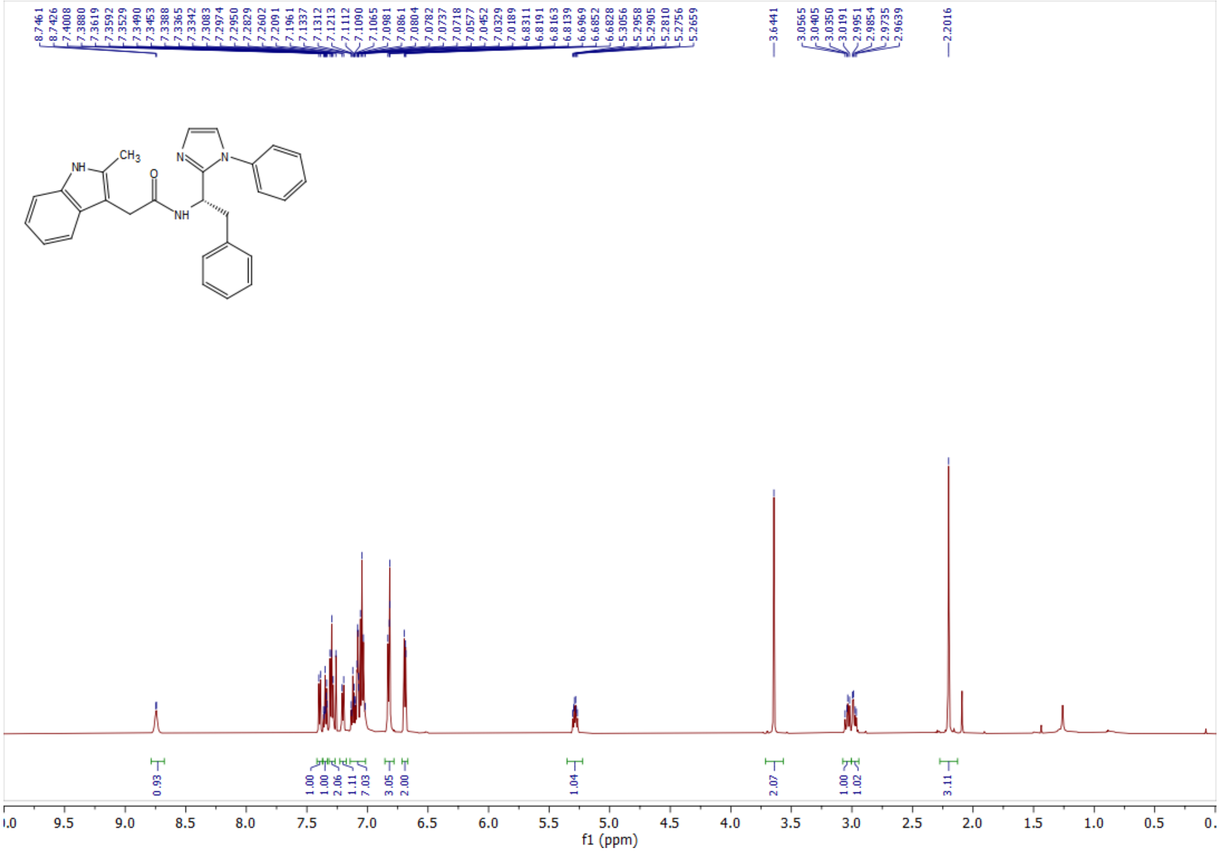
f1 (ppm)

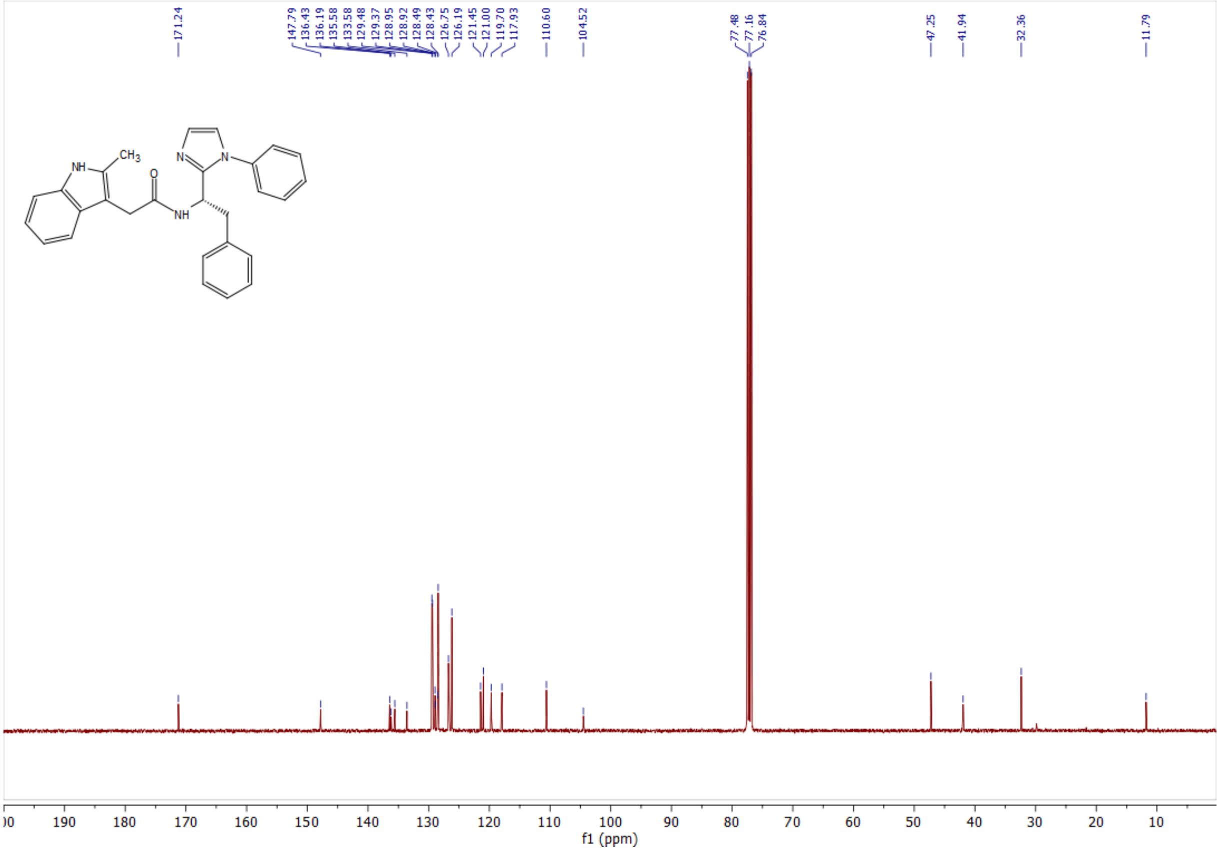
f1 (ppm)

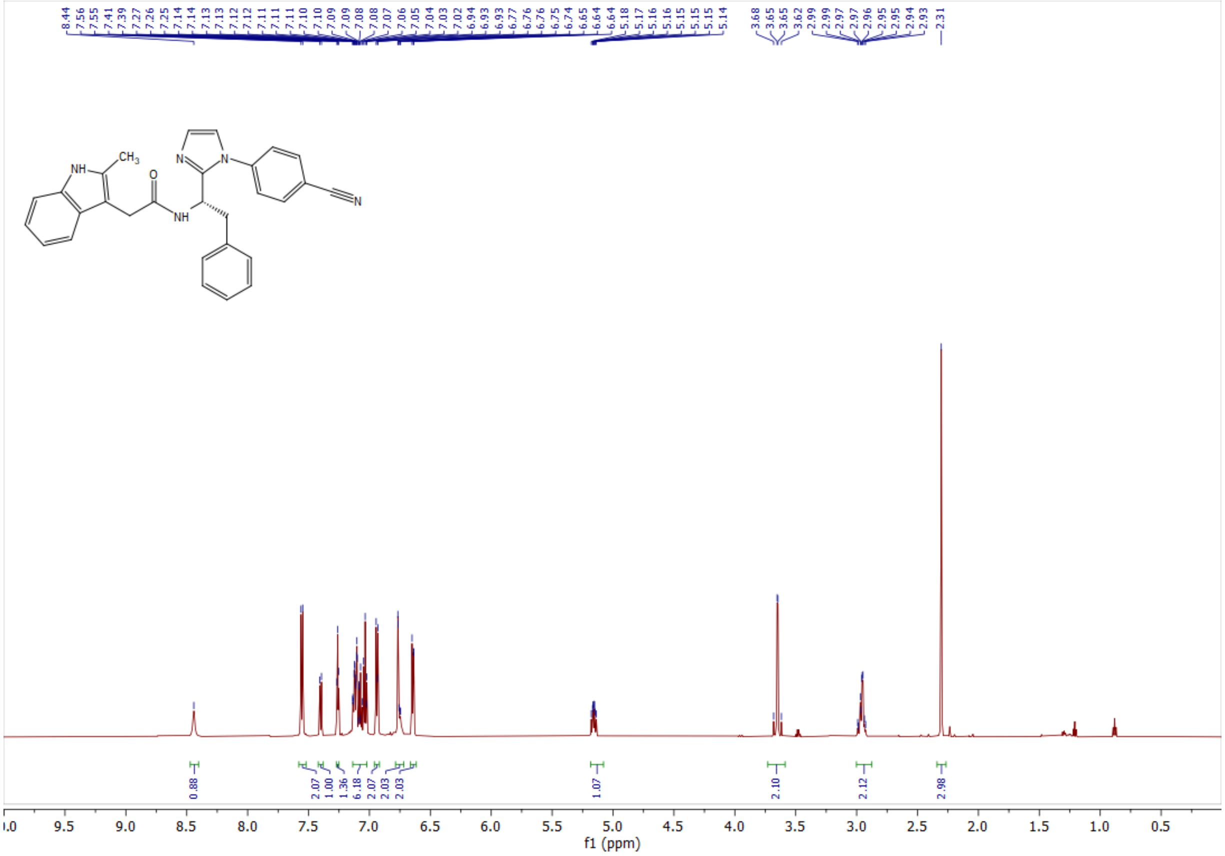

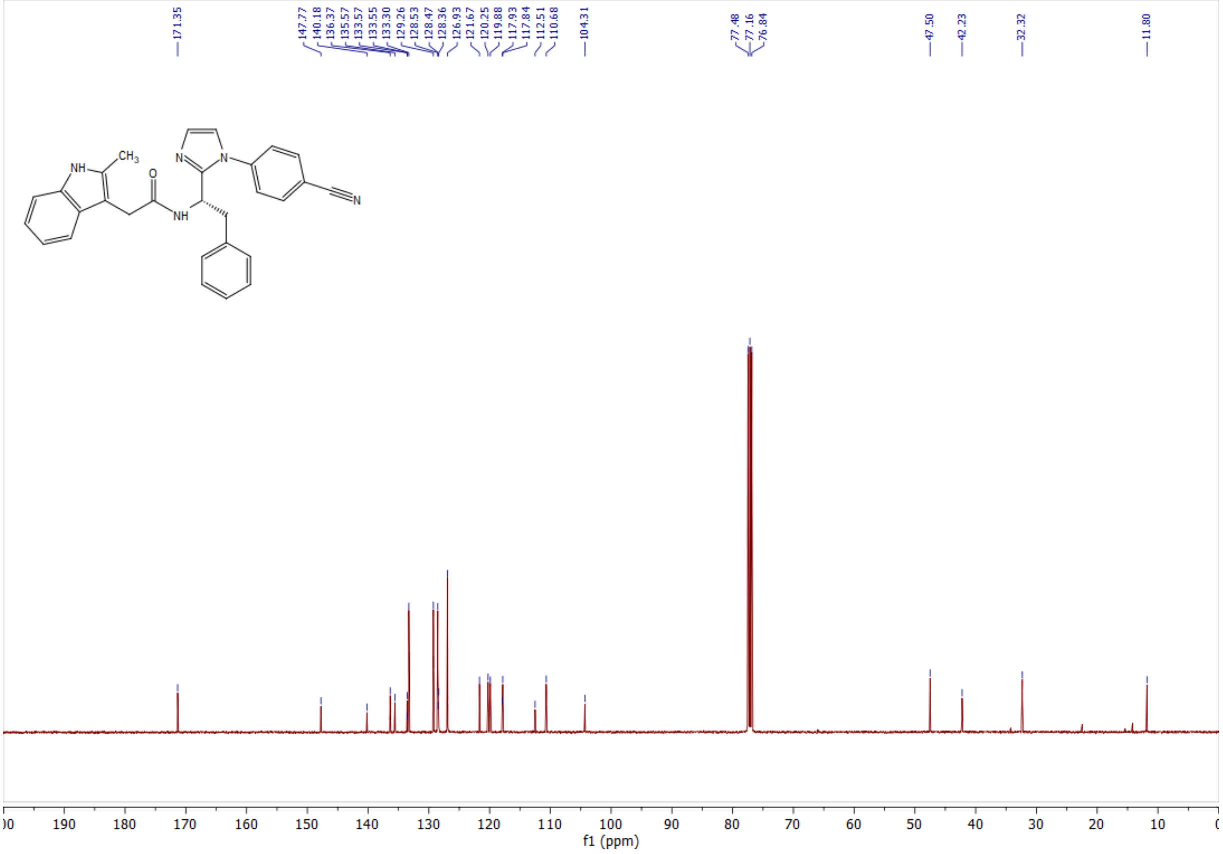

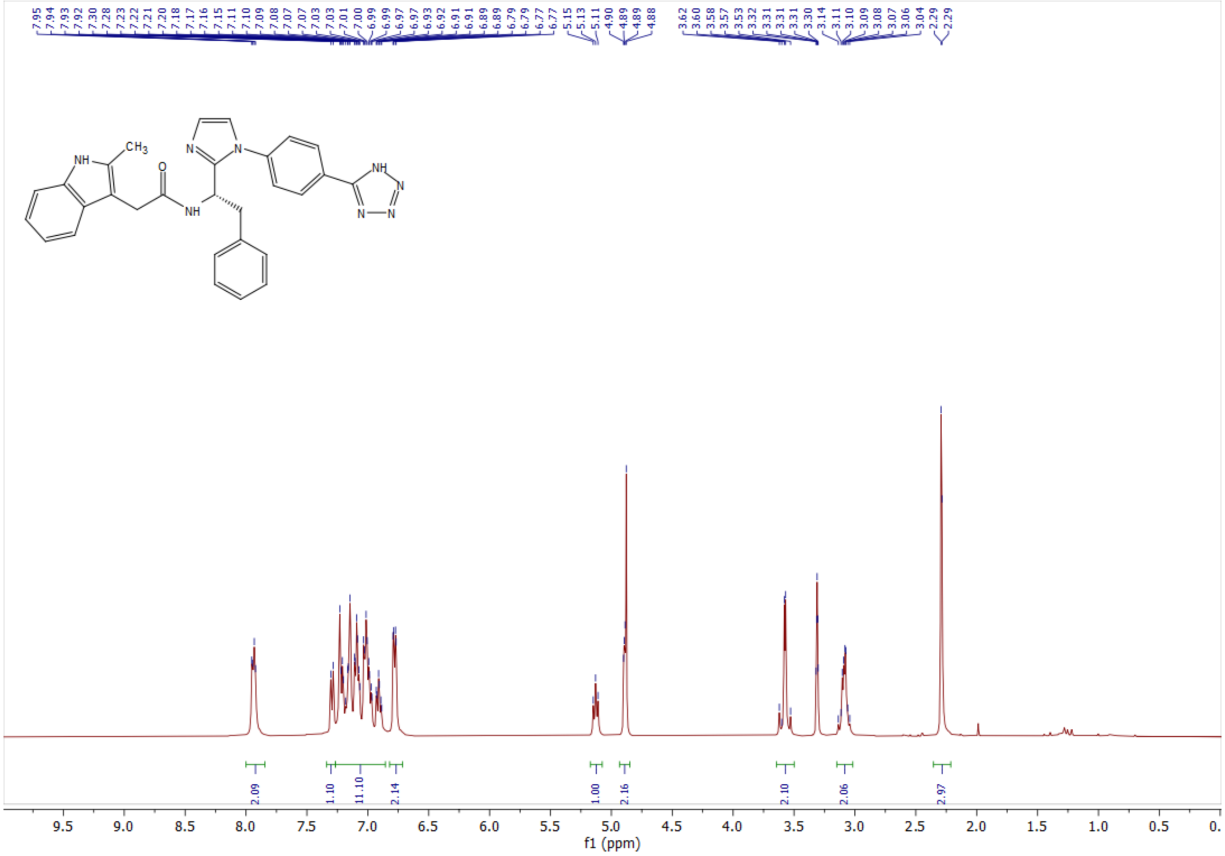

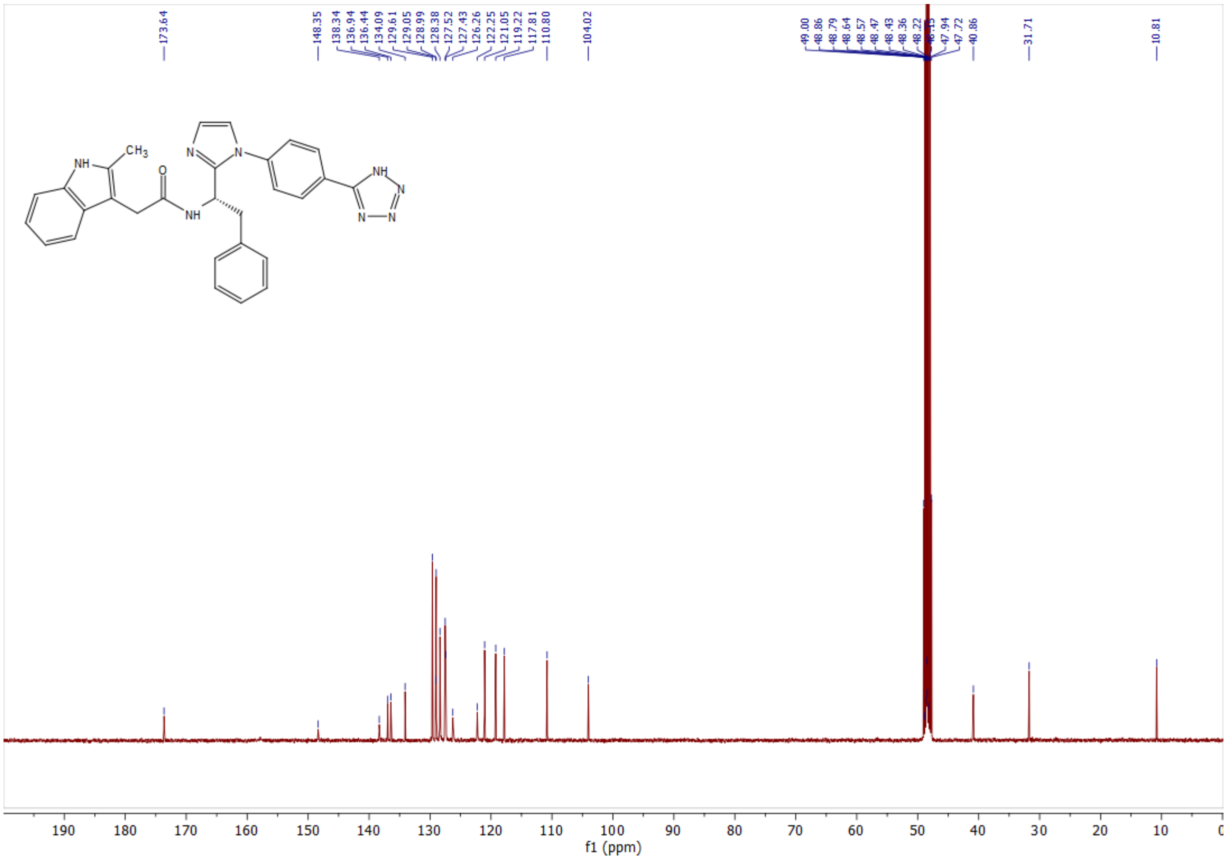

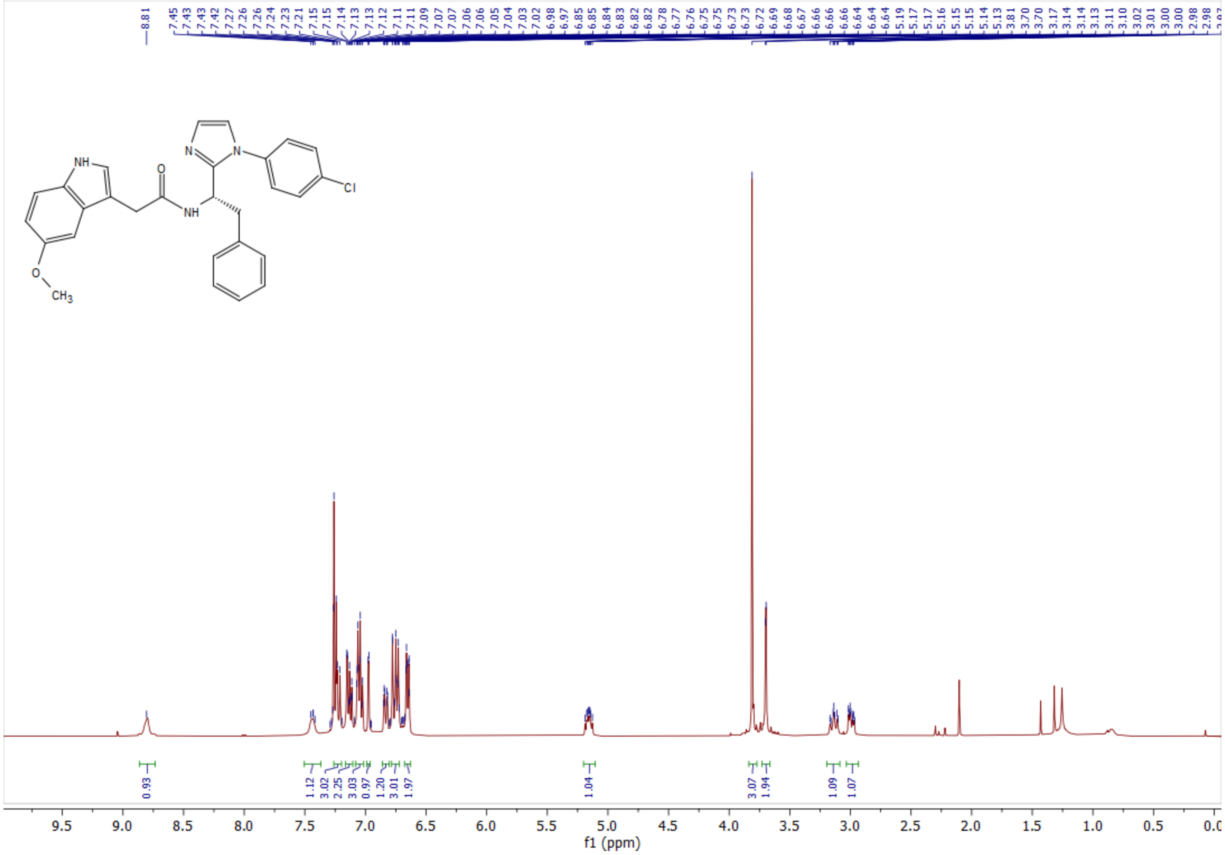

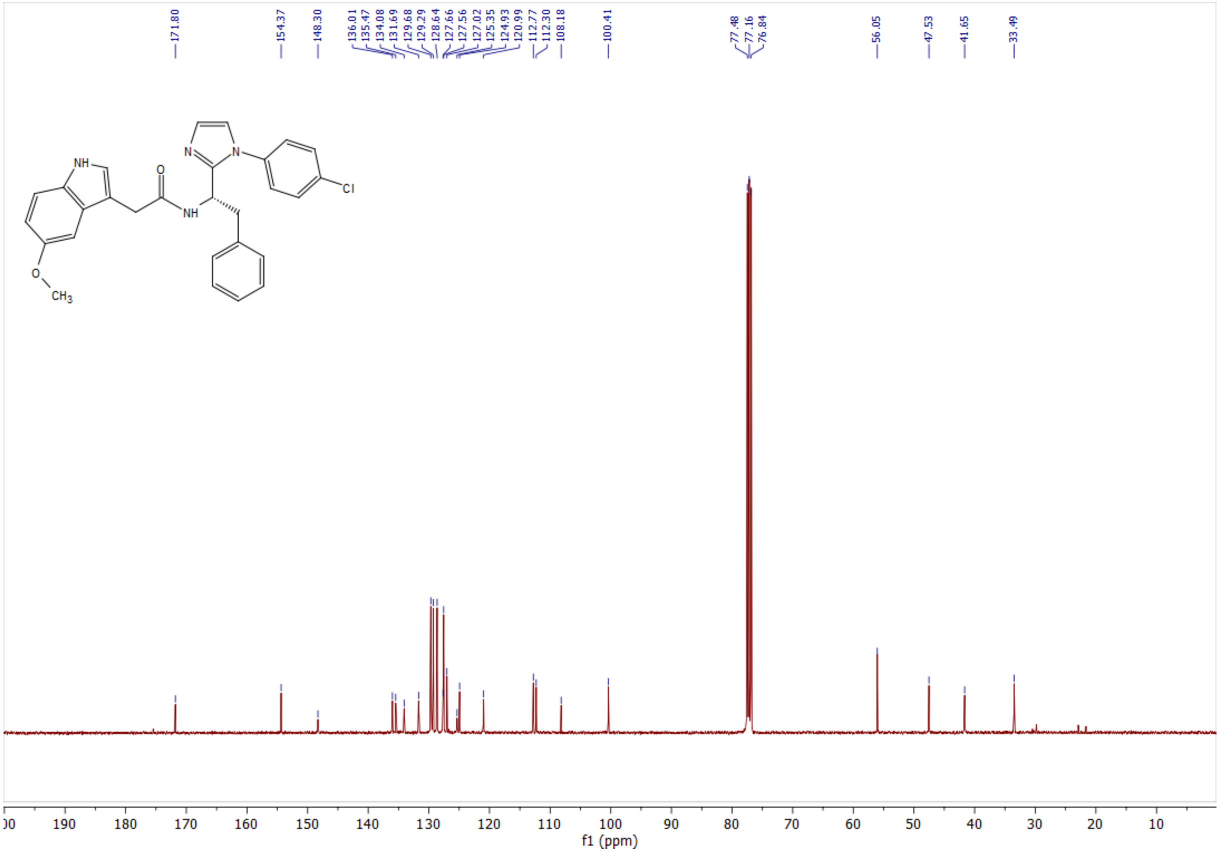

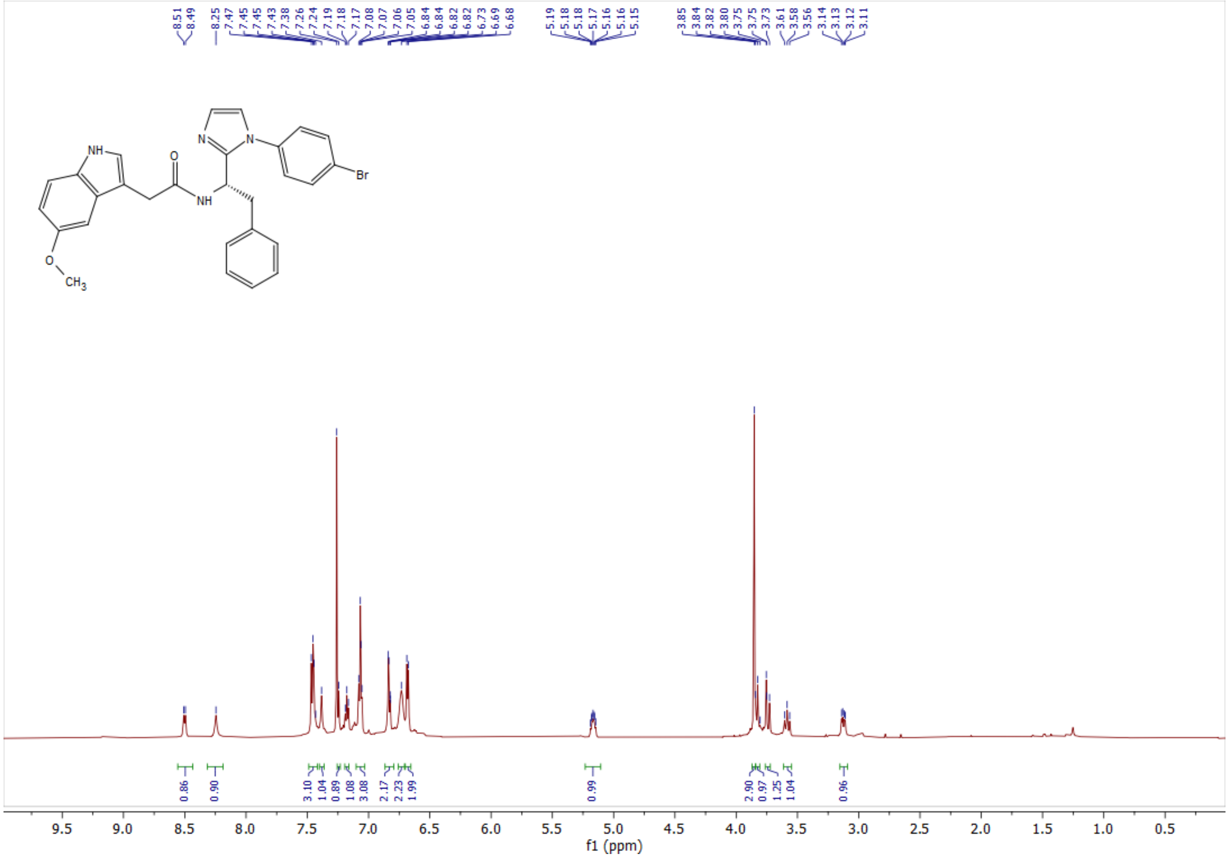

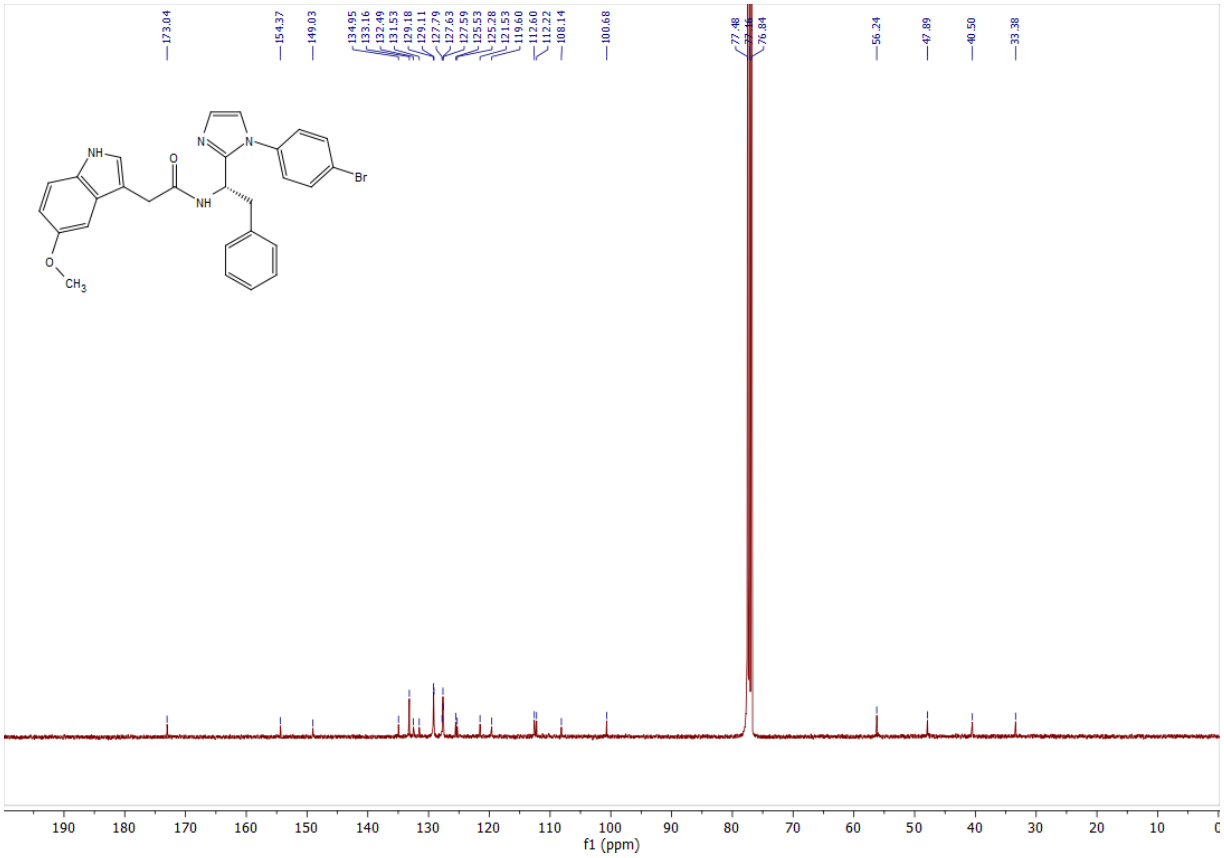

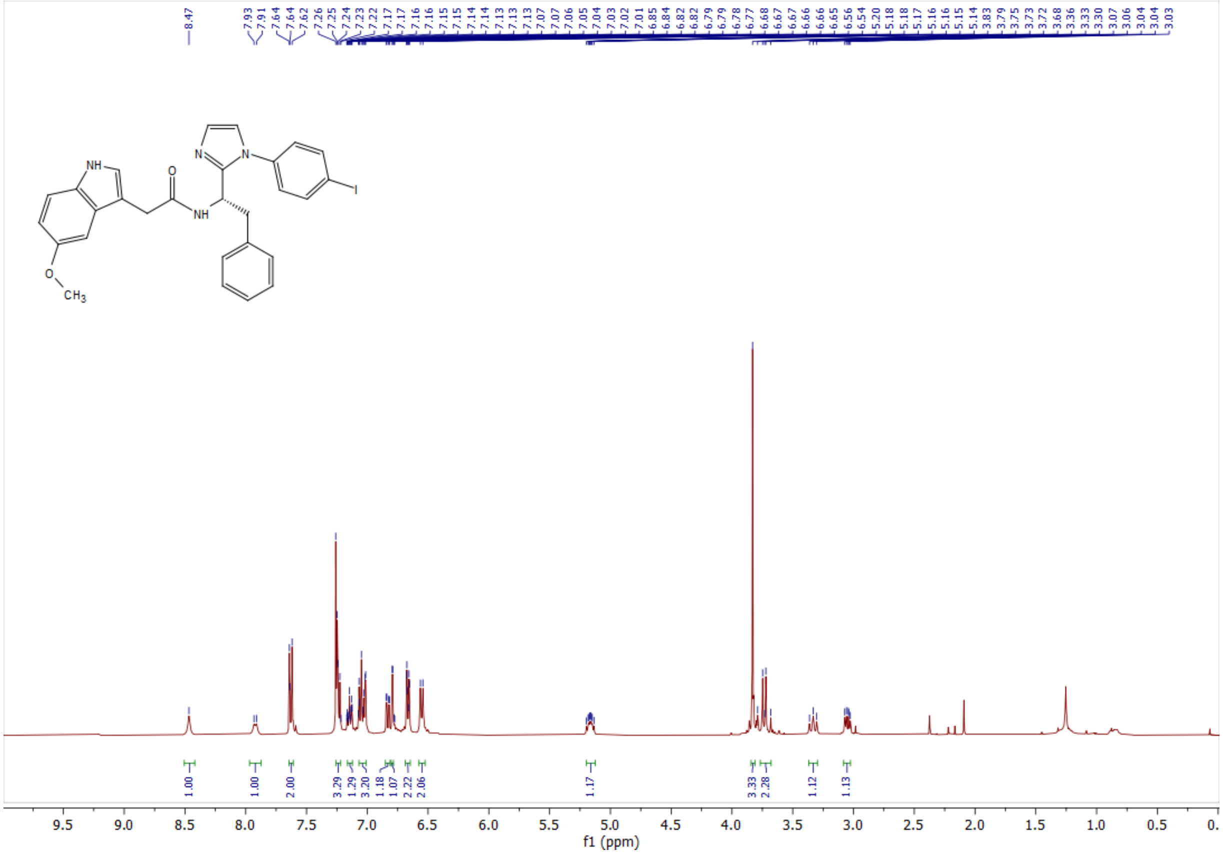

8.47
7.93
7.91
7.64
7.62
1.00
1.00
2.00
3.29
1.29
3.20
1.18
1.07
2.22
2.06
1.17
3.33
2.28
1.12
1.13
f1 (ppm)

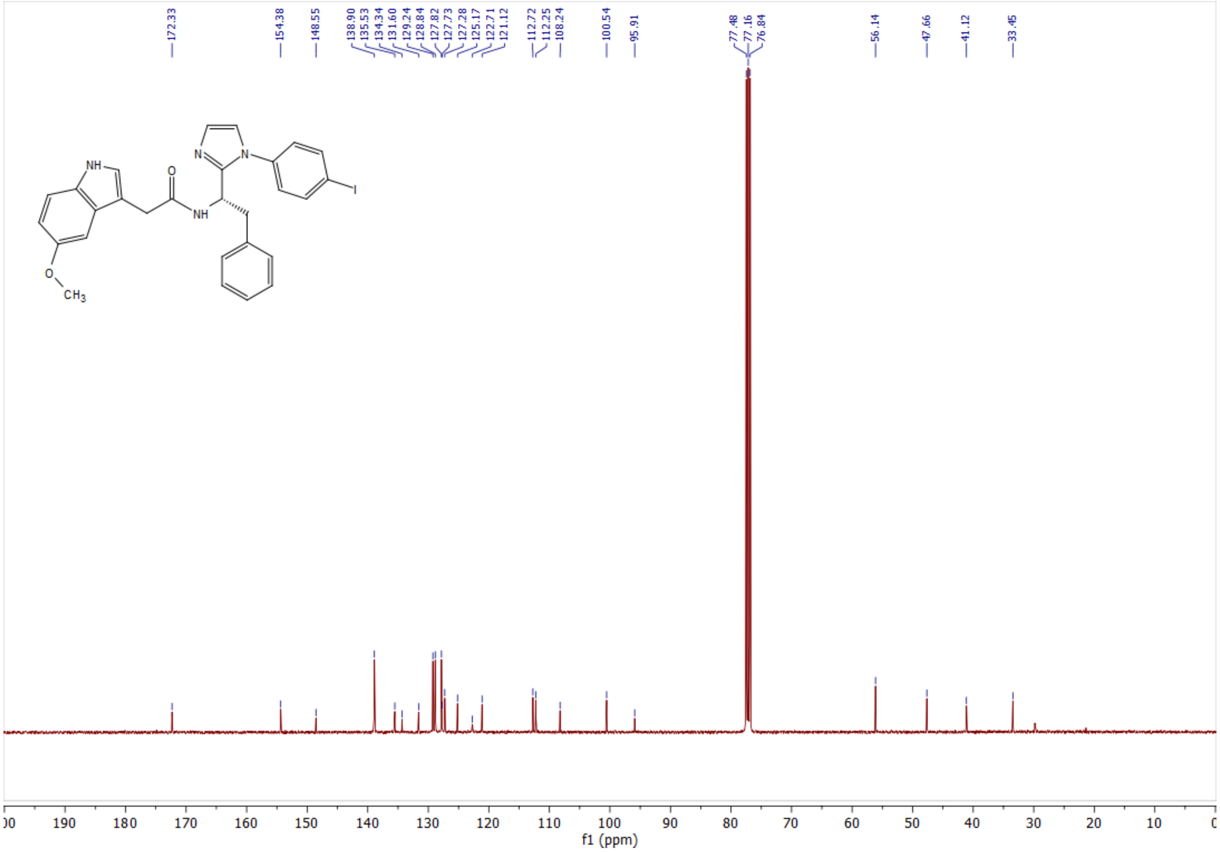

172.33
154.38
148.55
138.90
135.53
134.34
131.60
129.24
128.84
127.82
127.73
127.28
125.17
122.71
121.12
112.72
112.25
108.24
100.54
95.91
77.48
77.16
76.84
56.14
47.66
41.12
33.45
f1 (ppm)
